# Robust dimethyl‐based multiplex‐DIA doubles single‐cell proteome depth via a reference channel

**DOI:** 10.15252/msb.202211503

**Published:** 2023-08-21

**Authors:** Marvin Thielert, Ericka CM Itang, Constantin Ammar, Florian A Rosenberger, Isabell Bludau, Lisa Schweizer, Thierry M Nordmann, Patricia Skowronek, Maria Wahle, Wen‐Feng Zeng, Xie‐Xuan Zhou, Andreas‐David Brunner, Sabrina Richter, Mitchell P Levesque, Fabian J Theis, Martin Steger, Matthias Mann

**Affiliations:** ^1^ Department of Proteomics and Signal Transduction Max Planck Institute of Biochemistry Martinsried Germany; ^2^ Boehringer Ingelheim Pharma GmbH & Co. KG, Drug Discovery Sciences Biberach an der Riss Germany; ^3^ Helmholtz Zentrum München – German Research Center for Environmental Health Institute of Computational Biology Neuherberg Germany; ^4^ TUM School of Life Sciences Weihenstephan Technical University of Munich Freising Germany; ^5^ Department of Dermatology University of Zurich, University of Zurich Hospital Zurich Switzerland; ^6^ New address: NEOsphere Biotechnologies GmbH Planegg Germany; ^7^ Novo Nordisk Foundation Center for Protein Research, Faculty of Health and Medical Sciences University of Copenhagen Copenhagen Denmark

**Keywords:** DIA, dimethyl labeling, multiplexing, single cells, spatial proteomics, Methods & Resources, Proteomics

## Abstract

Single‐cell proteomics aims to characterize biological function and heterogeneity at the level of proteins in an unbiased manner. It is currently limited in proteomic depth, throughput, and robustness, which we address here by a streamlined multiplexed workflow using data‐independent acquisition (mDIA). We demonstrate automated and complete dimethyl labeling of bulk or single‐cell samples, without losing proteomic depth. Lys‐N digestion enables five‐plex quantification at MS1 and MS2 level. Because the multiplexed channels are quantitatively isolated from each other, mDIA accommodates a reference channel that does not interfere with the target channels. Our algorithm RefQuant takes advantage of this and confidently quantifies twice as many proteins per single cell compared to our previous work (Brunner *et al*, PMID 35226415), while our workflow currently allows routine analysis of 80 single cells per day. Finally, we combined mDIA with spatial proteomics to increase the throughput of Deep Visual Proteomics seven‐fold for microdissection and four‐fold for MS analysis. Applying this to primary cutaneous melanoma, we discovered proteomic signatures of cells within distinct tumor microenvironments, showcasing its potential for precision oncology.

## Graphical abstract

A robust and automated multiplexed DIA (mDIA) workflow is presented, using complete dimethyl labeling for bulk or single‐cell proteomics. Accurate quantification with a reference channel, combined with the RefQuant algorithm, confirms the hypothesis of a stable single‐cell proteome.

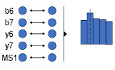

## Introduction

Characterizing biology directly at the single‐cell level is greatly advancing our knowledge of different cell types and cellular heterogeneity. Single‐cell RNA sequencing (scRNAseq) is now routine and large atlases of human cell populations of different organs are being generated (Eraslan *et al*, 2022; Suo *et al*, 2022; Tabula Sapiens Consortium *et al*, 2022). To clearly define single cell types or sub‐types, such measurements often encompass tens or hundreds of thousands of single‐cell transcriptomes.

Single‐cell mass spectrometry (MS)‐based proteomics (scProteomics) is also generating much interest, because the dynamic proteome is thought to be a very informative reflection of the biological function of cells. Furthermore, scProteomics could complement other omics modalities in a multi‐level description of cellular systems. However, this requires overcoming key technological challenges in four areas: lossless sample preparation, high‐performance chromatography, high sensitivity MS measurements and optimal analysis of the low‐level signals for quantification. A pioneering approach for reducing sample losses is called nanoPOTS (nanodroplet processing in one pot for trace samples) coupled sophisticated protein extraction to dedicated, narrow column chromatography (Zhu *et al*, 2018). SCoPE‐MS employs the tandem mass tag (TMT) isobaric labeling strategy to differentially mass‐label the single cells, to which a ‘carrier channel’ – originally consisting of hundreds of single‐cell equivalents – is added (Budnik *et al*, 2018). Quantification is less straightforward in SCoPE‐MS because their carrier channel also contributes to the reporter ion readout in TMT, and this has been shown to lead to ratio distortions (Cheung *et al*, 2020; Ye *et al*, 2022; Ctortecka *et al*, 2022b). Recently, label‐free approaches have also shown promising results (Saha‐Shah *et al*, 2019; Zhu *et al*, 2019; Cong *et al*, 2021; Matzinger *et al*, 2023).

Our group recently described an ultra‐high sensitivity workflow for single‐cell applications that builds entirely upon readily available components (Brunner *et al*, 2022). Samples are deposited into low adsorption 384‐well plates, allowing parallel preparation with minimal losses, as also used by others (Liang *et al*, 2020; Schoof *et al*, 2021; Specht *et al*, 2021). The resulting peptides are deposited on standard Evotips, from which they are eluted in ‘nano‐packages’ of 20 nl into a preformed gradient. This stored gradient is then separated at a very low flow ‘Whisper’ gradient (100 nl/min) on an analytical column attached to the Evosep system (Bache *et al*, 2018) and electrosprayed into a trapped ion mobility time of flight mass spectrometer (timsTOF) (Meier *et al*, 2018). We performed data acquisition by dia‐PASEF (Meier *et al*, 2020) and achieved median protein identifications of about 1,000 in interphase HeLa cells and up to 2,000 in drug arrested mitotic cells.

Based on these previous experiences with scProteomics, we aimed to develop an improved workflow building on recent developments in multiplex‐DIA (mDIA). Although DIA is overwhelmingly performed in a label‐free and single‐run manner, researchers have long investigated if some of the advantages of multiplexing could be transferred to the DIA setting as well. The principal challenge in mDIA is that multiplexing further multiplies the complexity of already highly complex DIA spectra. To our knowledge, the MEDUSA method first reported this concept (Griffiths *et al*, 2014). Here, ubiquitin and SUMO peptides were differentially labeled with mTRAQ reagents (Δ0, Δ4, and Δ8). Garcia and colleagues combined DIA with SILAC labeling achieving similar identification depth with an order of magnitude better quantitative accuracy than SILAC alone (Pino *et al*, 2021). Very recently, a powerful triplex mTRAQ workflow has been described, which approached similar proteome depth as label‐free DIA data (Derks *et al*, 2022) and exceeded its quantitative precision within runs. Applied to single cells, their ‘plexDIA’ approach reached a depth of 1,000 proteins per cell in an active gradient of 30 min. Apart from these non‐isobaric multiplexing methods, low mass reporter based isobaric methods or precursor coupled reporter tags have also been described (Tian *et al*, 2020; Ctortecka *et al*, 2022a).

Here we present an mDIA workflow that uses dimethyl labeling for multiplexing. This derivatization has been well established in proteomics from the original report (Hsu *et al*, 2003) and extensive subsequent work by the Heck group (Boersema *et al*, 2008, 2009; Taouatas *et al*, 2010). We introduce the principles of dimethyl mDIA, characterize its performance at the bulk proteomics level on modern MS instrumentation and extend to five‐plex at the MS1 and MS2 level by using Lys‐N enzyme. Next, we develop an automated workflow using robotic derivatization combined with Evotips for single‐cell proteomics. We then evaluate the concept of a reference channel in mDIA – in which one of the channels is used as a spike‐in proteome, making measurements universally comparable to each other. In the context of single‐cell proteomics it doubles identifications and throughput. Further, we devise Reference Quantification (RefQuant) an algorithmic strategy to make optimal use of the reference channel for quantification and apply it to single‐cell proteomics. Finally, we combine mDIA with our recently developed spatial technology termed Deep Visual Proteomics (DVP) (Mund *et al*, 2022) to illustrate the potential of mDIA in precision oncology using melanoma as an example.

## Results

### Exploration of dimethyl labeling‐based multiplexed data‐independent acquisition (mDIA)

**Figure 1:**
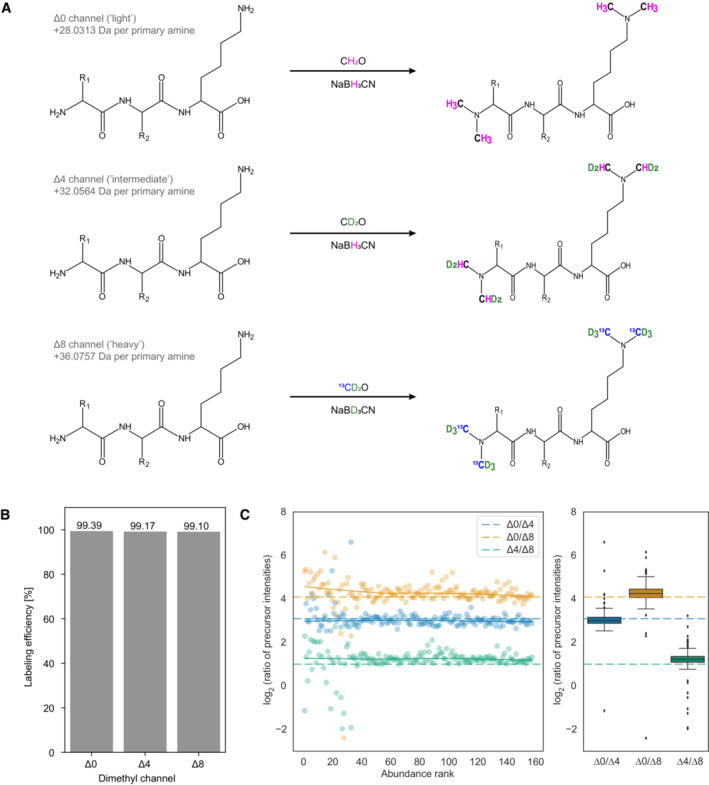
Dimethyl labeling of bovine serum albumin (BSA) combined with multiplexed data‐independent acquisition (mDIA) Stable isotope dimethyl labeling scheme for a three‐plex mDIA setup. Six hydrogens can be replaced by deuterium and the two carbons by ^13^C per dimethyl, of which there are one in tryptic peptides ending in arginine (N‐terminus) and two in those ending in lysine (N‐terminus and N‐ε‐Lys). Depending on the combination of stable isotope labeling reagents, mass tags of 28.0313 Da, 32.0564 Da, or 36.0757 Da are added to each primary amine group of a peptide. A tryptic peptide harboring a C‐terminal lysine residue is depicted.Dimethyl labeling efficiency of peptides derived from intensity ratios of labeled peptides relative to all detected peptides in DDA mode (*n* = 1).Quantification accuracy of dimethyl labeled peptides in DIA mode. Differentially labeled tryptic BSA peptides were mixed in a 17:2:1 ratio of Δ0/Δ4/Δ8 and the data was acquired in DIA mode. Scatterplots (left) illustrate the log_2_ intensity ratios as a function of the peptide abundance rank, which is summarized as a boxplot (right). The box depicts the interquartile range with the central band representing the median value of the dataset. The whiskers represent the furthest datapoint that is within 1.5 times the interquartile range (IQR). In both panels, the expected ratios are marked by colored dashed lines. 50 fmol BSA was injected per replicate (technical replicates, *n* = 3).

Previous approaches to multiplexed data‐independent acquisition (mDIA) mass spectrometry have used SILAC or amino‐reactive labels, specifically the non‐isobaric analog to the iTRAQ reagent, termed mTRAQ (Griffiths *et al*, 2014; Derks *et al*, 2022). To extend the repertoire of available tools for multiplexed analysis of complex proteomes by DIA, we investigated whether a complementary chemical labeling approach based on peptide dimethylation was also suited for this. We decided to explore dimethylation as the derivatization of peptides with dimethyl labels, which is quick, reliable, cost‐efficient, and can easily be automated (Raaijmakers *et al*, 2008). Primary amines occurring on peptide N‐termini or epsilon‐amino groups of lysine residues are thereby derivatized through formaldehyde, to form an intermediate Schiff base that is subsequently reduced by sodium cyanoborohydride, to form a dimethylamino group (Hsu *et al*, 2003). Depending on the combination of stable isotope labeling reagents, this adds a 28.0313 Da, 32.0564 Da, or 36.0757 Da mass tag to each amino group in a given tryptic peptide (referred to as light (Δ0), intermediate (Δ4), and heavy labels (Δ8)), enabling three‐plexed mDIA of proteomes (Fig 1A).

Following a previously established dimethyl labeling protocol (Boersema *et al*, 2009), we first evaluated the feasibility of combining it with mDIA. As a first step, we labeled tryptic peptides derived from bovine serum albumin (BSA) with dimethyl‐Δ0/Δ4/Δ8, which resulted in a labeling efficiency of more than 99% (Fig 1B). To determine quantification accuracy in this setup, we prepared three samples combining the three channels in different ratios (Δ0/Δ4/Δ8 = 17:2:1; 7:2:1 and 5:3:2) and acquired the data in DIA mode (See Materials and Methods), followed by raw data processing with DIA‐NN, a neural network‐based software that recently has been benchmarked for multiplexed DIA‐MS (Demichev *et al*, 2019, 2022; Derks *et al*, 2022). This revealed a high degree of quantification accuracy, even for the highest tested ratio (17:1) (Fig 1C) and as expected deviations from the true ratios were mainly observed for low‐abundant peptides (Appendix Fig S1A and B). We therefore concluded that dimethyl labeling might also be suited for multiplexed acquisitions of complex proteomes in DIA mode.

### mDIA applied to in‐depth quantification of complex proteomes

To determine the suitability of dimethyl labeling for mDIA of whole cell protein extracts, we derivatized tryptic peptides from HeLa cells with three dimethyl mass tags (Δ0, Δ4, and Δ8). Like the labeling of just one protein (Fig 1B), this resulted in an almost complete labeling (> 99%) of all detected peptides for all three channels (Appendix Fig S2A).

Next, we assessed whether identification rates and quantification precision might be compromised by dimethyl labels compared to label‐free analysis. First, we measured triplicates of unlabeled and dimethyl labeled peptides (light channel only, Δ0) and processed the raw data with DIA‐NN using spectral libraries predicted by AlphaPeptDeep (Zeng *et al*, 2022). Those predicted libraries account for potential differences in peptide fragmentation, collisional cross sections, and retention times between unlabeled and dimethyl labeled peptides.

**Figure 2:**
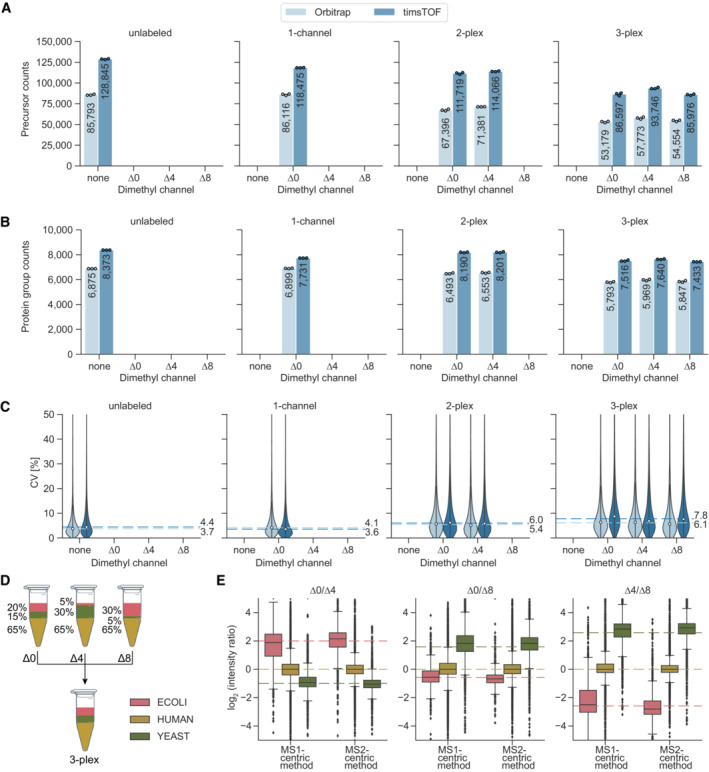
Identification rates, quantification precision and accuracy of dimethyl labeled peptides A, BNumber of quantified HeLa peptide precursors (A) and protein groups (B) for unlabeled, one‐channel (Δ0), two‐plex (Δ0 and Δ4), and three‐plex (Δ0, Δ4, and Δ8) labeled samples. 125 ng of peptides were injected three technical replicates (*n* = 3) per channel.CCoefficients of variation (CV, %) of all protein groups identified per condition for Orbitrap and timsTOF instruments. Protein group intensities were calculated using MaxLFQ‐based protein quantification from ‘Precursor.Normalised’ quantities. Median CVs for three technical replicates (*n* = 3) are shown as dashed lines.DMixing scheme for tryptic peptides of HeLa, *S. cerevisiae*, and *E. coli* at different ratios prior to dimethyl labeling. The three channels were multiplexed in a 1:1:1 ratio and 450 ng total amount (150 ng per channel) was measured in three technical replicates (*n* = 3) on the timsTOF platform using MS1‐ and MS2‐centric methods (Appendix Fig S3A and B).ESide‐by‐side comparison of quantification accuracies between MS1‐centric and MS2‐centric acquisition methods in a mixed species experiment (technical replicates, *n* = 3). Protein group ratios are plotted as boxplots with expected ratios (dashed lines). The box depicts the interquartile range with the central band representing the median value of the dataset. The whiskers represent the furthest datapoint that is within 1.5 times the interquartile range (IQR).

We used a 75 min LC gradient and data acquisition either by regular DIA on an Orbitrap platform, or using a timsTOF HT instrument, with our recently reported optimal dia‐PASEF acquisition schemes (Appendix Fig S3A, Materials and Methods) (Skowronek *et al*, 2022b). Whereas the timsTOF platform generally yielded higher identification numbers and greater quantification precision, the number of quantified precursors and protein groups between unlabeled and one‐channel dimethyl labeled samples was similar on both instruments. This demonstrates that derivatization of peptides with dimethyl groups does not negatively impact peptide identification rates (Fig 2A–C). Using the Orbitrap instrument, we quantified about 7,000 protein groups (85,000 precursors) from 125 ng of injected peptides in both unlabeled and dimethyl‐∆0‐labeled samples, with a median CV of 4.8%. In contrast, the timsTOF yielded about 20% more protein groups and 50% more precursors in unlabeled samples (about 8,400 protein groups and 128,000 precursors) and 11% more protein groups and 40% more precursors (7,700 protein groups and 118,000 precursors) in labeled samples with the same injection amount (Fig 2A and B).

**Figure EV1:**
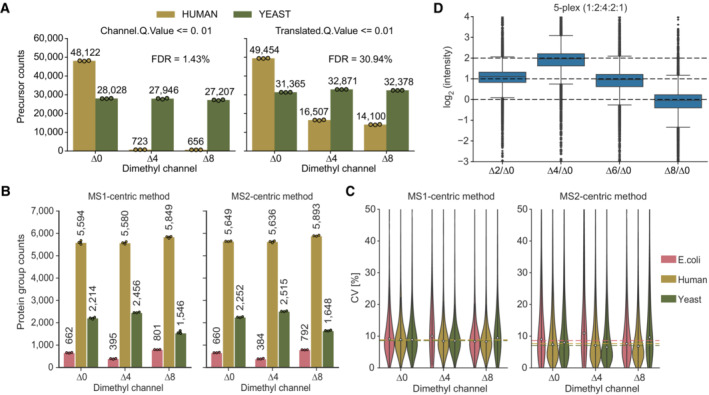
Identification rates and coefficient of variation (CVs) in the mixed species experiment Determination of the empirical FDR based on filter cut‐offs of ‘Channel.Q.Value’ ≤ 0.01 (left panel) and ‘Translated.Q.Value’ ≤ 0.01 (right panel). All channels contain 100 ng of labeled tryptic yeast peptides. Additionally, channel ∆0 contains 100 ng labeled tryptic HeLa peptides (human) (technical replicates, *n* = 3).Tryptic peptides from E. coli, yeast and human were combined at defined ratios before dimethyl labeling. Side‐by‐side comparison of the number of quantified protein groups for the individual species with MS1‐ and the MS2‐centric methods. 100 ng of peptides were injected per channel (technical replicates, *n* = 3).Coefficients of variation (CVs, %) of protein groups shown in (A). Median CVs for three technical replicates (*n* = 3) are shown as dashed lines.Quantification accuracy assessment of 5‐plex by Lys‐N of labeled HeLa peptides in the respective channel (∆0, ∆2, ∆4, ∆6, ∆8) in a ratio of 1:2:4:2:1 (technical replicates, *n* = 3). Protein group ratios are plotted as boxplots with expected ratios as dashed lines normalized to the ∆0 channel. The box shows the interquartile range with the central band representing the median value of the dataset. The whiskers represent the furthest datapoint within 1.5 times the interquartile range (IQR).

When increasing proteome complexity by adding a second channel (Δ4), identifications remained almost constant at both the precursor and the protein level on the timsTOF platform (about 115,000 precursors and 8,200 protein groups), whereas we observed a slight decrease on the Orbitrap instrument (about 20% for precursors and 4% for proteins groups). In a three‐plex mDIA setup, protein group identifications further decreased on the Orbitrap and slightly decreased with the timsTOF (Fig 2B), indicating that the deconvolution of multiplexed spectra is challenging and that the ion mobility dimension of the timsTOF platform helps to resolve this complexity. Quantitative reproducibility measured by coefficient of variation (CV) decreased only slightly from single to triple labeling (median 4.1% at a single channel and 7.8% at three channels) (Fig 2C). We empirically determined the false discovery rate (FDR) in a two‐species experiment (Fig EV1A). For bulk and equally abundant channels, the FDR was 1.5% at a ‘Channel.Q.Value’ cutoff of 0.01 (see Fig EV1A for comparison of ‘Channel.Q.Value’ and ‘Translated.Q.Value’). These results indicate absent or minimal cross‐talk between the dimethyl channels.

To investigate quantification accuracy in a three‐plex setup we combined tryptic peptides from human (HeLa), *S. cerevisiae*, and *E. coli* at defined ratios for three labels (Fig 2D). This mixing scheme creates a benchmark of known protein ratios, which can be compared with the ratios of measured intensity values. In addition to our initially designed dia‐PASEF method, we generated an alternative method, consisting of multiple MS1 scans in between dia‐PASEF scans of each duty cycle (Appendix Fig S3B) similar to what was performed previously on Orbitrap instruments (Xuan *et al*, 2020; Derks *et al*, 2022). We used this MS1‐centric method for direct comparison with our standard MS2‐centric method, by quantifying protein ratios across channels in the three‐plex, mixed species experiment (See Materials and Methods). This revealed that with both methods the measured protein group ratios largely agreed with the expected ones and that the MS2‐centric method slightly outperformed the MS1‐centric method in terms of accuracy, with similar identification rates and quantification precision (Figs 2E and EV1B and C).

### Extending the scope of multiplexing by dimethyl labels

Although trypsin is the most frequently used protease for MS‐based proteomics applications, in the context of dimethyl labeling it limits multiplexing to only three channels if a mass difference between channels of 4 Da is desired at MS1 and MS2 level. At the MS1 level alone, Lys‐C in combination with different isotopes of formaldehyde and cyanoborohydride enables five‐plex dimethyl labeling (Wu *et al*, 2014).

**Figure 3:**
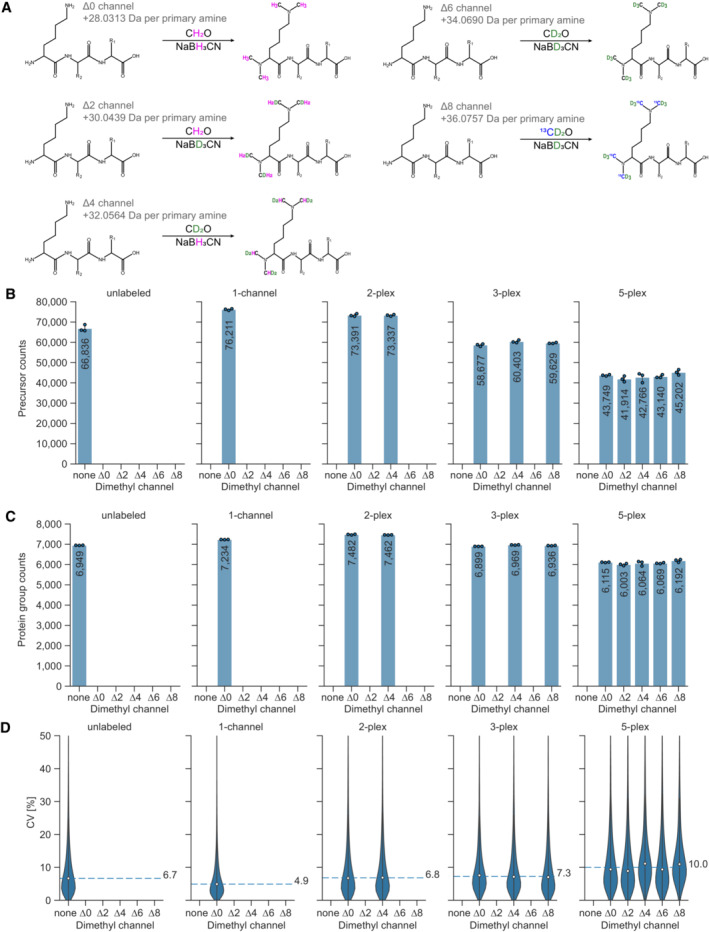
Five‐plex dimethyl labeling using Lys‐N protease and mDIA AStable isotope dimethyl labeling scheme for a five‐plex mDIA experimental setup with Lys‐N digested peptides. Depending on the combination of stable isotope labeling reagents, mass tags of 28.0313 Da, 30.0439 Da, 32.0564 Da, 34.0690 Da, or 36.0757 Da are added to each primary amine group on a peptide. Since Lys‐N hydrolyses the N‐terminal side of lysine residues, two labels are clustered on the N‐termini of peptides (N‐terminus and N‐ε‐Lys).B, CNumber of quantified HeLa peptide precursors (B) and protein groups (C) for unlabeled (none), one‐channel (Δ0), two‐plex (Δ0 and Δ4), three‐plex (Δ0, Δ4, and Δ8), and five‐plex (Δ0, Δ2, Δ4, Δ6, and Δ8) labeled samples. 100 ng of peptides were injected per channel (technical replicates, *n* = 3).DCoefficients of variation (CV, %) of all protein groups identified per condition in the dimethyl five‐plex setup, using Lys‐N as protease. Protein group intensities are calculated using MaxLFQ‐based protein quantification from ‘Precursor.Normalised’ quantities. Median CVs for three technical replicates (*n* = 3) are shown as dashed lines.

Lys‐C derived peptides harbor two primary amino groups for labeling – one of them is located on the N‐terminus and the second one on the side chain of the C‐terminal lysine residue. Digesting proteins with Lys‐N instead generates a N‐terminal lysine on each peptide, and thus two primary N‐terminal amines for labeling (Raijmakers *et al*, 2010). Five‐plex labeling of Lys‐C or Lys‐N‐derived HeLa peptides thus results in isotopologues that are separated by 4 Da from each other, which would be sufficient for accurate quantification (Fig 3A).

Lys‐N‐derived and dimethylated peptides form more b‐ions during MS2 fragmentation than tryptic peptides (Taouatas *et al*, 2008) and the two dimethyl labels are carried by b‐ions. We reasoned that this should enable MS2‐based quantification in five‐plex experiments. In contrast, fragmented five‐plex labeled peptides derived from Lys‐C carry one dimethyl label on each b‐ and y‐ion. Each of them separates from its isotopologues by only 2 Da on MS2 level, which leads to major overlaps of isotope clusters.

We therefore explored whether Lys‐N‐mediated digestion combined with five‐plex dimethyl labeling could be a straightforward way of further increasing multiplexing for mDIA. We used HeLa lysates and digested them with Lys‐N, followed by derivatization with five‐plex dimethyl mass tags, achieving more than 97% labeling efficiency (Appendix Fig S2B). After combining the labeled peptides in equal ratios, we quantified them using the timsTOF instrument, with dia‐PASEF as scan mode and employing methods optimized for Lys‐N digestion with py_diAID (Appendix Fig S3C) (Skowronek *et al*, 2022b). This led to the quantification of about 7,000 protein groups in both unlabeled and labeled samples with just one‐channel (Δ0) (Fig 3B and C). In contrast to dimethyl labeled tryptic peptides, combining up to three labels did not significantly decrease protein group identifications and even in the five‐plex setup, protein identifications decreased by only about 15% compared to one‐channel dimethyl measurements. Similar to the three‐plex mDIA result described above, CV values did not change until 3‐plex, while they increased slightly in 5‐plex (Fig 3D). The median fold changes of a mixing experiment largely agreed with their expected values in 5‐plex (Fig EV1D). This demonstrates the feasibility of multiplexing up to five samples in mDIA experiments using dimethyl labeling.

### Robust and automated ultra‐high sensitivity mDIA workflow with a reference channel

**Figure 4:**
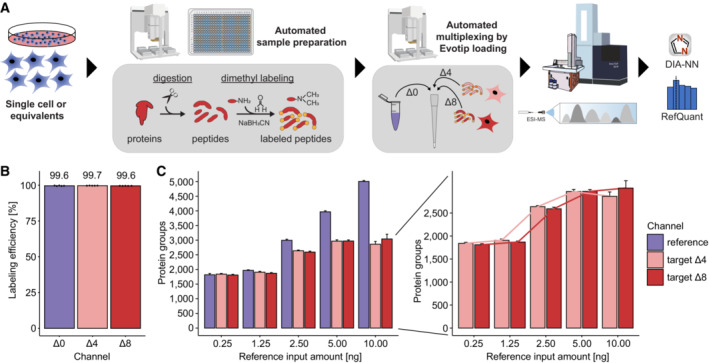
Streamlined and automated ultra‐high sensitivity mDIA workflow Single HeLa cells or single‐cell equivalents are processed in a standard 384‐well plate on a Bravo robot (Agilent) by lysis in TEAB and ACN, tryptic digestion, dimethyl labeling, and multiplexing by loading the different mDIA channels onto the same Evotip. LC–MS analysis is done by an Evosep One with low‐flow chromatography coupled to a timsTOF SCP instrument. The data is analyzed by DIA‐NN, followed by our algorithm RefQuant (see main text).1 ng tryptic peptides from HeLa cells were labeled with dimethyl mass tags Δ0, Δ4, and Δ8 and acquired individually in DDA mode to determine labeling efficiency. Efficiency is calculated based on intensity ratios of labeled peptides relative to all detected peptides. The labeling efficiencies were consistently higher than 99.5% for all channels in quintuplicate measurements (technical replicates, *n* = 5).Effect of varying the protein input in the reference channel for protein identification across all channels. Increasing the total protein amount in the reference channel linearly increases protein identifications in the reference channel (left), but importantly protein identifications reach a plateau in the target channels (Δ4 and Δ8) with single‐cell equivalents upon 5–10 ng in the reference channel (scReference dataset). Connected lines between increasing reference input amounts show a sigmoidal relation (right). Error bars represent the standard deviation of quintuplicate measurements (technical replicates, *n* = 5).

Having established the dimethyl mDIA derivatization for bulk proteomics samples, we set out to combine it with our recently published ultra‐high sensitivity single‐cell proteomics workflow (Brunner *et al*, 2022). We used a 384‐well plate format for all ultra‐high sensitivity experiments to reduce reaction volumes. Furthermore, we adapted the lysis and digestion buffer to an amine‐free buffer using triethylammonium bicarbonate (TEAB) to enable the amine directed chemistry of dimethyl labeling (Fig 4A). The dimethylation reaction itself is performed by simple addition of the chemicals to the peptide mixtures in small volumes achieving a labeling efficiency greater than 99.5% for all channels at 1 ng tryptic HeLa peptides (Fig 4B). Subsequent cleanup of the derivatization reaction has previously been done in an in‐column format (Boersema *et al*, 2009), but such a step is generally omitted in single‐cell applications to avoid sample loss. In our format, however, the cleanup and combination of the separately labeled single cells can easily be performed in the Evotips. Each of the three in‐solution labeled digests in a 384‐well plate are sequentially deposited onto the same Evotip, thus, the derivatization reaction does not complicate the workflow and is completely transparent to the user. The entire sample preparation, including the combination of the labeled channel while loading of the Evotips, was automated on a standard Bravo robot (Agilent) to streamline the workflow, enhance reproducibility and automate for increasing throughput (Fig 4A, Materials and Methods). This also adds traceability and enables compliance and quality control.

In the Evosep instrument, peptides are eluted from the tips in nano‐packages and analyzed at a very low ‘Whisper’ flow (100 nl/min) with a gradient of 31 min and overhead time to the next injection of 7 min (‘40 samples per day method’). With such a low flow rate, it is important to avoid any post‐column dead volumes. We therefore sought to eliminate the post‐column connectors that we had employed previously by using pulled columns packed into the electrospray tip (IonOpticks) (preprint: Sandow *et al*, 2021). This setup makes the workflow very robust and reproducible, and also improved chromatographic resolution, with peaks eluting in full width half maximum (FWHM) with a median of about 2.7 s, corresponding to an elution volume of only 8 nl at peak width.

Finally, we analyzed the multiplexed single‐cell samples using an optimal dia‐PASEF method (Skowronek *et al*, 2022b) with eight dia‐PASEF scans and a mass range of 300 to 1,200 and ion mobility of 0.7 to 1.3 on the timsTOF SCP (Appendix Fig S3D, See Materials and Methods).

In the bulk experiments above, we had established that the different non‐isobaric channels in the mDIA workflow are decoupled from each other in terms of quantification. More importantly, different precursors that are fragmented together still do not contribute to the same ‘reporter ions’ as they may do in TMT labeling. Thus, the only interference in terms of quantification has to come from chemical noise of different precursors that happen to share a fragment within the MS2 resolution of the mass spectrometer. We therefore reasoned that we could use one of the mDIA channels as a ‘reference channel’ comprised of the same input material across all samples. Apart from providing a common standard for identification and quantification, such a reference channel conceptually decouples identification from quantification. This is because the reference channel proteome is easily identified due to its higher signal intensity and its uniformity across samples. Subsequently, the software needs to transfer the quantification boundaries from the reference channel to the target channels containing the single‐cell proteomes.

**Figure EV2:**
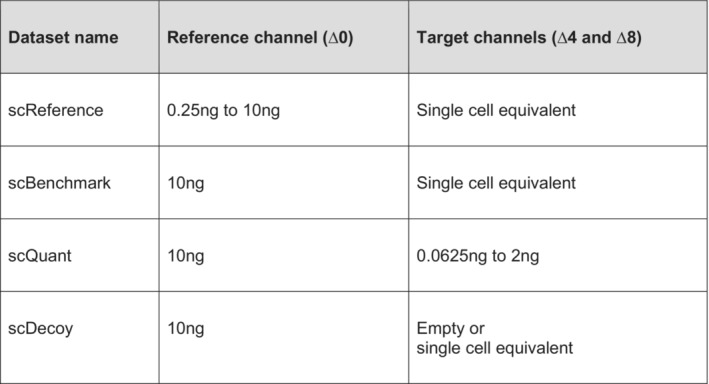
Overview of datasets used for evaluation of the reference channel

**Figure EV3:**
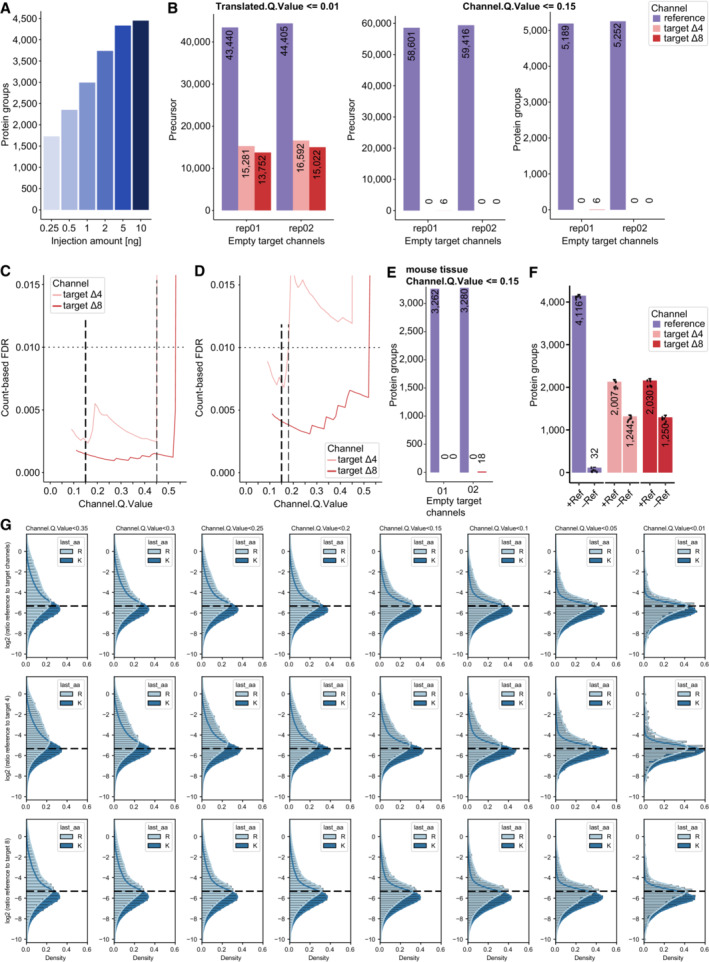
Evaluation of ‘Translated.Q.Value’ and ‘Channel.Q.Value’ on identifications in empty target channels and quantification in single‐cell equivalents ADilution series of HeLa peptides to define protein identifications in mDIA workflow, in which the linear increase with input amount levels off at about 10 ng (technical replicates, *n* = 3).BPrecursor identifications of empty target channels at 1% ‘Translated.Q.Value’ revealed more than 1% false positives (left). Precursor (middle) and protein (right) identifications of empty target channels at 15% Channel.Q.Value showed a FDR lower than 1% (scDecoy dataset).C, DCount‐based FDR estimation on precursor (C) and protein level (D) by dividing two quantities, namely the maximum number of identified precursors or proteins in four empty target channel runs by the minimum number of identified precursors or proteins in a total of four target channels (target channel ∆4 and ∆8) which contain single‐cell equivalent amounts (scDecoy). The count‐based FDR reveals a ‘Channel.Q.Value’ filter of 0.45 for precursors and 0.17 (dashed line) for proteins at 1%. For comparison, the used ‘Channel.Q.Value’ cutoff of 0.15 is shown as well (thick dashed line).EPrecursor count‐based FDR derived from empty target channels with mouse liver tissue samples at 15% Channel.Q.Value is much below 1%.FImpact of the reference channel on identification in the target channels of single‐cell equivalents. We compare single‐cell equivalents in the target channels with (10 ng ∆0‐labeled HeLa) and without reference channel (10 ng unlabeled HeLa) (technical replicates, *n* = 6). Bars and errors depict median ± standard deviation.GQuantification evaluation of ‘Channel.Q.Value’ filter. Ratios of reference to target channels (top), channel 4 (middle) and 8 (bottom) by RefQuant (Fig 5) are shown for arginine and lysine precursor at a given filter of ‘Channel.Q.Value’ (technical replicates, *n* = 5). At all filtering steps, the modes of the distributions are close to the expected ratio (dashed line) with a systematic skew towards lower ratios, which is more pronounced for the arginine precursors. The skews in the distributions are decreased with increased filtering stringency in a gradual manner. The mean quantification ratios are as expected, whereas they start to deviate at 0.2.

To investigate the reference channel concept in mDIA, we first systematically increased its loading in the ∆0 channel (The specific datasets used for evaluation are summarized in Fig EV2). With our current chromatographic set‐up, the number of identifications leveled off at roughly 10 ng, which we therefore chose as the reference channel amount for all subsequent experiments (Fig EV3A).

Next, we kept the reference amount constant, but varied the amount of peptide in the target channels to explore how much the reference channels would support identifications of weak signals. The DIA‐NN software that we used for analysis does not directly have a notion of a reference channel, but considers the boundaries of the channel with the highest scoring precursor as internal reference for transfer. To assess identification confidence, DIA‐NN reports a ‘Translated.Q.Value’ and a ‘Channel.Q.Value’ parameter (Fig EV3B). As noted above for bulk proteomics experiments, the former is unsuitable for FDR determination. Therefore, we experimentally determined the value of the ‘Channel.Q.Value’ at which features from the reference channel could safely be transferred into the target channels. To this end, we measured mDIA samples in which we left one or both of the target channels empty (scDecoy). This revealed that a ‘Channel.Q.Value’ of 0.45 led to a count‐based FDR of 1% for precursors and 0.17 for protein groups (Fig EV3C and D, Materials and Methods). At the latter value, we also obtained accurate quantitative ratios between the target and reference channel as judged by defined mixing experiments (Fig EV3G). To ensure high confidence identifications in the target channels with single‐cell equivalents and good quantification, we henceforth filtered the data with a ‘Channel.Q.Value’ of 0.15 as well as the recommended parameters of DIA‐NN. Note that this same empirically determined cutoff was also supported by experiments on mouse liver tissue (Fig EV3E).

With these parameters, we investigated whether increasing amounts in the reference channel might influence the number of proteins found in single‐cell equivalents in the target channels (scReference dataset, amounts from 0.25 ng to 10 ng in the reference channel and single‐cell equivalents in the target channels). Importantly, while increasing the reference channel input amount beyond 5 ng, the target channel identifications remained the same (Fig 4C). We conclude that these channels are isolated from each other as expected from the mDIA concept. Additionally, to show that this identification increase is due to the reference channel and not simply because of adsorptive losses during sample analysis, we compared the identifications in the target channels with reference channel and without, while spiking 10 ng of unlabeled HeLa instead. Identifications from single‐cell equivalents adding an unrelated 10 ng proteome were 1,247 proteins, while using a 10 ng reference channel were 2,018 protein groups (mean of five replicates in each target channel). Thus, the increase in identifications is overwhelmingly due to the reference channel, rather than simply an effect of adsorptive losses (Fig EV3F).

### RefQuant improves quantification by sampling ratios relative to the reference channel

**Figure 5:**
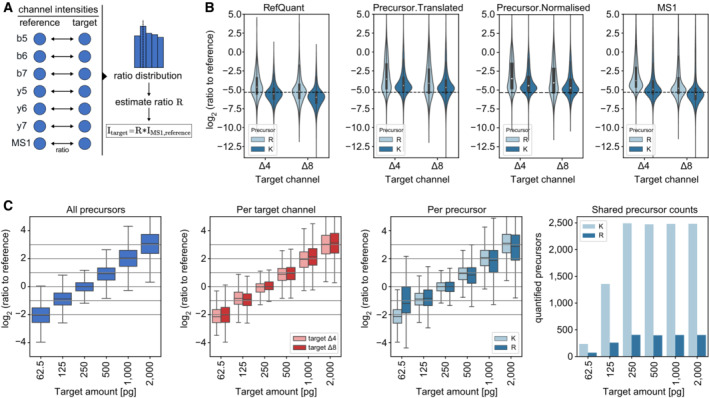
RefQuant quantifies single‐cell equivalents accurately based on sampling ratios relative to the reference channel Concept of RefQuant in calculating ratios between each fragment and MS1 peak for each precursor between reference and each target channel. The resulting ratio distribution is filtered for the first 40% of the quantile and the ratio factor R is calculated by the mean of the resulting distribution. This ratio factor R is then multiplied by the reference intensity to retrieve the RefQuant target intensity.Ratio of reference channel to each target channel by arginine (R) and lysine (K) precursor based on quantities from RefQuant (left), ‘Precursor.Translated’ and ‘Precursor.Normalised’ by DIA‐NN (middle) and MS1 by DIA‐NN (right) on the scBenchmark dataset (technical replicates, *n* = 5). RefQuant showed best the expected ratios in both target channels compared to DIA‐NN quantities. The violin plot shows the distribution of the data while the box depicts the interquartile range with the central band representing the median value of the dataset. The whiskers represent the furthest datapoint within 1.5 times the interquartile range (IQR).RefQuant accurately quantified four‐fold differences in protein amount between reference and target channels (scQuant dataset, 62.5 to 2000 pg in target channel, 10 ng in reference channel) and correctly extracted the expected ratios across channels for lysine (K) and arginine (R) precursors (technical replicates, *n* = 5). The ratio of reference to target channel was calculated using RefQuant. All log_2_ ratios were normalized to the single‐cell equivalent sample in the target channel (250 pg) and only shared precursors are used (right panel). The box depicts the interquartile range with the central band representing the median value of the dataset. The whiskers represent the furthest datapoint within 1.5 times the interquartile range (IQR). Source data are available online for this figure.

**Figure EV4:**
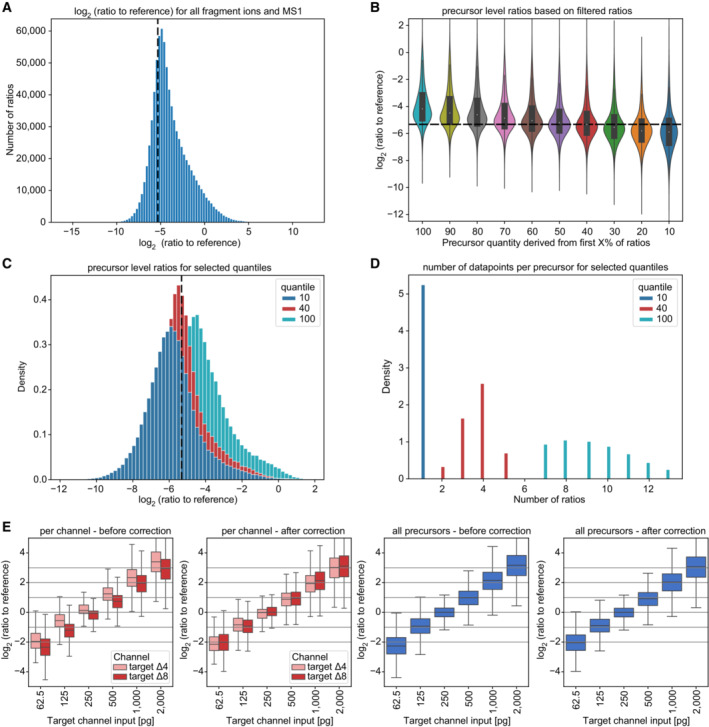
Ratios to reference channel evaluation and channel correction comparison Ratios to the reference for all available ions (fragment ions and MS1 peaks, scBenchmark dataset), revealing a distribution that has a mode proximate to the expected ground truth (dashed line). The distribution is asymmetric with a skew towards less extreme ratios. A potential explanation for this skew is that noise or interferences are dominant for a fraction of the ratios (a ratio of 0 might be a ‘noise vs. noise’ comparison).An approach to mitigate this asymmetry is to filter out some ions before estimating the precursor level ratio. For this, the ratios of a precursor are sorted ascending and only the first ratios are retained (up to the ‘X value’ indicated) using the scBenchmark dataset. The precursor ratio is estimated by taking the mean of the remaining ratios. We see that retaining 40% of the ratios matches the ground truth well and is more symmetric (technical replicates, *n* = 5). The violin plot shows the distribution of the data while the box depicts the interquartile range with the central band representing the median value of the dataset. The whiskers represent the furthest datapoint within 1.5 times the interquartile range (IQR).A more detailed comparison of the distributions when taking 10%, or 40% of ratios as compared to all ratios.Number of available ratios per precursor after filtering. We see that in general between 7 and 14 ratios are available, which reduces to 2–5 when taking the 40% quantile.Channel correction comparison in scQuant dataset in comparison of all precursor and target channel (technical replicates, *n* = 5). Ratio of reference channel to target channel was calculated using RefQuant. All log_2_ ratios were normalized to the single‐cell equivalent sample in the target channel (250 pg). A basic median normalization between the channels was applied. The violin plot shows the distribution of the data while the box depicts the interquartile range with the central band representing the median value of the dataset. The whiskers represent the furthest datapoint within 1.5 times the interquartile range (IQR).

Having shown the advantages of a reference channel to substantially increase protein identifications in the target channels (Fig 4C) and the ‘Channel.Q.Value’ cutoff of 0.15 to filter out false identifications (Fig EV3B, right panel), we investigated if its benefits also extend to quantification. We reasoned that the standardized reference proteome present at higher amounts might directly improve quantification in the target channels by effectively reducing technical variation across runs – an idea already implemented in the super‐SILAC strategy (Geiger *et al*, 2010). To enable this analysis, we implemented an approach that we term **Ref**erence **Quant**ification (RefQuant), which is based on sampling all available ratios relative to the reference channel (Fig 5, Materials and Methods). In short, the ratios of individual fragment ions as well as MS1 peaks between reference and target channels are extracted, which results in a distribution of ratios (Figs 5A and EV4A). Subsequently, a best ratio R is estimated from the ratio distribution in a robust manner by taking the mean from the 40% lower quantile of ratios. We determined this threshold empirically using the scBenchmark dataset (Figs EV2 and EV4B). The intensity of the target channel is then calculated by multiplying the ratio R with a scaling factor representing the intensity of the reference channel. This gives a re‐scaled precursor intensity value. The scaling factor is derived per precursors as the median reference intensity over all runs, in order to further stabilize the estimate. An interesting property of RefQuant is that it utilizes the high relative ratio to the reference channel in two ways: (i) the prior knowledge about the high expected ratio is used as an effective ‘noise filter’ (Fig EV4A–D) and (ii) the higher abundance of the reference channel stabilizes quantification, which should conceptually reduce overall noise (Appendix Text S1).

To test RefQuant, we initially applied it to a single‐cell equivalent benchmark dataset (scBenchmark, target channels with 250 pg HeLa peptides, reference channel with 10 ng) and one in which we varied the amount of HeLa peptides in each of the target channels covering four‐fold expression differences in both directions from a single‐cell equivalent (scQuant, target channels from 62.5 to 2,000 pg HeLa peptides, reference channel with 10 ng; Fig EV2).

In the scBenchmark dataset, the ratio between the reference and target channel is well reconstructed by RefQuant, as compared to the standard method of only extracting the target ion intensities from DIA‐NN without using the reference channel (Fig 5B). This indicates good reproducibility and also good quantitative accuracy. This was the case both for MS1 (‘Ms1.Area’) and MS2 (‘Precursor.Translated’ and ‘Precursor.Normalised’) quantification. In particular, quantification without RefQuant had more scatter and RefQuant best reconstructed the true ratios. DIA‐NN reports a difference between quantification of tryptic peptides with C‐terminal lysine (‘lysine precursors’) and arginine (‘arginine precursors’). RefQuant also accurately quantifies arginine precursors, which are particularly challenging to quantify, presumably because they have shared y‐ions between mDIA channels. Next, we specifically investigated the effects of varying the peptide amount in the reference channel with or without RefQuant. We analyzed the ratios to reference over a range from single‐cell equivalents (0.25 ng) to 10 ng in the reference channel (Appendix Fig S4A–E). Without RefQuant, we observed a clear dependence of the distribution spread (measured via the standard deviation) on the amount of reference channel at the MS1 level. This spread was substantially reduced on the MS2 level (assessed via ‘Precursor.Translated’ and the ‘Precursor.Normalised’ quantities from DIA‐NN). However, when applying RefQuant to the same data virtually no dependence of the standard deviation on reference channel amount was observed. Additionally, only RefQuant eliminated systematic ratio compression as long as the reference channel was dominant. This demonstrates that RefQuant specifically utilizes the information of the reference channel and thereby removes potential artifacts.

We next turned to assess the accuracy of different fold changes in single‐cell equivalents, the most important outcome of single‐cell proteomics. The scQuant dataset shows that RefQuant on mDIA accurately retrieves four‐fold differences in the target channel with either decreasing or increasing amount in the target channel. There were no differences in the two target channels (∆4 and ∆8) after median correction (Figs 5C and EV4E). The quantification of lysine peptides, which have no shared fragments between channels, has less variation compared to arginine precursors although the results of the arginine peptides may be skewed by their small number at 62.5 pg (Fig 5C). Finally, we investigated the quantitative reproducibility of single‐cell equivalents in dependence on the reference channel amount (scReference). The Pearson correlations were always greater than 0.88 using RefQuant or 0.8 for MS1 quantities and did not differ between target channels ∆4 and ∆8 (Appendix Fig S4B and C).

### mDIA doubles protein identifications per single cells

Having shown the benefits of the reference channel for identification and quantification, we next applied our mDIA workflow to single cells. We designated one of the channels as the reference (∆0), leaving two target channels (∆4 and ∆8) for single‐cell analysis. With this setup, we routinely analyzed 80 single cells per day.

**Figure 6:**
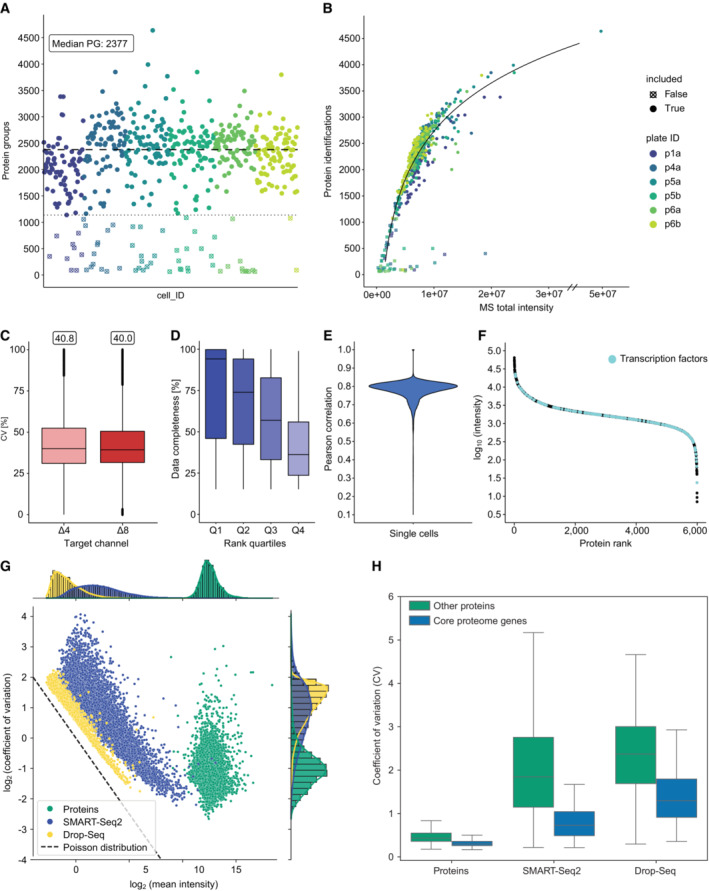
Reference channel doubles protein identifications per single cell and the concept of a stable proteome is still valid Protein identifications of single‐cell measurements. Four hundred and seventy‐six single HeLa cells reveal a median identification of 2,377 with few single cells of identifications up to 4,000 protein groups over six 384‐well plates (disregarding a single outlier of 4,600 protein groups).Protein identifications versus the total sum of MS signal intensity per cell measurement shows a logarithmic dependency.CV distribution of single cells in each target channel (biological replicates, *n* = 476). The box depicts the interquartile range with the central band representing the median value of the dataset. The whiskers represent the furthest datapoint within 1.5 times the interquartile range (IQR).Completeness of ranked quartiles (Q1: 1–924; Q2: 925–1,848; Q3: 1,849–2,772; Q4: 2,773–3,696). Proteins with high rank show higher data completeness compared to low ranked proteins (biological replicates, *n* = 476). The box depicts the interquartile range with the central band representing the median value of the dataset. The whiskers represent the furthest datapoint within 1.5 times the interquartile range (IQR).Pearson correlation of single cells of the mDIA workflow (median 0.79).Rank plot of close to 6,000 proteins. Transcription factors are highlighted across abundance range (cyan).Coefficients of variation of single‐cell mDIA protein expression plotted against the mean intensity of each protein for single‐cell proteins (green) and scRNAseq of SMART‐Seq2 (blue) and Drop‐Seq (yellow).Comparison of coefficients of variation between single‐cell proteomics and scRNAseq with regards to the ‘core proteome’ (blue) and other genes (green) (biological replicates, scProteomics *n* = 476, SMART‐Seq2 *n* = 720, Drop‐seq *n* = 5,665). The box depicts the interquartile range with the central band representing the median value of the dataset. The whiskers represent the furthest datapoint within 1.5 times the interquartile range (IQR). Source data are available online for this figure.

The only difference to the ultra‐high sensitive mDIA workflow described above is that we upfront sorted single HeLa cells from culture by flow cytometry into 384‐well plates. We FACS‐isolated the cells in an unbiased manner; thus we expected a range of cell sizes depending on the cell cycle stage. This was reflected in a wide range of protein identifications across analyzed cells (Fig 6A). To investigate the cause of this spread, we considered the summed MS signal of all peptides as a proxy for total protein amount in our single cells. Plotting this signal against protein identifications revealed a strong dependency of input amount on proteome depth (Fig 6B), but is not caused by doublets (Appendix Fig S5A). This suggests that further gains in ion signals will allow even deeper coverage in the reference mDIA workflow in the future. We also note that FACS‐sorting may lead to some drop outs which might be overcome with more sensitive sorting strategies as well as the possibility to take pictures of each isolated single cell to validate an intact cell for analysis as done in the cellenONE instrument (preprint: Hartlmayr *et al*, 2021). This may help to reduce drop outs and throughput.

However, even at this stage, we already identified 2,377 protein groups and 7,607 peptides per single cell and reached almost 4,000 protein groups in a few single cells (disregarding a single outlier at 4,600 identifications) (Fig 6A, Appendix Fig S5B and C). Across our 476 cells in the entire dataset, we obtained 5,997 proteins, a substantial part of the proteome expressed by a single cell. Even compared to some of the deepest proteomes that were achieved in this cell type with hundreds of μg rather than pg input (Bekker‐Jensen *et al*, 2017), our single‐cell proteomes together account for half of the proteins.

This is more than twice the number of the median identified in interphase cells in our previous publication, almost entirely attributable to the improvement due to the reference channel (Fig 6A) (Brunner *et al*, 2022). The overall coefficient of variation due to technical and biological factors was 0.4 without bias between the target channels (Fig 6C). Overall data completeness was high. After we filtered out proteins that were only detected in 15% of the cells – possibly due to biological variation – data completeness of the remaining 3,696 proteins was 59.3% across the entire dataset. As expected, this depended on the abundance of the proteins, with completeness of the top rank order quartile at 94.1% and the lowest quartile at 36.2% (Fig 6D). The median overall Pearson correlation of the single cells was 0.79 (Fig 6E).

Overall, our protein signals cover more than four orders of magnitude of dynamic range, in which we identify many transcription factors, like the Hox family, STAT1‐3 and SOX6, which range over the whole abundance range, but are mainly expressed at low amounts (Fig 6F). This constitutes to 20% of all human transcription factors (Lambert *et al*, 2018).

### Stable proteome at deeper proteome coverage

In our recent single‐cell publication, we discovered evidence for a stable proteome while the transcriptome had a much higher overall variability as measured by CV. We attributed this difference to the fact that single cells need a complete proteome to function, whereas transcripts for genes expressed in these same cells are only needed rarely (Brunner *et al*, 2022). Thus, numbers of transcripts for many expressed genes are often below one on average across many single cells. A limitation of our previous study was the coverage of the single‐cell proteome – about 1,000 proteins in interphase cells and up to 2,000 proteins in drug arrested mitotic cells.

Here, we used the same strategy as in our label‐free single‐cell dataset (Brunner *et al*, 2022). In brief, we investigated the variability of protein expression by the CV as a function of abundance by MS intensity (See Materials and Methods). Remarkably, we still see relatively small CVs across covered abundances for the whole measured dynamic range (Appendix Fig S5D). Previously, we had defined a ‘core proteome’ of the top 200 proteins with at least 70% data completeness. We also observe lower CVs and higher mean MS intensity for these compared to noncore proteins (Appendix Fig S5E and F). The somewhat increased CVs of the proteins that were only quantified here, is readily explained by their lower signal in the MS.

As before, we next compared our mDIA single‐cell dataset to scRNAseq data based on Drop‐Seq (Macosko *et al*, 2015) and SMART‐Seq2 (Picelli *et al*, 2014) on the same cell type. In the count distribution plot, scRNAseq and mDIA scProteomics data again separate from each other, indicating the stable proteome at higher proteomic coverage (Fig 6G). CV values for the proteins are tight and low while scRNAseq has higher CV values (Fig 6H). Together these data show that the concept of a stable proteome still holds true at the higher proteomic depth achieved here.

### mDIA advances single cell type resolved spatial proteomics in oncology

**Figure 7:**
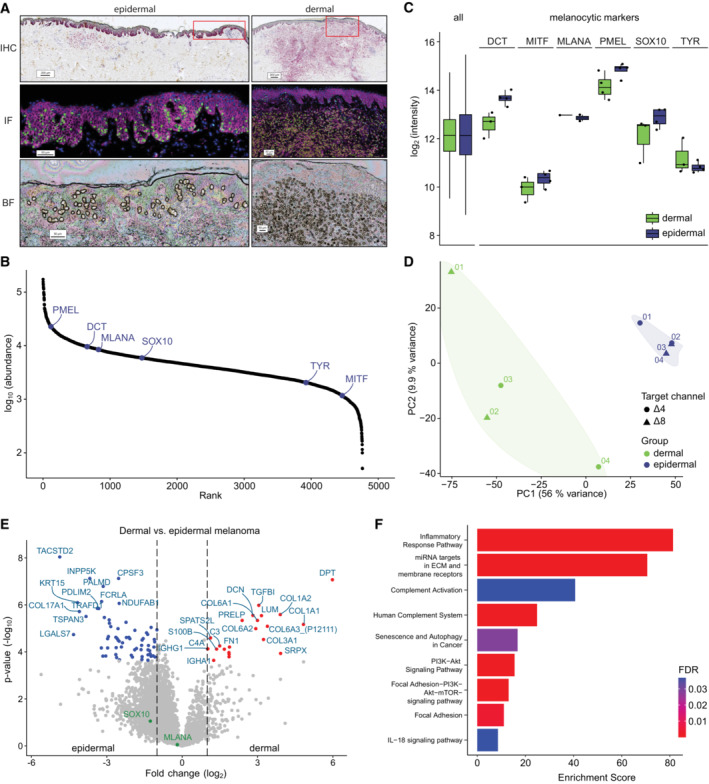
mDIA‐DVP applied to primary cutaneous melanoma showcases its potential for precision oncology Macroscopic overview of primary cutaneous melanoma (type SSM, Breslow 3.5 mm) including Melan‐A immunohistochemistry (IHC, pink) and CD44/Sox10 immunofluorescence (IF, pink/green), as well as a brightfield (BF) image after laser microdissection. Segmented melanoma cells are highlighted (yellow outlines). Quadruplicates of 100 shapes (20 cell equivalents) were laser‐microdissected from the epidermal (left) and dermal (right) compartment using Deep Visual Proteomics (DVP).Rank plot of all proteins identified in melanoma cells. Six out of seven melanocyte identity markers were identified (blue).Boxplot of log_2_ intensities of all proteins compared to six out of seven identified melanocytic markers (Belote *et al*, 2021) (biological replicate, *n* = 4 in each group). The box depicts the interquartile range with the central band representing the median value of the dataset. The whiskers represent the furthest datapoint within 1.5 times the interquartile range (IQR).Principal component analysis of dermal and epidermal melanoma cells which differentiate on PC1 irrelevant of the target channel used.Differential protein expression between epidermal (left) and dermal (right) melanoma cells. Significant proteins (Benjamini–Hochberg corrected multiple‐sample *t*‐test, *q*‐val/FDR < 0.01) with a log_2_ fold change > 1 (red) and < 1 (blue) are highlighed, respectively.Overrepresentation analysis of significantly enriched proteins in dermal melanoma using the Wikipathway database. Color represents FDR (threshold < 0.05).

We reasoned that the multiplexing and quantitative attributes of mDIA should be of great advantage in tissue proteomics, especially in the context of diseases. In particular, we wanted to integrate it with our recent technology termed Deep Visual Proteomics (Mund *et al*, 2022). DVP combines artificial intelligence‐driven image analysis of cellular phenotypes with automated single‐cell laser microdissection and ultra‐high‐sensitivity mass spectrometry, effectively linking protein abundance to complex cellular phenotypes while preserving the spatial context. To explore the advantages of mDIA in this context, we profiled cancer cells in‐situ in primary cutaneous melanoma. Using routine histopathological markers, we segmented single melanoma cells and further stratified them according to their spatial location to the epidermal or dermal compartment, thereby taking the tumor microenvironment into account (Fig 7A). In recent work on superficial spreading melanoma, we had cut 700 shapes from 2.5 μm‐thin formalin‐fixed paraffin‐embedded (FFPE) tissue sections (Mund *et al*, 2022). We found that the mDIA workflow seamlessly integrated into the DVP pipeline. For the design of the reference channel, we used bulk digest from a consecutive tissue slide, containing mainly but not exclusively tumor material. We expected mDIA to be much more sensitive in this context and therefore only excised 100 single cell shapes (corresponding to 20 cell equivalents at 2.5 μm thickness). We added the reference channel to the epidermal and dermal target channels in a roughly estimated ten‐fold excess. With a Whisper20 SPD gradient (58 min active gradient), we quantified 4,000 protein groups in melanoma cells within the epidermis and 2,740 in the dermis, similar to our previous report on the same cell type. However, our mDIA‐DVP pipeline used seven times less input amount, a shorter gradient (1 h vs. 2 h) and measured two samples per run. This represents an increased throughput of four‐fold for MS analysis without losing proteomic depth and a seven‐fold decreased cutting time on the laser microdissection instrument, a large gain for the overall DVP pipeline.

To test the cell type‐specific and biological validity, we looked for typical melanocytic markers (SOX10, MITF, DCT, MLANA, PMEL, TYR, TYRP1) (Belote *et al*, 2021) in our data. We detected all but TYRP1, including SOX10, the most important (transcription) factor of melanocytic lineage used in routine clinical diagnostics (Fig 7B). Interestingly, expression of these identity markers was lower in dermal melanoma cells, highlighting the role of the tumor microenvironment in cancer‐cell identity and dedifferentiation (Fig 7C). Next, we sought to assess proteomic differences between epidermal and dermal melanoma cells. In principal component analysis (PCA), component 1 clearly separated dermal from epidermal melanoma cells (Fig 7D). Importantly, this was irrespective of the target channel used for labeling individual replicates, which we ascertained by label swapping. Compared to melanoma cells in the epidermis, melanoma cells located in the dermal compartment had significantly higher expression of proteins involved in remodeling of the extracellular matrix (e.g., DPT, COL1A2, COL1A1, COL3A1, COL6A1, DCN, TGFBI, PRELP, DCN, FGA, FN1, and LUM) (Fig 7E). In contrast, melanoma cells of the dermal compartment had significantly lower expression of TACSTD2 and LGALS7, amongst others (Fig 7E). While downregulation of TACSTD2 is part of a gene expression profile signature that predicts increased risk of cancer progression, LGALS7 is involved in promoting cellular apoptosis and could therefore lead to increased tumor cell survival (Biron‐Pain *et al*, 2013; Gerami *et al*, 2015). The protein Keratin 15 (KRT15), which is normally expressed in basal keratinocytes of the epidermis, has also been found to be associated with tumor stage and prognosis in metastatic melanoma patients, with higher expression in primary tumors and loss in metastases (Han *et al*, 2021). We also observed a reduced expression of KRT15 in cells located in the dermal compared to the epidermal melanoma cells according to the depth of invasion (Fig 7E). Pathway enrichment revealed multiple and highly relevant signaling cascades enriched in dermal melanoma cells, such as senescence, PI3K‐AKT signaling, and IL‐18 signaling (Fig 7F). Taken together, the combination of mDIA and DVP enables high‐throughput, in‐depth and biologically relevant insights of cancer cells from single patients, including the spatial component.

## Discussion

Here, we described a robust and economical mDIA workflow that we explored using standard proteomics samples and single cells. One of its main advantages is that it uses dimethyl labeling, a well‐established, very simple and robust technology (Hsu *et al*, 2003; Boersema *et al*, 2009). We consistently achieved labeling efficiencies of more than 99% for peptides generated by trypsin and Lys‐C – proteases typically employed in proteomics. The labeling protocol consists of only three pipetting steps, hence it was easily automatable on a standard liquid handling robot. The differentially labeled peptides are combined into one stage‐tip for purification and storage, thus avoiding sample loss. Another practical advantage is that the reagents themselves are very stable, whereas broadly used NHS (N‐Hydroxysuccinimide)‐chemistry‐based reagents such as mTRAQ or TMT are more labile.

Dimethyl labeling enables quantification at both the MS1 and MS2 level, but is limited to three‐plex when used in combination with trypsin. Here we show that Lys‐N digestion followed by dimethyl labeling accommodates a five‐plex format at both levels as well, with the interesting property that b‐ions are split up into five quantifiable states separated by 4 Da. The y‐ions are less intense due to the absence of a C‐terminal lysine, however, this is compensated for identification purposes by the fact that y‐ions from all channels stack on top of each other. This will be particularly interesting in the context of fragmentation with Electron Transfer Dissociation (ETD), as this has been shown to generate nearly pure c‐ion series in many cases on triply charged precursors (Hennrich *et al*, 2010). It might also be interesting if synchro‐PASEF (Skowronek *et al*, 2022a) can distinguish y‐ions from different channels by different slicing positions of the precursor‐fragment pair, since the different channels slightly differ in their ion mobility versus m/z position. More generally, mDIA should fully benefit from new scan modes and different mass spectrometry platforms.

Current software tools do not yet make optimal use of dimethyl mDIA data. Unlike ^13^C‐based labels, dimethyl labels can slightly influence retention times on LC columns. Our results show that this is not a major impediment to quantification, but we believe that results will improve once the retention times are directly modeled and corrected for. We have begun to do this with AlphaPeptDeep (Zeng *et al*, 2022), which we also used to derive the predicted DIA libraries.

While proteome depth is not compromised by dimethyl labeling per se, we did notice a drop in identifications for multiplexed as opposed to single proteomes (although overall identifications and quantifications are still vastly higher). As this correlates with the number of labeled channels, we attribute it to the increased complexity of the DIA data which will eventually lead to an overlap in DIA transitions. At least some of this drop in identifications should be retrievable by further optimization of the DIA software as the extra complexity follows a clearly defined pattern in the channel spacing. In tri‐plex format and combined with the relatively short gradients on the Evosep instruments, this achieves a throughput of 3 × 60 samples per day for proteome measurements, which would make it quite practical to incorporate an up‐front fractionation step in the future.

We also explored the idea of a reference channel in single‐cell mDIA. Note that this is conceptually different from the carrier channel employed in the SCoPE‐MS method (Budnik *et al*, 2018) because the fragments of the mDIA channels are offset from each other and do not contribute to a common low reporter mass (Derks *et al*, 2022). Existing DIA software such as Spectronaut or DIA‐NN, which we used here, do not explicitly make use of the reference channel yet, and the concept of a separate FDR for individual target channels is not fully developed yet. Nevertheless, we found that identifications roughly doubled, from just above thousand proteins for interphase cells in our previous single‐cell publication (Brunner *et al*, 2022) to a median of 2,370 now. Encouragingly, almost 4,000 proteins were identifiable for some of the cells. When combined with further ongoing instrumental and sample preparation advances, this may enable characterization of more than half of the expressed proteome in single cells in the near future. For our single‐cell analysis, we use the low flow, high sensitivity Whisper gradients on the Evosep, leading to a throughput of 80 single cells per day when using a reference channel, which would increase to 160 per day or 1,000 per week with the Lys‐N five‐plex method (subtracting one channel for the reference).

We are currently exploring the applicability of mDIA for a wide variety of biological hypotheses and projects that can now be addressed with proteomics. In regular analyses, we have found that the method retains most of the advantages of DIA, while tripling the throughput at little if any extra cost, as others have found as well (Derks *et al*, 2022). When transferred to our DVP technology (Mund *et al*, 2022), mDIA is especially attractive because different cell states that are spatially separated can be directly compared to each other. Furthermore, we found that the exquisite sensitivity of mDIA with a reference channel allows us to drastically reduce the number of shapes. Rare cell types can now rapidly be profiled at great proteomic depth and increased spatial resolution. Importantly, this also enables the analysis of true single cells in a spatial context (preprint: Rosenberger *et al*, 2022). Altogether, we believe that mDIA‐DVP will become a key enabling technology for precision oncology.

We look forward to further exploration of the reference channel concept. Combined with the inherent simplicity of the DIA acquisition method, it may lead to much more complete proteomes. Furthermore, the reference channel will make proteomic results much more comparable across experiments and between laboratories, something that should greatly benefit the community. This is applicable particularly for clinical cohorts like for example plasma proteomics (Bader *et al*, 2023). Together, the mDIA workflow and the reference channel opens up new opportunities for single‐cell proteomics and clinical proteomics.

## Materials and Methods

### Reagents and Tools table

**Table:** 

Reagent/Resource	Source	Identifier
**Chemicals, Enzymes, and other reagents**		
Bovine Serum Albumin (BSA)	New England BioLabs Inc.	P8108S
HeLa tryptic digest	Pierce	1862824
Yeast (*S. cerevisiae*) tryptic digest	Promega	V746A
*E. coli* tryptic digest	Waters	186003196
Triethylammonium bicarbonate (TEAB)	Sigma Aldrich	T7408
Ammonium bicarbonate (ABC)	Merck	1.01131
Urea	Sigma Aldrich	U1250
Dithiothreitol (DTT)	Sigma Aldrich	D0632
Chloroacetamide (CAA)	Sigma Aldrich	C0267
Formaldehyde: ‘light’ (CH_2_O)	Sigma Aldrich	F8775
Formaldehyde‐d2‐solution: ‘intermediate’ (CD_2_O)	Sigma Aldrich	492620
Formaldehyde‐^13^C, d2‐solution: ‘heavy’ (^13^CD_2_O)	Sigma Aldrich	596388
Sodium cyanoborohydride: ‘light’ (NaBH_3_CN)	Sigma Aldrich	156159
Sodium cyanoborodeuteride: ‘heavy’ (NaBD_3_CN)	Sigma Aldrich	190020
Lichropur® ammonia solution, 25%	Merck	5.33003
LC–MS‐grade water	Fisher Chemical	W6‐4
Trifluoroacetic acid (TFA)	Sigma Aldrich	8.08260
Formic acid (FA)	Sigma Aldrich/Merck	#64‐18‐6
Acetonitrile	Sigma Aldrich/Merck	#75‐05‐8
CDS Empore C18 Extraction Disks	Empore	13‐110‐018
Trypsin	Sigma‐Aldrich	T6567
Lys‐C	FUJIFILM Wako	125‐05061
Lys‐N	ImmunoPrecise Antibodies (Europe) BV	L101
**Software**		
MaxQuant (2.0.1)	https://maxquant.org/	N/A
DIA‐NN (1.8.1)	https://github.com/vdemichev/DiaNN	N/A
RStudio (2022.07.2) and R (4.2.2)	https://posit.co/download/rstudio‐desktop/	N/A
Jupyter Notebook	https://jupyter.org/	N/A
RefQuant	https://github.com/MannLabs/refquant	N/A
**Other**		
384‐well plates	Eppendorf	#0030129547
Adhesive PCR Sealing Foil Sheets	Thermo Scientific	#AB‐0626
Bravo robot	Agilent	
ThermoMixer	Eppendorf	#460‐0223
Mastercycler X50h	Eppendorf	#63160000
NanoDrop One/OneC Microvolume UV–Vis Spectrophotometer	Thermo Fisher	@ND‐ONEC‐W
EvoSep One	EvoSep	#EV‐1000
Evotip Pure	EvoSep	#EV‐2011
timsTOF HT	Bruker Daltonik GmbH	N/A
timsTOF SCP	Bruker Daltonik GmbH	N/A
timsTOF Ultra	Bruker Daltonik GmbH	N/A
Orbitrap Exploris 480	Thermo Scientific	N/A
Column oven	Sonation lab solutions	#PRSO‐V2
Aurora Elite CSI	IonOpticks	AUR3‐15075C18‐CSI

### Methods and Protocols

#### Sample preparation of bulk samples

##### Bovine serum albumin

CAM‐modified tryptic digest of bovine serum albumin (New England BioLabs Inc.) was labeled with dimethyl using the in‐solution labeling protocol (Boersema *et al*, 2009). Briefly, 500 pmol of the BSA digest was reconstituted in 300 μl of 100 mM triethylammonium bicarbonate (TEAB, pH 7) buffer and aliquoted into three separate tubes. To each of these, 4 μl of 4% (vol/vol) formaldehyde (CH_2_O, CD_2_O or ^13^CD_2_O) and 4 μl of 600 mM sodium cyanoborohydride (NaBH_3_CN or NaBD_3_CN) were added sequentially. The samples were incubated at room temperature for 1 h on a bench‐top mixer. To quench the reaction, 16 μl of 1% (vol/vol) ammonia (NH_4_OH) was added to each tube. The solutions were then dried at room temperature using a speed vacuum concentrator, reconstituted in 200 μl of buffer A (0.1% formic acid), and desalted using in‐house prepared, C18 StageTips (Rappsilber *et al*, 2003). The solutions were then adjusted to have a concentration of 50 fmol/μl. Three different mixtures were prepared by combining the three channels (Δ0/Δ4/Δ8) in varying mixing ratios: Mix 1 (8.5 μl/1.0 μl/0.5 μl), Mix 2 (7 μl/2 μl/1 μl), and Mix 3 (5 μl/3 μl/2 μl). A 1 μl aliquot of each mix was injected into the Thermo Orbitrap Exploris 480™ in triplicates, corresponding to an injection amount of 50 fmol per replicate.

##### Tryptic HeLa

A similar in‐solution labeling protocol was followed for tryptic HeLa digests (Pierce #1862824) using 10 μg of starting material per channel. After C18 desalting, four different mixes were prepared: 62.5 ng/μl of label‐free solution, 62.5 ng/μl of 1‐channel (Δ0), 125 ng/μl of 2‐plex (1:1 Δ0/Δ4), and 187.5 ng/μl of 3‐plex (1:1:1 Δ0/Δ4/Δ8). A 2 μl aliquot of each mix was injected into the Thermo Orbitrap Exploris 480™ in triplicates and another 2 μl aliquot of each mix was injected into the Bruker timsTOF HT in triplicates. These correspond to injection amounts of 125 ng for unlabeled and one‐channel solutions, 250 ng for two‐plex solutions, and 375 ng for three‐plex solutions, per replicate.

##### Mixed species (human/yeast/*E. coli*)

For the mixed species experiment, three different mixtures with varying mixing ratios of HeLa tryptic digest (Pierce #1862824), *S. cerevisiae* tryptic digest (Promega V746A), and *E. coli* tryptic digest (Waters #186003196) were prepared prior to labeling: Mix 1 (5.20 μg/1.20 μg/1.60 μg Human(H)/Yeast(Y)/*E. coli*(E)), mix 2 (5.20 μg/2.40 μg/0.40 μg H/Y/E), and mix 3 (5.20 μg/0.40 μg/2.40 μg H/Y/E). Mix 1 was then labeled with Δ0, mix 2 with Δ4, and mix 3 with Δ8, following the in‐solution labeling protocol. After C18 desalting, each mixture was adjusted to 150 ng/μl using buffer A (0.1% formic acid) and the three labeled mixtures were combined with each other in a 1:1:1 ratio. A 3 μl aliquot of each mixture was injected into the Bruker timsTOF HT in triplicates for each of the two acquisition methods (20S and 16S4MS1, S = dia‐PASEF scan) tested in this study. This corresponds to an injection amount of 450 ng total per replicate for each acquisition method.

##### Sample preparation of mixed species for FDR estimation

To empirically determine the false discovery‐rate (FDR), 4 μg S. cerevisiae tryptic digest (Promega V746A) and HeLa tryptic digest (Pierce #1862824) each was mixed together and labeled with Δ0. Additionally, 4 μg yeast tryptic peptides were labeled with Δ4 and Δ8, respectively. After C18 desalting, each solution was adjusted to 100 ng/μl using buffer A (0.1% formic acid) and the three labeled solutions were then mixed together in a 2:1:1 (Δ0/Δ4/Δ8) ratio to have equally distributed amount of yeast in each channel. A 4 μl aliquot of each mixture was injected into the Bruker timsTOF HT in triplicates, corresponding to an injection amount of 400 ng per replicate.

##### Lys‐N‐derived HeLa

The Lys‐N digest was prepared in‐house. A HeLa cell pellet was lysed in 8.0 M urea, 50 mM ABC pH 8.5 and incubated in a Bioruptor for 10 min. Reduction of disulfide bonds was performed by adding dithiothreitol (DTT) to a final concentration of 10 mM DTT and incubating at room temperature for 45 min. Carbamidomethylation of the free sulfhydryl groups was achieved by adding chloroacetamide (CAA) to a final concentration of 40 mM CAA and incubating in the dark at room temperature for 15 min. The reaction was then quenched with DTT to a final concentration of 10 mM DTT. The resulting lysate was diluted to ensure that the concentration of urea in the solution is less than 2.0 M (diluted to 1.0 M). Lys‐N (ImmunoPrecise Antibodies) was added to the lysate in a 1:100 (enzyme/protein) ratio and the digestion was left to digest at 37°C overnight. The mixture was acidified to a final concentration of 1% TFA to quench the digestion and desalted using in‐house prepared, C18 stage tips. The same in‐solution labeling protocol was used for labeling 25 μg of HeLa Lys‐N peptides per channel. Five different mixes were prepared from the labeled peptides: 50 ng/μl of unlabeled solution, 50 ng/μl of one‐channel (Δ0), 100 ng/μl of two‐plex (1:1 Δ0/Δ4), 150 ng/μl of three‐plex (1:1:1 Δ0/Δ4/Δ8), and 250 ng/μl of five‐plex (1:1:1:1:1 Δ0/Δ2/Δ4/Δ6/Δ8). A 2 μl aliquot of each mix was injected into the Bruker timsTOF HT in triplicates. These correspond to injection amounts of 100 ng for unlabeled and one‐channel solutions, 200 ng for two‐plex solutions, 300 ng for three‐plex solutions, and 500 ng for 5‐plex solutions, per replicate. For the 5‐plex accuracy test, purified labeled HeLa samples were mixed together in a ratio of 1:2:4:2:1 (Δ0, Δ2, Δ4, Δ6, Δ8) and a total of 500 ng was used per injection.

#### Sample preparation for reference channel and single‐cell equivalents

HeLa cells were cultured in Dulbecco's modified Eagle's medium at 10% fetal bovine serum, 20 mM glutamine and 1% penicillin–streptomycin. The supernatant was carefully removed, cells were detached by accutase treatment for 5 min at 37°C followed by pipetting for cell aggregate dissociation. Cells were washed three times with ice‐cold PBS, lysed by 60 mM TEAB and 20% acetonitrile (ACN) at 72°C for 30 min. Proteins were digested by trypsin and Lys‐C in a 1:50 enzyme:protein ratio. Peptides were cleaned up by C18 desalting as described above. Cleaned up peptides were labeled with light dimethyl (Δ0) as described above in 100 μl 60 mM TEAB (pH 8.5) to be comparable to the single‐cell workflow. Evotips were loaded as described in the single‐cell experiments.

#### Sample preparation for single‐cell experiments

HeLa cells were cultured and harvested following standard protocol as above. Cells were washed three times with ice‐cold PBS, pelleted by centrifugation and resuspended in ice‐cold PBS to a solution of 2 × 10^6^ cells per ml. Two microliters propidium iodide (PI, BioRad, 1351101) was added to the single‐cell suspension and sorting was performed on the PI‐negative live cell population using fluorescent‐activated cell sorting (FACS). Single cells were sorted into 384‐well plates containing 1 μl of 20% ACN, 0.01% n‐Dodecyl β‐D‐maltopyranoside (DDM), 60 mM TEAB pH 8.5, centrifuged briefly, sealed with aluminum seal sheets and frozen at −80°C until further processing. Single cells were incubated in the 384‐well plate for 30 min at 72°C in a PCR cycler with a lid temperature of 110°C, digested overnight at 37°C (lid temperature 55°C) after addition of 1 μl of 20% ACN, 0.01% DDM, 60 mM TEAB and 0.5 ng trypsin and Lys‐C each. Digested peptides were derivatized with dimethyl by adding 1 μl 0.6% formaldehyde (final concentration 0.15%) and 1 μl 92 mM cyanoborohydride (final concentration 23 mM). Single cells in a consecutive row pattern (B, D, F, …) were labeled with intermediate dimethyl (∆4) using intermediate formaldehyde (CD_2_O) and light cyanoborohydride, while other wells (C, E, G, …) were labeled with heavy dimethyl (∆8) using heavy formaldehyde (^13^CD_2_O) and heavy cyanoborohydride. After 1 h incubation at room temperature, the dimethyl labeling reaction was stopped by adding 1 μl 0.65% ammonia solution (final concentration 0.13%). Before Evotip loading, samples were acidified by 1 μl 6% TFA (final concentration 1%). All pipetting steps were done with a Bravo robot (Agilent). Evotips Pure were loaded with the Bravo robot (Agilent), by activation with 1‐propanol, washing two times with 50 μl buffer B (99.9% ACN, 0.1% FA), activation with 1‐propanol and two wash steps with 50 μl buffer A (99.9% H_2_O, 0.1% FA). In between Evotips were spun at 700 *g* for 1 min. For sample loading, Evotips were prepared with 70 μl buffer A and a short spin at 700 *g*. Ten microliters of 1 ng/μl prepared and aliquoted reference channel proteome (labeled with light dimethyl, ∆0) was pipetted into Evotips, followed by an intermediate and heavy dimethyl labeled single cell with the same tip of the Bravo robot to avoid plastic contacts. Single‐cell wells were washed with 15 μl buffer A and also loaded onto the Evotips. Evotips were spun at 700 *g* to load the sample and washed ones with 50 μl buffer A and stored at 4°C with buffer A on top until measured.

#### Multiplexed‐DIA Deep Visual Proteomics (mDIA‐DVP)

##### Specimen preparation and immunofluorescence staining

Primary cutaneous melanoma (subtype superficial spreading melanoma, Breslow‐depth 2.5 mm) was obtained as formalin‐fixed paraffin‐embedded (FFPE) tissue according to the institutional review board protocol (BASEC. Nr:2014‐0425) at the University Hospital of Zurich, Switzerland. The tissue was collected in accordance with the Declaration of Helsinki following standard operating procedures. 2.5 μm thin tissue sections were mounted on Polyethylen‐Naphthalat (PEN) membrane slides (MicroDissect GmbH, MDG3P40AK), coated with Vectabond (Biozol, VEC‐SP‐1800). Tissue specimens were processed as described previously (Mund *et al*, 2022; preprint: Nordmann *et al*, 2023). In brief, slides were deparaffinized (2 min xylene, 1 min 100% EtOH, 95% EtOH, 75% EtOH, 30% EtOH, and ddH20, repeated twice, respectively) and glycerol‐supplemented heat induced epitope‐retrieval (G‐HIER) (Tris/EDTA pH 9 HIER buffer (DAKO, S2367), 10% glycerol (v/v; Sigma, G7757)) was performed for 20 min at 88°C. Thereafter, unspecific binding sites were blocked using 5% bovine serum albumin (BSA) in PBS for 30 min at RT, followed by the application of primary antibodies for CD44 (rabbit, Abcam ab51037; 1:200) and SOX10 (mouse, NordicBiosite bsh‐7959‐1; 1:50) for 90 min in a humidified staining chamber. Secondary antibodies against mouse‐IgG (Alexa‐647, Invitrogen A32728, 1:400) and rabbit‐IgG (Alexa‐555, Invitrogen A32732, 1:400) were applied for 1 h, followed by 7 min incubation with SYTOX green Nucleic Acid Stain (Invitrogen S7020; 1:700 in ddH20). Potential membrane distortions were released using a needle (30G) and sealed thereafter, as described previously (preprint: Nordmann *et al*, 2023). Cover glasses were mounted using Slowfade Diamond Antifade Mountant (Invitrogen, S36967) and removed after high‐resolution imaging in ddH20. All staining steps were performed at room temperature unless otherwise specified.

##### Artificial intelligence (AI)‐based image analysis and cell extraction

High‐content images of mounted tissue specimens were acquired on a Zeiss Axioscan Z7 at 20× magnification with a 10% tile overlap. Resulting data were submitted to AI‐based cell segmentation and classification using the Biology Image Analysis Software (BIAS, Cell Signaling). Cell outlines were defined based on CD44 staining and supervised machine learning (Mode: Deep Neural Network) to further stratify melanoma cells according to SOX10 positivity. Epidermal and dermal location was specified by manual region annotation. Contours of identified cells were transferred to a LMD7 laser microdissection instrument (Leica Microsystems) and cell registration was achieved using three reference points in the tissue regions of interest. Cells were extracted using the middle pulse function and an outline offset of 0.25 μm into a 384‐well plate by gravity. Samples were collected in quadruplicates for the respective number of contours.

##### Sample processing for mDIA‐DVP

DVP samples were processed on a liquid handling platform (Bravo, Agilent Technologies). Extracted cells were collected at the bottom of each well by acetonitrile‐supported centrifugation and vacuum evaporation until dryness. Thereafter, lysis was accomplished in 6 μl 70 mM TEAB, 0.013% DDM and incubation at 95°C for 60 min, complemented by additional 60 min at 75°C in 12% (v/v) acetonitrile (by adding 1 μl 80% ACN). Proteins were digested using 4 ng LysC and 6 ng trypsin overnight at 37°C and labeled as described above. For the reference channel, a consecutive tissue slide – containing mainly but not exclusively tumor material – was collected from a PEN membrane slide into a 96‐well plate (Covaris) using a scalpel and processed analogous to the DVP samples using 42 μl of lysis buffer and additional sonification (LE220‐plus: peak Power: 450.0, duty factor: 50%, cycles: 200, average power: 225, Covaris). The following steps of digestion, purification and labeling were performed as described above in sample prepartion of the reference channel. Evotip loading was accomplished as described above using 25 ng of the bulk tissue as reference channel (∆0) combined with the target channels. Two replicates of each melanoma type were measured as target ∆4 and ∆8, respectively.

#### Acquisition of bulk data

All samples were injected using the EASY‐nLC 1200 (Thermo Scientific) and separated via in‐house pulled columns (50 cm length, 75 μm ID) packed with 1.9 μm ID ReproSil‐Pur 120 C18‐AQ (Dr. Maisch GmbH). These samples were sprayed into either an Orbitrap Exploris 480™ (Thermo Scientific) or a timsTOF HT (Bruker Daltonics) for MS analysis. Buffer A is 0.1% formic acid in LC–MS‐grade water. Buffer B is 80% acetonitrile in LC–MS‐grade water with 0.1% formic acid.

##### Bovine serum albumin

For BSA samples, a 30‐min active gradient was used as follows: 2–7% Buffer B (minutes 0–1), 7–30% Buffer B (minutes 1–15), 30–65% Buffer B (minutes 15–18), 65–95% Buffer B (minutes 18–21), 95% Buffer B (minutes 21–24), 95–5% Buffer B (minutes 24–27), and 5% Buffer B (minutes 27–30). The flow rate was kept constant at 300 nl/min. *DDA*. For determining the labeling efficiency of each dimethyl channel, shotgun DDA was employed on the Thermo Orbitrap Exploris 480™. The MS1 full scan range was 300–1,650 m/z with a resolution of 60,000, normalized automatic gain control (AGC) target of 300%, and a maximum injection time of 25 ms. This shotgun DDA approach was filtered for an intensity threshold of 5,000, with inclusion of charge states 2–5 and a dynamic exclusion duration of 30 s. There are five data dependent MS2 scans with a resolution of 15,000, normalized AGC target of 100%, maximum injection time of 28 ms, 1.4‐Th isolation window width, and 27% normalized HCD collision energy. *DIA*. The DIA mode consists of one MS1 full scan followed by 16 MS2 windows with variable widths (Appendix Table S1). Each MS1 full scan (300–1,650 m/z) was conducted at 120,000 resolving power, 300% normalized AGC target, and 100 ms maximum injection time. Each MS2 scan was conducted at 30,000 resolving power, 3,000% normalized AGC target, and 30% normalized HCD collision energy with a default charge of 2. The RF lens was set to 40%.

##### Tryptic HeLa

For the tryptic HeLa digests, a 75 min active gradient was used as follows: 2–7% Buffer B (minutes 0–1), 7–30% Buffer B (minutes 1–60), 30–50% Buffer B (minutes 60–66), 50–60% Buffer B (minutes 66–70), 60–90% Buffer B (minutes 70–71), and 90% Buffer B (minutes 71–75). The flow rate was kept constant at 300 nl/min. *Orbitrap DDA*. For determining the labeling efficiency of each dimethyl channel, shotgun DDA was employed on the Thermo Orbitrap Exploris 480™. The MS1 full scan range was 300–1,650 m/z with a resolution of 60,000, normalized automatic gain control (AGC) target of 300%, and a maximum injection time of 25 ms. This shotgun DDA approach was filtered for an intensity threshold of 100,000, with inclusion of charge states 2–5 and a dynamic exclusion duration of 30 s. There are 15 data dependent MS2 scans with a resolution of 15,000, normalized AGC target of 100%, maximum injection time of 28 ms, 1.4‐Th isolation window length, and 30% normalized HCD collision energy. *Orbitrap DIA*. The DIA mode consists of one MS1 full scan followed by 44 MS2 windows with variable widths (Appendix Table S2) (Steger *et al*, 2021). Each MS1 full scan (300–1,650 m/z) was conducted at 120,000 resolving power, 300% normalized AGC target, and 100 ms maximum injection time. Each MS2 scan was conducted at 30,000 resolving power, 3,000% normalized AGC target, and normalized HCD collision energy in stepped mode of 25%, 27.5%, and 30%. The default charge was 3 and the RF lens was set to 40%. *timsTOF dia‐PASEF*. For the dia‐PASEF measurements on the Bruker timsTOF HT platform, one MS1 full scan was followed by 20 dia‐PASEF scans with variable widths that were optimized for the precursor densities of tryptic HeLa digests using py_diAID (Skowronek *et al*, 2022b). The method covers an m/z range from 300 to 1,200 with two IM windows per dia‐PASEF scan ranging from 0.3 to 1.7 Vs cm^−2^ (Appendix Fig S3A, Appendix Table S3). Since the MS1 scan and each dia‐PASEF scan measures 100 ms, the total cycle time for this method is 2.1 s. The collision energy is a linear ramp from 20 eV at 1/K0 = 0.6 Vs cm^−2^ to 59 eV at 1/K0 = 1.6 Vs cm^−2^.

##### Mixed species experiment (human/yeast/*E. coli*)

The mixed species experiment was performed using the same gradient as the one used for tryptic HeLa digests. They were mainly measured in dia‐PASEF mode on the Bruker timsTOF HT platform using two methods, hereby referred to as MS2‐centric method (20S) and MS1‐centric method (16S4MS1). The MS2‐centric method has the same settings as the one described for tryptic HeLa digests where one MS1 scan is followed by 20 dia‐PASEF scans (Appendix Fig S3A). The MS1‐centric method was also optimized using py_diAID to cover an m/z range from 300 to 1,200 with two IM windows per dia‐PASEF scan ranging from 0.3 to 1.7 Vs cm^−2^ (Appendix Fig S3B). In this method, there are 4 ⨉ (1 MS1 scan + 4 dia‐PASEF scans) that also leads to a total cycle time of 2.0 s (Appendix Fig S3B, Appendix Tables S4 and S5). The collision energy is a linear ramp from 20 eV at 1/K0 = 0.6 Vs cm^−2^ to 59 eV at 1/K0 = 1.6 Vs cm^−2^.

##### Lys‐N‐derived HeLa

All Lys‐N‐derived HeLa measurements were acquired on the Bruker timsTOF HT using the 75‐min gradient used for the tryptic HeLa digests. They were mainly measured in dia‐PASEF mode using an MS2‐centric method (20S) optimized for Lys‐N‐derived HeLa peptides (Appendix Fig S3C, Appendix Table S6). The 5‐plex accuracy experiment was measured on the EvoSep One system using the 30SPD method (44 min active gradient) on a 15 cm × 150 μm column with 1.9 μm C18‐beads (PepSep) at 40°C. The analytical column were connected to a fused silica ID emitter (10 μm ID; Bruker Daltonics).

#### LC–MS/MS analysis of ultra‐high sensitivity and single‐cell data

All samples were loaded onto Evotips Pure and measured with the Evosep One LC system (EvoSep) coupled to a timsTOF SCP mass spectrometer (Bruker). The Whisper40 SPD (samples per day) method was used with the Aurora Elite CSI third generation column with 15 and 75 μm ID (AUR3‐15075C18‐CSI, IonOpticks) at 50°C inside a nanoelectrospray ion source (Captive spray source, Bruker). The mobile phases comprised 0.1% FA in LC–MS‐grade water as buffer A and 99.9% ACN/0.1% FA as buffer B. The timsTOF SCP was operated in dia‐PASEF mode with variable window widths. Optimal dia‐PASEF methods cover the precursor cloud highly efficient in the m/z – ion mobility (IM) plane while providing deep proteome coverage. For method generation with py_diAID, the precursor density distribution in m/z and IM was estimated based on a tryptic 48 high‐pH fraction library (Skowronek *et al*, 2022b). We calculated the optimal cycle time based on the chromatographic peak width of 5 ng HeLa single runs. DIA‐NN reported a base‐to‐base peak width of 7.6 s, translating into eight data points per peak at a cycle time of 0.96 s. The optimal dia‐PASEF method consisted of one MS1 scan followed by eight dia‐PASEF scans with two IM ramps per dia‐PASEF scan, covering a m/z range from 300 to 1,200 and IM of 0.7 to 1.3 Vs cm^−2^ (Appendix Fig S3D, Appendix Table S7). The mass spectrometer was operated in high sensitivity mode, the accumulation and ramp time was specified as 100 ms, capillary voltage was set to 1,400 V and the collision energy was a linear ramp from 20 eV at 1/K_0_ = 0.6 Vs cm^−2^ to 59 eV at 1/K_0_ = 1.6 Vs cm^−2^.

For the mDIA‐DVP (mDVP) experiment, a timsTOF Ultra mass spectrometer (Bruker) was used in combination with the Whisper20 SPD method. The optimal dia‐PASEF method consisted of one MS1 scan followed by twelve dia‐PASEF scans with two IM ramps per dia‐PASEF scan, covering a m/z range from 350 to 1,200 and IM of 0.7 to 1.3 Vs cm^−2^ (Appendix Table S8). All other parameters were kept the same.

The label efficiency of 1 ng HeLa peptides was assessed on the timsTOF SCP operating in dda‐PASEF mode with ten PASEF/MSMS scans per topN acquisition cycle. Singly charged precursors were excluded by their position in the m/z‐IM plane using a polygon shape, and precursor signals over an intensity threshold of 1,000 arbitrary units were picked for fragmentation. Precursors were isolated with a 2 Th window below m/z 700 and 3 Th above, as well as actively excluded for 0.4 min when reaching a target intensity of 20,000 arbitrary units. All spectra were acquired within a m/z range of 100–1,700. All other settings were as described above.

#### Raw data analysis with MaxQuant for label efficiency assessment

MaxQuant version 2.0.1 was used to process up to quintuplicate DDA data for calculating the labeling efficiency of each dimethyl channel. All dimethyl groups (Var DimethNter0, Var DimethLys0, Var DimethNter2, Var DimethLys2, Var DimethNter4, Var DimethLys4, Var DimethNter6, Var DimethLys6, Var DimethNter8, and Var DimethLys8) were first configured as variable modifications in MaxQuant. They were selected as variable modifications together with Oxidation (M) and Acetyl (Protein‐N‐term). For example, BSA samples labeled with dimethyl Δ0 were searched with Var DimethNter0, Var DimethLys0, Oxidation (M), and Acetyl (Protein‐N‐term) as variable modifications. Carbamidomethyl (C) was selected as a fixed modification for bulk but unselected for ultra‐high sensitivity acquisitions, since peptides were not reduced and alkylated. The maximum number of modifications per peptide was set to 3. Depending on the sample, either trypsin/P or Lys‐N was selected as the protease and the maximum number of missed cleavages was set to 2. Labeling efficiency was calculated based on intensity ratios of labeled peptides relative to all detected peptides.

#### Raw data analysis with DIA‐NN

DIA‐NN version 1.8.1 was used to search DIA raw files and dia‐PASEF files for precursor and fragment identifications based on 2D or 3D peak position (retention time, m/z precursor, and IM) using AlphaPeptDeep‐predicted spectral libraries (*dimethyl labeled tryptic HeLa: 6331933 precursor entries*, *label‐free tryptic HeLa: 6341464 precursor entries*, *dimethyl labeled tryptic Human/Yeast/E.Coli mix: 9716057 precursor entries*, *dimethyl labeled Lys‐N‐derived HeLa: 3387578 precursor entries*, *dimethyl labeled tryptic HeLa for ultra‐high sensitivity samples: 6294089 precursor entries*).

The DIA‐NN search included the following settings: Protein inference = ‘Genes’, Neural network classifier = ‘Single‐pass mode’, Quantification strategy = ‘Robust LC (high precision)’, Cross‐run normalization = ‘RT‐dependent’, Library Generation = ‘IDs, RT and IM Profiling’, and Speed and RAM usage = ‘Optimal results’. Mass accuracy and MS1 accuracy were set to 0 for automatic inference from the first run. The following settings were also enabled: ‘Use isotopologues’, ‘MBR’, ‘Heuristic protein inference’, and ‘No shared spectra’. For dimethyl labeled BSA and tryptic HeLa samples, the following additional commands were entered into the DIA‐NN command line GUI: *(1)* {‐‐fixed‐mod Dimethyl, 28.0313, nK}, *(2)* {‐‐channels Dimethyl, 0, nK, 0:0; Dimethyl, 4, nK, 4.0251:4.0251; Dimethyl, 8, nK, 8.0444:8.0444}, *(3)* {‐‐original‐mods}, *(4)* {‐‐peak‐translation}, *(5)* {‐‐ms1‐isotope‐quant}, *(6)* {‐‐report‐lib‐info}, and *(7)* {‐mass‐acc‐quant 10.0}. Note that *(7)* is removed from the command line for the MS1‐centric method. For dimethyl labeled Lys‐N‐derived HeLa samples, additional channels were inserted into the DIA‐NN command line GUI: {‐‐channels Dimethyl, 0, nK, 0:0; Dimethyl, 2, nK, 2.0126:2.0126; Dimethyl, 4, nK, 4.0251:4.0251; Dimethyl, 6, nK, 6.0377:6.0377; Dimethyl, 8, nK, 8.0444:8.0444}. For label‐free HeLa tryptic/Lys‐N samples, only the following commands were used: *(1)* {‐‐original‐mods}, *(2)* {‐‐ms1‐isotope‐quant}, *(3)* {‐‐report‐lib‐info} and *(4)* {‐mass‐acc‐quant 10.0}. In cases where only one channel or two channels were present, the actual number of channels were inserted into the DIA‐NN command line GUI and not the full set of channels. We used reannotate with the fasta SwissProt database of reviewed sequences (April, 2022).

Ultra‐high sensitivity measurements were searched in the same way as tryptic HeLa samples, except that the scan window was always set to a fixed value of 9, as well as MS1 and mass accuracy to 15 ppm, as this was identified as the optimal settings after several runs and kept constant for minimizing variance between the datasets. The optimal settings were evaluated by an independent search with ‘unrelated runs’ enabled. In cases of empty channels, the full set of channels were still added to the DIA‐NN command line GUI. For the single‐cell dataset and mDIA‐DVP, a combined library approach was used to speed up the searching time. A subset of the single‐cell dataset (50 raw files) or DIA runs (50 ng) of the reference channel sample were searched with same settings against the AlphaPeptDeep‐predicted library. The generated report‐lib library (file name: ‘report‐lib.tsv’, from ‘first‐pass’ search) was then used to analyze all raw files. The report‐lib library was adjusted by renaming the ‘Protein.Group’ column to ‘UniprotID’ and searched without the ‘reannotate’ function in DIA‐NN to keep the same protein grouping. The first‐pass‐result of this search was used for data analysis, since a MBR cycle was already performed in the report‐lib library generation.

#### AlphaPeptDeep library prediction

Libraries were predicted by AlphaPeptDeep using the same fasta file as described above. Predefined standard models of MS2, RT and CCS were refined by transfer learning using either MaxQuant, AlphaPept or DIA‐NN output files (refined models are uploaded to PRIDE and MassIVE, see Data availability). Peptide labeling was set to ‘Dimethyl@K’ and ‘Dimethyl@Any N‐term’ with a mass of 28.0313 (∆0). Please note that the library only needs to be predicted with one channel (in our case ∆0, as used for the reference channel), since DIA‐NN creates the library for the other channels on its own by the added commands. All other settings were used as standard. In short, ‘None’ was used as decoy method and trypsin was selected as protease. A maximum missed cleavage of 1 was allowed. ‘Carbamidomethyl@C’ was set as fixed modification (where appropriate) and ‘Oxidation@M’ as variable modification, with a maximum of one variable modification. The peptide length was set from 7 to 30, the precursor charge from 1 to 4 and the precursor m/z from 200 to 1800. b‐ and y‐ions were used as fragment types with a maximum fragment charge of 2. The top 12 fragments per precursor were kept from 200 to 2000 m/z. Predicted RT was converted to iRT and a tsv file was exported for DIA‐NN search.

#### Protein quantification for bulk data

The DIA‐NN R package was used to calculate the MaxLFQ abundance for protein groups (Demichev *et al*, 2019). The MaxLFQ abundance (Cox *et al*, 2014) was calculated based on the ‘Precursor.Normalised’ column output by DIA‐NN for MS2‐centric methods whereas it is based on the ‘Ms1.Area’ column output by DIA‐NN for the MS1‐centric method. For all datasets, the output results from the R package were then filtered for ‘Global.PG.Q.Value’ < 0.01 and ‘PG.Q.Value’ < 0.05. An additional filtering for ‘Channel.Q.Value’ < 0.01 was applied for labeled datasets and ‘Q.Value’ < 0.01 for label‐free datasets. For the mixed species three‐plex sample, the calculated protein ratios were normalized using the human protein ratios which are present in equal amounts in the different channels. To control the comparison, we used intersected protein groups between the two methods (*n* = 8,707).

#### RefQuant and its protein quantification

The filtered DIA‐NN output report table was processed with the ‘RefQuant’ package implemented in Python. RefQuant (https://github.com/MannLabs/refquant) imports the DIA‐NN table and extracts the relevant information used for quantification (quantities used: ‘Fragment.Quant.Raw’, ‘Ms1.Area’, ‘Precursor.Translated’, ‘Precursor.Normalised’). Subsequently, on each precursor, the following operations are performed: The target quantity is divided by the reference quantity for each of the available extracted quantities. This results in a list of ratios. The ratios are sorted in ascending order and the first 40% of ratios are retained. The overall ratio R between target and reference is then obtained by taking the mean of the remaining ratios. The ratio R is then multiplied by a precursor‐specific scaling factor, representing the intensity in the reference channel, which results in an overall intensity estimate of the precursor in the target channel. The scaling factor for each precursor is derived by taking the median of the reference intensity over all available runs for this particular precursor. The default value for the reference intensity is derived from the ‘Ms1.Area’. In case there are remaining precursors with less than two not‐null ‘Ms1.Area’ values, the scaling factor is derived from ‘Precursor.Translated’ or ‘Precursor.Normalised’. Otherwise, if there are still remaining precursors with less than two not‐null reference intensity values, the scaling factor is derived from the summed available ion intensities. Processing with RefQuant resulted in a quantification matrix (protein group, precursors, experiment and channel RefQuant intensity).

The RefQuant quantification matrix was then further processed with the iq package (Pham *et al*, 2020) in order to derive MaxLFQ protein quantities.

#### Proteomics downstream data analysis

Proteomics data analysis was performed in RStudio 2022.07.2 with R 4.2.2 and Python (version 3.8.2). MaxQuant output tables were filtered for ‘Reverse’, ‘Only identified by site modification’ and ‘Potential contaminants’ before further processing. For ultra‐high sensitivity measurements, the RefQuant output was filtered for ‘Lib.PG.Q.Value’ < 0.01, ‘Q.value’ < 0.01 and ‘Channel.Q.Value’ < 0.15. Seven hundred and sixty‐four cells were measured and 534 cells were not empty (above 1% of identified proteins). Cells in a range of 1.5 standard deviations around the median number of identified proteins were used for further analysis, except three outliers which showed a highly increased MS signal intensity compared to their proteomic depth (SM03p5b_30_5329_label4, SM03p4a_68_5210_label8, SM03p6a_65_5473_label8).

#### Comparison of single‐cell protein and RNA

The SMART‐Seq2 (Hu *et al*, 2019) dataset contained 720 HeLa cells with a total of 24,990 expressed genes and the Drop‐seq (Schwabe *et al*, 2020) dataset measured a total of 5,665 cells and 41,161 expressed genes, both measured as three batches. Single‐cell analysis was performed with scanpy v1.6.0 (Wolf *et al*, 2018) similar as before, while proteins were normalized based on the scepter ‘normalize’ method that iteratively adjusts the median protein expression between the different batches and different targets (Schoof *et al*, 2021). If not stated otherwise, standardized filtering across all datasets, removed cells with less than 600 genes expressed, and removed genes detected in < 15% of the remaining cells. This resulted in 10,557 transcripts in 720 cells in the SMART‐Seq2 dataset and 5,022 transcripts and 6,701 cells measured with Drop‐seq technology. The data completeness across covered dynamic range was computed as a function of the mean log(x + 1)‐transformed protein abundance of all non‐zero/‐NaN entries. The expected data completeness was included based on the assumption that missing values are purely due to shot‐(Poisson)‐noise as 1‐exp(−x). For correlation analysis, the RNA abundance entries were linearly scaled to sum to the mean cell size of the respective dataset per cell (231,281.56 for SMART‐Seq2 and 7,808.12 for Drop‐Seq). This was followed by log(x + 1) transformation of all abundance entries. Entries of missing protein abundance values were excluded from the specific computation. RNA expression vectors were scaled to the mean cell size of that measurement technology in the CV versus mean intensity plots comparing different technologies as well as the mean versus CV analysis (including the core proteome analysis) and the CV distribution boxplots. Mean and CV values were computed per gene under the assumption that scRNAseq data are not zero inflated (Svensson, 2020) while NaNs were excluded for the proteomics data. CV (RNA‐seq) versus CV (Proteomics) plots compared CV values of proteins/genes that were shared between all datasets.

## Author contributions

**Matthias Mann:** Conceptualization; formal analysis; supervision; funding acquisition; writing – original draft; project administration; writing – review and editing. **Marvin Thielert:** Conceptualization; data curation; software; formal analysis; supervision; validation; investigation; visualization; methodology; writing – original draft; project administration; writing – review and editing. **Ericka CM Itang:** Formal analysis; validation; investigation; writing – original draft; writing – review and editing. **Constantin Ammar:** Software; formal analysis; visualization. **Florian A Rosenberger:** Formal analysis; investigation. **Isabell Bludau:** Formal analysis; validation. **Lisa Schweizer:** Validation; investigation. **Thierry M Nordmann:** Formal analysis; investigation. **Patricia Skowronek:** Data curation; formal analysis. **Maria Wahle:** Formal analysis; validation; investigation. **Wen‐Feng Zeng:** Software. **Xie‐Xuan Zhou:** Software. **Andreas‐David Brunner:** Formal analysis. **Sabrina Richter:** Formal analysis; visualization; methodology. **Mitchell P Levesque:** Resources; formal analysis. **Fabian J Theis:** Formal analysis; supervision; visualization; methodology. **Martin Steger:** Conceptualization; formal analysis; supervision; investigation; writing – original draft; writing – review and editing.

## Disclosure and competing interests statement

MM is an indirect shareholder in EvoSep Biosystems. All other authors have no relevant competing interests. MM and FJT are editorial advisory board members. This has no bearing on the editorial consideration of this article for publication.

## Supporting information



Appendix

Expanded View Figures PDF

PDF+

Source Data for Figure 5

Source Data for Figure 6

## Data Availability

All mass spectrometry proteomics raw data, libraries, refined AlphaPeptDeep models for library prediction and outputs from each particular search engine analyzed in this study have been deposited to the ProteomeXchange Consortium via the PRIDE partner repository (Perez‐Riverol *et al*, 2019), Project accession: PXD038632 (https://www.ebi.ac.uk/pride/archive?keyword=PXD038632), and additionally, via the MassIVE partner repository, Project accession: MSV000090956 (https://massive.ucsd.edu/). The source code of RefQuant is openly available on GitHub: https://github.com/MannLabs/refquant. It will be made available through PyPI with “pip install RefQuant” as well. The code used for data analysis will be made publicly available upon publication on GitHub.
