# Supplementary material for: Robust dimethyl‐based multiplex‐DIA doubles single‐cell proteome depth via a reference channel
Source: Mol Syst Biol. 2023 Aug 21;19(9):e11503. doi: 10.15252/msb.202211503 (PMC10495816; doi:10.15252/msb.202211503)
Supplement: Supplementary file 1 — Appendix [file MSB-19-e11503-s001.pdf]

Appendix - Table of Content

Appendix Figures

Appendix Figure S1 - Quantification accuracy of bovine serum albumin (BSA)-derived tryptic peptides labeled with dimethyl mass tags and mixed at defined ratios. 2

Appendix Figure S2 - Labeling efficiency for tryptic and Lys-N-derived HeLa peptides. 3

Appendix Figure S3 - Optimal dia-PASEF acquisition method for dimethyl labeled peptides for tryptic and Lys-N-derived HeLa peptides... 4

Appendix Figure S4 - Quantitative accuracy and reproducibly of target channels in dependence of the reference channel amount using the scReference dataset. 6

Appendix Figure S5 - mDIA shows that the concept of a stable proteome is still valid at higher proteomic depth in single cells. 7

Appendix Tables

Appendix Table S1 - Variable window sizes for the Orbitrap DIA method used to measure dimethyl 3-plex tryptic BSA. 9

Appendix Table S2 - Variable window sizes for the Orbitrap DIA method used to measure dimethyl 3-plex tryptic HeLa. 10

Appendix Table S3 - 20S MS2-centric dia-PASEF method optimized for tryptic digests (See Appendix Fig S3A). 11

Appendix Table S4 - 16S4MS1 MS1-centric dia-PASEF method optimized for tryptic digests (See Appendix Fig S3B). 12

Appendix Table S5 - Overview of important parameters used in the MS2-optimized acquisition schemes for both Orbitrap and timsTOF and MS1-optimized for timsTOF. 13

Appendix Table S6 - 20S MS2-centric dia-PASEF method optimized for Lys-N HeLa digest (See Appendix Fig S3C). 13

Appendix Table S7 - Eight dia-PASEF scan method optimized for tryptic HeLa digest and ultra-high sensitivity experiments (See Appendix Fig S3D). 14

Appendix Table S8 - Twelve dia-PASEF scan method for mDIA-DVP on Whisper20 gradient. .15

Appendix Texts

Appendix Text S1 - Fundamental considerations about the noise in target and reference for ratio-based quantification 16

Appendix Figures

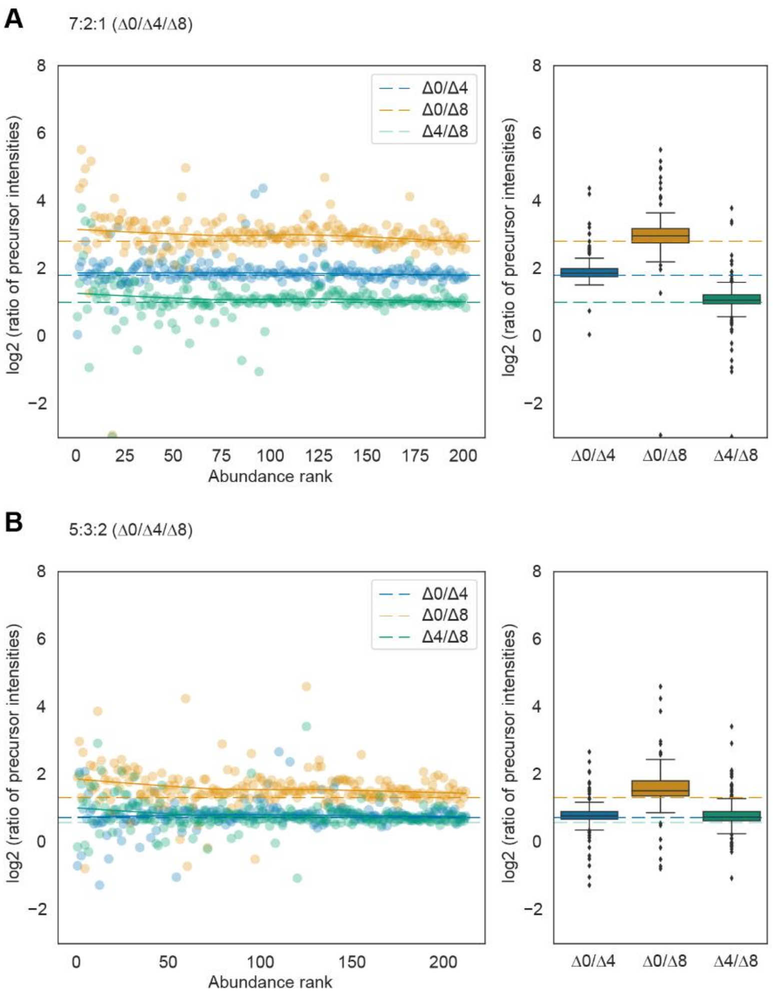

Appendix Figure S1 - Quantification accuracy of bovine serum albumin (BSA)-derived tryptic peptides labeled with dimethyl mass tags and mixed at defined ratios.

A and B. Quantification accuracy for peptides labeled with three mass tags and mixed in 7:2:1 (A) or 5:3:2 (B) ratios (technical replicates, n=3). Scatter plots on the left panel show the log2 intensity ratios as a function of the peptide abundance rank. In both panels, the expected ratios are marked by colored dashed lines. The box shows the interquartile range with the central band representing the median value of the dataset. The whiskers represent the furthest datapoint that is within 1.5 times the interquartile range (IQR).

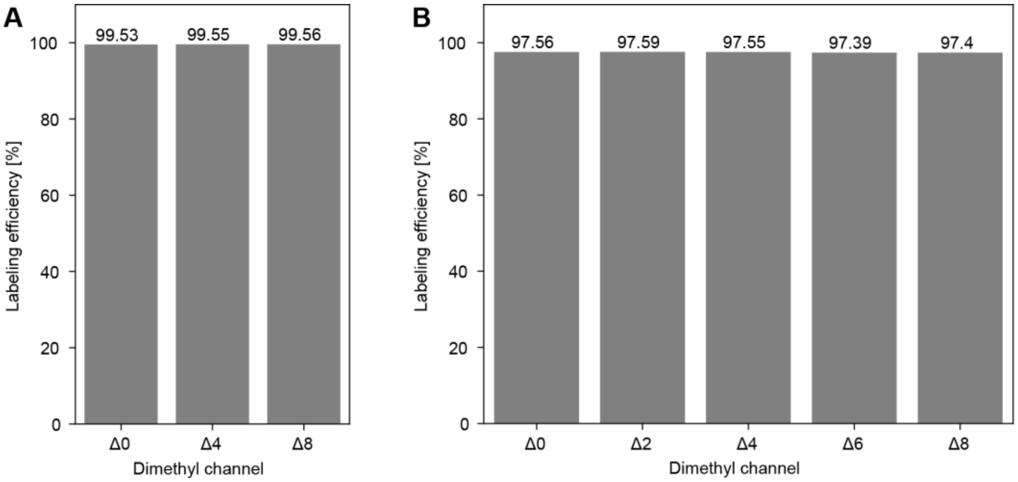

Appendix Figure S2 - Labeling efficiency for tryptic and Lys-N-derived HeLa peptides.

A. Tryptic peptides from HeLa cells were labeled with dimethyl mass tags Δ0, Δ4 and Δ8 and acquired individually in DDA mode (n=1).

B. Lys-N-derived HeLa peptides from HeLa cells were labeled with dimethyl mass tags Δ0, Δ2, Δ4, Δ6, and Δ8 and acquired individually in DDA mode (n=1).

Labeling efficiencies based on intensity ratios of labeled peptides relative to all detected peptides are shown.

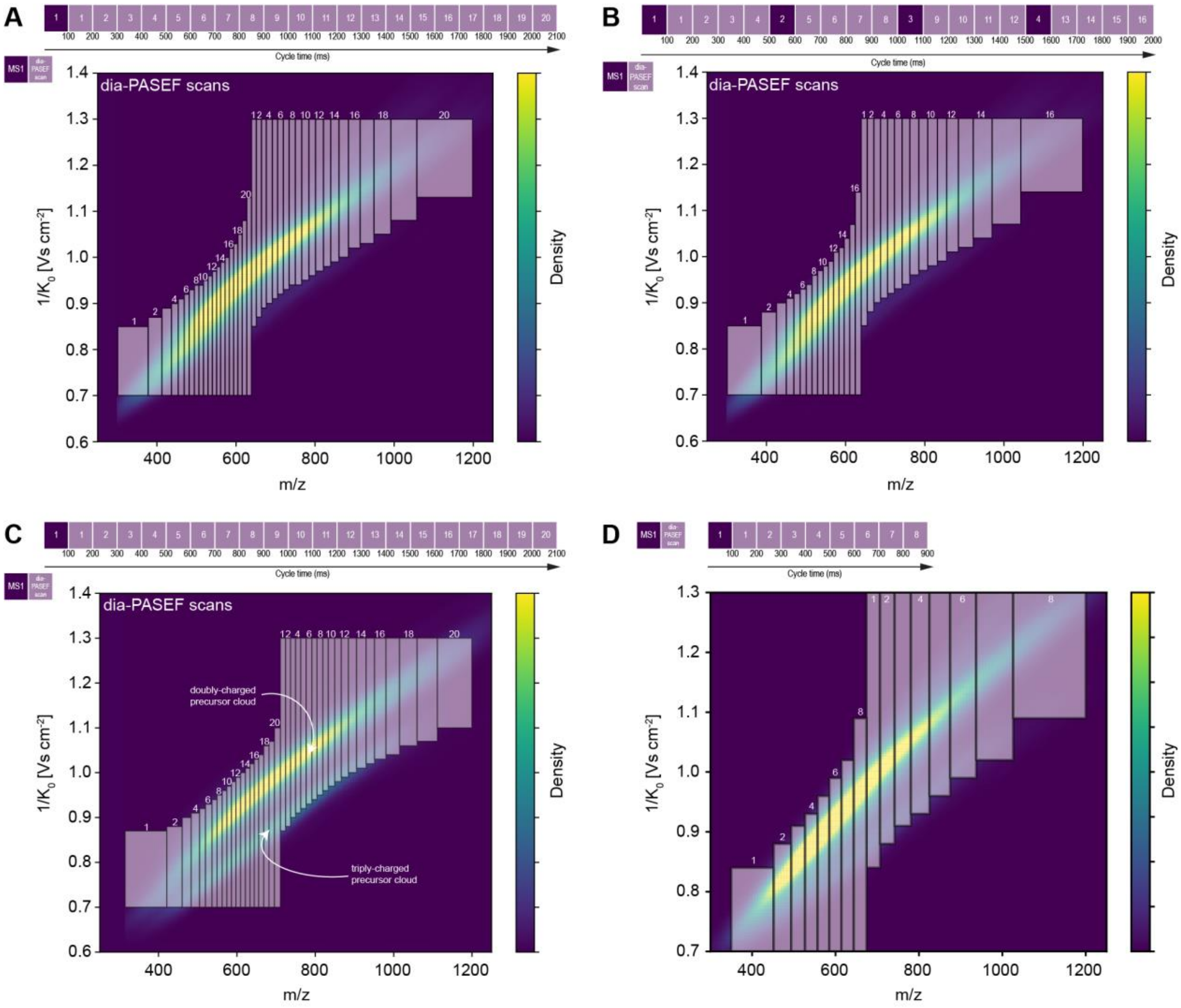

Appendix Figure S3 - Optimal dia-PASEF acquisition method for dimethyl labeled peptides for tryptic and Lys-N-derived HeLa peptides.

A. 20 dia-PASEF scan method used for MS2-centric acquisitions for tryptic HeLa peptides. It consists of one MS1 scan followed by 20 dia-PASEF scans with variable m/z isolation widths and two ion mobility windows in one cycle (~2.1 seconds).

B. 16 dia-PASEF scan method used for MS1-centric acquisitions for tryptic HeLa peptides. This method consists of 16 dia-PASEF and four MS1 scans, with each MS1 scan followed by 4 dia-PASEF scans. Similar to the 20 dia-PASEF scan method (A), the cycle time is about two seconds.

C. 20 dia-PASEF scan method was used for the acquisition of Lys-N-derived HeLa peptides. The method was optimized to specifically cover both doubly- and triply-charged precursors. One cycle time is about 2.1 seconds.

D. Eight dia-PASEF scan method for ultra-high sensitivity measurement of single-cell equivalents and single cells using the ‘Whisper40 SPD’ gradient in combination with an Aurora Elite LC

column (IonOpticks). The acquisition scheme is plotted on top of a kernel density distribution of the precursors from a representative tryptic 48 high-pH fraction HeLa library (Skowronek *et al*, 2022b).

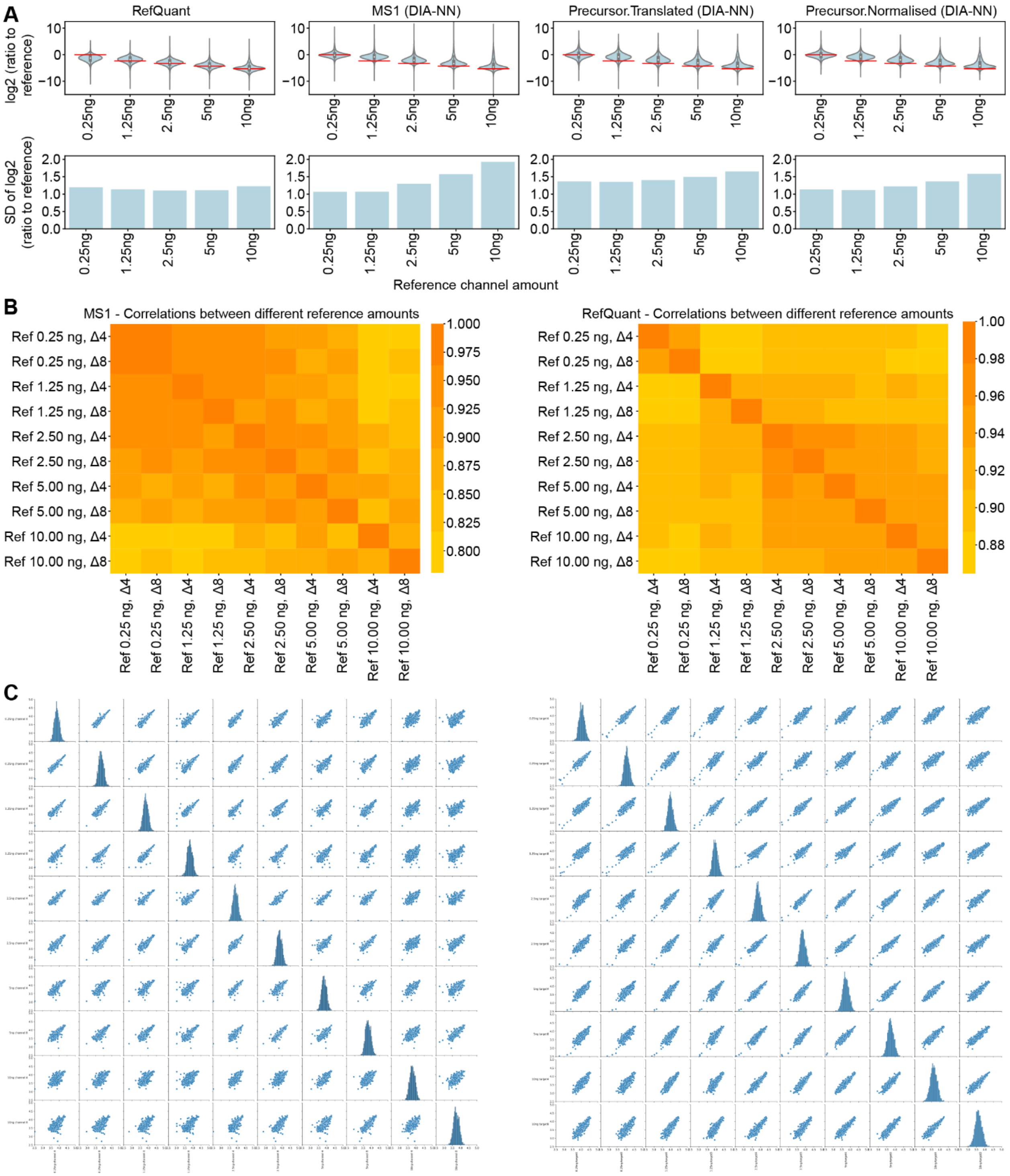

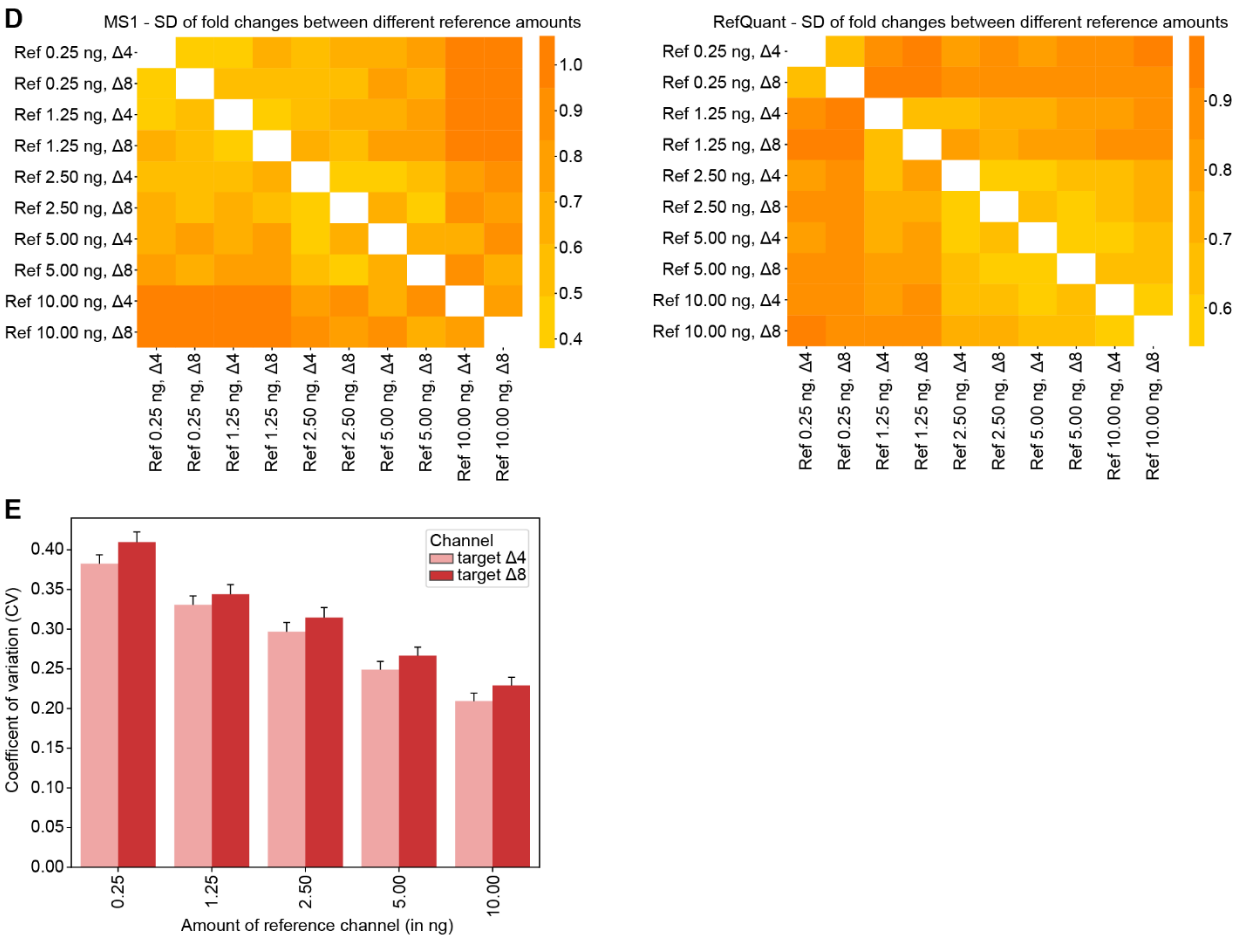

Appendix Figure S4 - Quantitative accuracy and reproducibly of target channels in dependence of the reference channel amount using the scReference dataset.

A. Comparison of the observed ratios to reference to the known ground truth ratio (scReference dataset). Top: distributions as violin plots, bottom: distribution width quantified via the standard deviation. Dependence on amount of reference channel is observed most strongly for MS1 data and RefQuant eliminates most biases. RefQuant under-estimates the ratios in the 0.25ng case, as the model assumptions of RefQuant rely on having a more abundant reference channel. It is not applicable to non-reference channel settings.

B and C. Quantification accuracy in Pearson correlation coefficients (B) or quantity comparisons (C) between single cell equivalents in target channels (Δ4 and Δ8) dependent on different reference channel amounts (0.25ng to 10ng) using the scReference dataset on unmodified MS1 quantities (left) and RefQuant quantities (right). Correlations are improved by using RefQuant and effects of reference channel amounts are mitigated.

D. As in B, but using the standard deviation of the fold changes between precursors. Quantification accuracy yields similar results.

E. Decreasing CV values within the data of single cell equivalents as the amount in the reference channel is increased from 250 pg to 10 ng (by using the scReference dataset, (technical replicates, n=5). Bars and errors depict mean ± standard deviation.

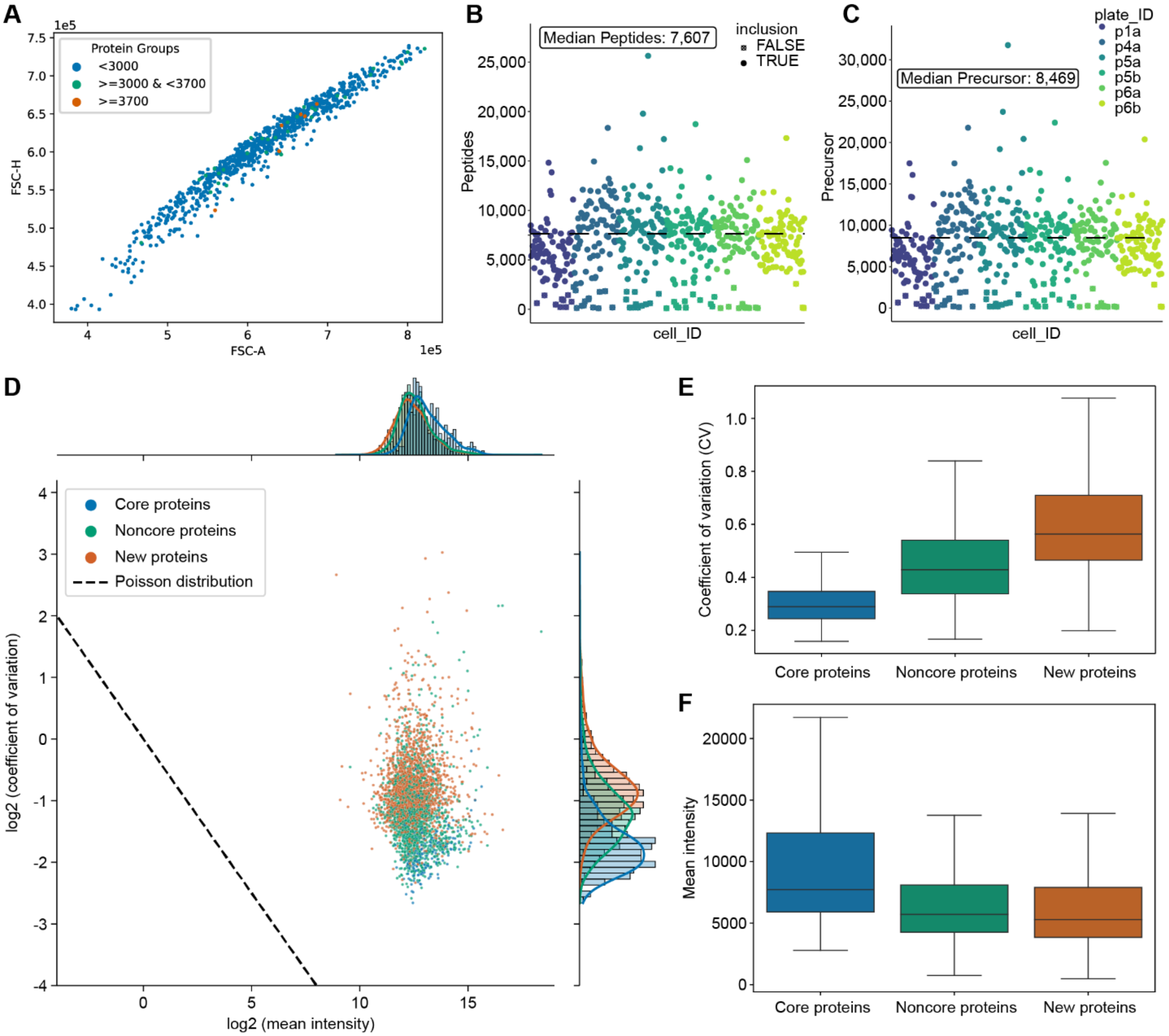

Appendix Figure S5 - mDIA shows that the concept of a stable proteome is still valid at higher proteomic depth in single cells.

A. FACS plot of 476 single HeLa cells of forward scatter height (FSC-H) versus FSC-A (area). The data suggest absence of cell doublets and no correlation of FACS signature with protein identifications.

B. and C. Peptides (B) and precursor (C) identifications of single cells across different plates and runs.

D. Coefficients of variation of single-cell mDIA protein expression plotted against the mean intensity of each protein. ‘Core proteins’ are labeled in blue, ‘noncore proteins’ in green and additionally identified and quantified proteins compared to our previous single-cell publication in orange (Brunner *et al*, 2022).

E. Distribution of coefficients of variation across the mDIA single-cell dataset for ‘core’ (blue), ‘noncore’ (green) and ‘new’ (orange) proteins (biological replicates, n=476). The box depicts the interquartile range with the central band representing the median value of the dataset. The whiskers represent the furthest datapoint within 1.5 times the interquartile range (IQR).

F. Distribution of the mean MS intensity across the mDIA single-cell dataset for ‘core’ (blue), ‘noncore’ (green) and ‘new’ (orange) proteins (biological replicates, n=476). The box depicts the interquartile range with the central band representing the median value of the dataset. The whiskers represent the furthest datapoint within 1.5 times the interquartile range (IQR).

Appendix Tables

Appendix Table S1 - Variable window sizes for the Orbitrap DIA method used to measure dimethyl 3-plex tryptic BSA.

Window	m/z	z	RT Time (min)	Window (min)	Isolation Window (m/z)
1	368.5	2	15	30	137
2	458.5	2	15	30	45
3	497.5	2	15	30	35
4	529.5	2	15	30	31
5	558.5	2	15	30	29
6	587.0	2	15	30	30
7	615.5	2	15	30	29
8	642.5	2	15	30	27
9	669.5	2	15	30	29
10	699.0	2	15	30	32
11	732.0	2	15	30	36
12	771.0	2	15	30	44
13	818.5	2	15	30	53
14	879.5	2	15	30	71
15	969.0	2	15	30	110
16	1334.0	2	15	30	622

Appendix Table S2 - Variable window sizes for the Orbitrap DIA method used to measure dimethyl 3-plex tryptic HeLa.

Window	m/z	z	RT Time (min)	Window (min)	Isolation Window (m/z)
1	339.0	3	37.5	75	78
2	391.0	3	37.5	75	28
3	414.5	3	37.5	75	21
4	432.5	3	37.5	75	17
5	448.0	3	37.5	75	16
6	462.0	3	37.5	75	14
7	474.5	3	37.5	75	13
8	486.0	3	37.5	75	12
9	497.0	3	37.5	75	12
10	507.5	3	37.5	75	11
11	517.5	3	37.5	75	11
12	527.5	3	37.5	75	11
13	537.5	3	37.5	75	11
14	547.0	3	37.5	75	10
15	556.0	3	37.5	75	10
16	565.5	3	37.5	75	11
17	575.0	3	37.5	75	10
18	584.0	3	37.5	75	10
19	593.5	3	37.5	75	11
20	603.5	3	37.5	75	11
21	613.0	3	37.5	75	10
22	622.5	3	37.5	75	11
23	632.5	3	37.5	75	11
24	642.5	3	37.5	75	11
25	652.5	3	37.5	75	11
26	663.0	3	37.5	75	12
27	674.0	3	37.5	75	12
28	685.0	3	37.5	75	12
29	696.0	3	37.5	75	12
30	707.5	3	37.5	75	13
31	720.0	3	37.5	75	14
32	733.0	3	37.5	75	14
33	746.5	3	37.5	75	15
34	761.5	3	37.5	75	17
35	777.5	3	37.5	75	17
36	795.0	3	37.5	75	20
37	814.0	3	37.5	75	20
38	834.5	3	37.5	75	23
39	859.0	3	37.5	75	28
40	887.5	3	37.5	75	31
41	921.5	3	37.5	75	39
42	966.0	3	37.5	75	52
43	1032.0	3	37.5	75	82
44	1358.5	3	37.5	75	573

Appendix Table S3 - 20S MS2-centric dia-PASEF method optimized for tryptic digests (See Appendix Fig S3A).

MS Type	Cycle Id	Start IM [1/K0]	End IM [1/K0]	Start Mass [m/z]	End Mass [m/z]	CE [eV]
MS1	0	-	-	-	-	-
dia-PASEF	1	0.85	1.30	639.03	651.33	-
dia-PASEF	1	0.70	0.85	300.85	377.86	-
dia-PASEF	2	0.87	1.30	651.33	664.31	-
dia-PASEF	2	0.70	0.87	377.86	412.59	-
dia-PASEF	3	0.89	1.30	664.31	676.84	-
dia-PASEF	3	0.70	0.89	412.59	436.90	-
dia-PASEF	4	0.90	1.30	676.84	691.83	-
dia-PASEF	4	0.70	0.90	436.90	453.77	-
dia-PASEF	5	0.91	1.30	691.83	705.41	-
dia-PASEF	5	0.70	0.91	453.77	468.95	-
dia-PASEF	6	0.92	1.30	705.41	719.88	-
dia-PASEF	6	0.70	0.92	468.95	481.80	-
dia-PASEF	7	0.93	1.30	719.88	734.38	-
dia-PASEF	7	0.70	0.93	481.80	494.75	-
dia-PASEF	8	0.94	1.30	734.38	749.91	-
dia-PASEF	8	0.70	0.94	494.75	506.29	-
dia-PASEF	9	0.94	1.30	749.91	766.41	-
dia-PASEF	9	0.70	0.94	506.29	517.76	-
dia-PASEF	10	0.95	1.30	766.41	784.36	-
dia-PASEF	10	0.70	0.95	517.76	529.28	-
dia-PASEF	11	0.96	1.30	784.36	801.41	-
dia-PASEF	11	0.70	0.96	529.28	540.30	-
dia-PASEF	12	0.97	1.30	801.41	821.07	-
dia-PASEF	12	0.70	0.97	540.30	550.97	-
dia-PASEF	13	0.98	1.30	821.07	839.74	-
dia-PASEF	13	0.70	0.98	550.97	561.30	-
dia-PASEF	14	0.99	1.30	839.74	860.42	-
dia-PASEF	14	0.70	0.99	561.30	572.36	-
dia-PASEF	15	1.00	1.30	860.42	884.43	-
dia-PASEF	15	0.70	1.00	572.36	583.31	-
dia-PASEF	16	1.02	1.30	884.43	914.01	-
dia-PASEF	16	0.70	1.02	583.31	593.86	-
dia-PASEF	17	1.03	1.30	914.01	948.93	-
dia-PASEF	17	0.70	1.03	593.86	604.85	-
dia-PASEF	18	1.05	1.30	948.93	991.54	-
dia-PASEF	18	0.70	1.05	604.85	615.73	-
dia-PASEF	19	1.08	1.30	991.54	1057.58	-
dia-PASEF	19	0.70	1.08	615.73	627.36	-
dia-PASEF	20	1.13	1.30	1057.58	1198.62	-
dia-PASEF	20	0.70	1.13	627.36	639.03	-

Appendix Table S4 - 16S4MS1 MS1-centric dia-PASEF method optimized for tryptic digests (See Appendix Fig S3B).

MS Type	Cycle Id	Start IM [1/K0]	End IM [1/K0]	Start Mass [m/z]	End Mass [m/z]	CE [eV]
MS1	0	-	-	-	-	-
dia-PASEF	1	0.85	1.30	639.33	654.83	-
dia-PASEF	1	0.70	0.85	300.85	387.24	-
dia-PASEF	2	0.88	1.30	654.83	671.35	-
dia-PASEF	2	0.70	0.88	387.24	425.24	-
dia-PASEF	3	0.90	1.30	671.35	688.34	-
dia-PASEF	3	0.70	0.90	425.24	450.27	-
dia-PASEF	4	0.91	1.30	688.34	705.86	-
dia-PASEF	4	0.70	0.91	450.27	469.25	-
MS1	5	-	-	-	-	-
dia-PASEF	6	0.92	1.30	705.86	723.36	-
dia-PASEF	6	0.70	0.92	469.25	485.25	-
dia-PASEF	7	0.93	1.30	723.36	742.94	-
dia-PASEF	7	0.70	0.93	485.25	500.91	-
dia-PASEF	8	0.94	1.30	742.94	762.46	-
dia-PASEF	8	0.70	0.94	500.91	515.27	-
dia-PASEF	9	0.96	1.30	762.46	784.91	-
dia-PASEF	9	0.70	0.96	515.27	529.29	-
MS1	10	-	-	-	-	-
dia-PASEF	11	0.97	1.30	784.91	807.43	-
dia-PASEF	11	0.70	0.97	529.29	543.30	-
dia-PASEF	12	0.98	1.30	807.43	831.77	-
dia-PASEF	12	0.70	0.98	543.30	556.31	-
dia-PASEF	13	0.99	1.30	831.77	855.44	-
dia-PASEF	13	0.70	0.99	556.31	569.79	-
dia-PASEF	14	1.01	1.30	855.44	884.96	-
dia-PASEF	14	0.70	1.01	569.79	583.33	-
MS1	15	-	-	-	-	-
dia-PASEF	16	1.02	1.30	884.96	922.39	-
dia-PASEF	16	0.70	1.02	583.33	596.34	-
dia-PASEF	17	1.04	1.30	922.39	969.99	-
dia-PASEF	17	0.70	1.04	596.34	610.34	-
dia-PASEF	18	1.07	1.30	969.99	1042.54	-
dia-PASEF	18	0.70	1.07	610.34	624.35	-
dia-PASEF	19	1.14	1.30	1042.54	1198.62	-
dia-PASEF	19	0.70	1.14	624.35	639.33	-

Appendix Table S5 - Overview of important parameters used in the MS2-optimized acquisition schemes for both Orbitrap and timsTOF and MS1-optimized for timsTOF.

	Orbitrap	timsTOF	
Acquisition scheme	MS2-optimized	MS1-optimized	MS2-optimized
Number of MS1 scans	1	4	1
Number of MS2 scans	44	16	20
MS1 Resolution	120,000	40,000	40,000
MS2 Resolution	30,000	40,000	40,000
Cycle Time (s)	~3.2	~2.12	~2.23
Data points per peak	4	5	5

Appendix Table S6 - 20S MS2-centric dia-PASEF method optimized for Lys-N HeLa digest (See Appendix Fig S3C).

MS Type	Cycle Id	Start IM [1/K0]	End IM [1/K0]	Start Mass [m/z]	End Mass [m/z]	CE [eV]
MS1	0	-	-	-	-	-
dia-PASEF	1	0.87	1.30	710.89	724.34	-
dia-PASEF	1	0.70	0.87	314.86	420.92	-
dia-PASEF	2	0.88	1.30	724.34	736.91	-
dia-PASEF	2	0.70	0.88	420.92	460.56	-
dia-PASEF	3	0.90	1.30	736.91	749.89	-
dia-PASEF	3	0.70	0.90	460.56	483.28	-
dia-PASEF	4	0.91	1.30	749.89	762.89	-
dia-PASEF	4	0.70	0.91	483.28	504.93	-
dia-PASEF	5	0.92	1.30	762.89	776.92	-
dia-PASEF	5	0.70	0.92	504.93	519.82	-
dia-PASEF	6	0.93	1.30	776.92	791.44	-
dia-PASEF	6	0.70	0.93	519.82	536.54	-
dia-PASEF	7	0.94	1.30	791.44	804.38	-
dia-PASEF	7	0.70	0.94	536.54	549.30	-
dia-PASEF	8	0.95	1.30	804.38	819.38	-
dia-PASEF	8	0.70	0.95	549.30	562.30	-
dia-PASEF	9	0.96	1.30	819.38	833.37	-
dia-PASEF	9	0.70	0.96	562.30	574.31	-
dia-PASEF	10	0.97	1.30	833.37	849.49	-
dia-PASEF	10	0.70	0.97	574.31	586.32	-
dia-PASEF	11	0.98	1.30	849.49	866.48	-
dia-PASEF	11	0.70	0.98	586.32	597.31	-
dia-PASEF	12	0.99	1.30	866.48	884.48	-
dia-PASEF	12	0.70	0.99	597.31	609.33	-

dia-PASEF	13	1.00	1.30	884.48	905.43	-
dia-PASEF	13	0.70	1.00	609.33	621.98	-
dia-PASEF	14	1.01	1.30	905.43	929.94	-
dia-PASEF	14	0.70	1.01	621.98	633.38	-
dia-PASEF	15	1.02	1.30	929.94	951.45	-
dia-PASEF	15	0.70	1.02	633.38	644.36	-
dia-PASEF	16	1.03	1.30	951.45	980.48	-
dia-PASEF	16	0.70	1.03	644.36	655.83	-
dia-PASEF	17	1.04	1.30	980.48	1015.17	-
dia-PASEF	17	0.70	1.04	655.83	668.35	-
dia-PASEF	18	1.06	1.30	1015.17	1059.96	-
dia-PASEF	18	0.70	1.06	668.35	681.85	-
dia-PASEF	19	1.07	1.30	1059.96	1111.58	-
dia-PASEF	19	0.70	1.07	681.85	695.86	-
dia-PASEF	20	1.10	1.30	1111.58	1199.65	-
dia-PASEF	20	0.70	1.10	695.86	710.89	-

Appendix Table S7 - Eight dia-PASEF scan method optimized for tryptic HeLa digest and ultra-high sensitivity experiments (See Appendix Fig S3D).

MS Type	Cycle Id	Start IM [1/K0]	End IM [1/K0]	Start Mass [m/z]	End Mass [m/z]	CE [eV]
MS1	0	-	-	-	-	-
dia-PASEF	1	0.83	1.30	636.78	667.83	-
dia-PASEF	1	0.70	0.83	300.49	421.89	-
dia-PASEF	2	0.88	1.30	667.83	700.37	-
dia-PASEF	2	0.70	0.88	421.89	460.78	-
dia-PASEF	3	0.90	1.30	700.37	737.37	-
dia-PASEF	3	0.70	0.90	460.78	493.24	-
dia-PASEF	4	0.92	1.30	737.37	779.36	-
dia-PASEF	4	0.70	0.92	493.24	522.55	-
dia-PASEF	5	0.94	1.30	779.36	828.43	-
dia-PASEF	5	0.70	0.94	522.55	550.30	-
dia-PASEF	6	0.96	1.30	828.43	889.46	-
dia-PASEF	6	0.70	0.96	550.30	577.82	-
dia-PASEF	7	1.00	1.30	889.46	976.47	-
dia-PASEF	7	0.70	1.00	577.82	606.34	-
dia-PASEF	8	1.06	1.30	976.47	1199.61	-
dia-PASEF	8	0.70	1.06	606.34	636.78	-

Appendix Table S8 - Twelve dia-PASEF scan method for mDIA-DVP on Whisper20 gradient.

MS Type	Cycle Id	Start IM [1/K0]	End IM [1/K0]	Start Mass [m/z]	End Mass [m/z]	CE [eV]
MS1	0	-	-	-	-	-
dia-PASEF	1	0.84	1.30	674.37	695.36	-
dia-PASEF	1	0.70	0.84	350.16	433.55	-
dia-PASEF	2	0.87	1.30	695.36	718.34	-
dia-PASEF	2	0.70	0.87	433.55	467.54	-
dia-PASEF	3	0.89	1.30	718.34	741.90	-
dia-PASEF	3	0.70	0.89	467.54	493.77	-
dia-PASEF	4	0.90	1.30	741.90	767.41	-
dia-PASEF	4	0.70	0.90	493.77	516.58	-
dia-PASEF	5	0.92	1.30	767.41	794.94	-
dia-PASEF	5	0.70	0.92	516.58	537.29	-
dia-PASEF	6	0.94	1.30	794.92	825.37	-
dia-PASEF	6	0.70	0.94	537.29	557.28	-
dia-PASEF	7	0.95	1.30	825.37	857.42	-
dia-PASEF	7	0.70	0.95	557.28	575.97	-
dia-PASEF	8	0.97	1.30	857.42	893.92	-
dia-PASEF	8	0.70	0.97	575.97	594.82	-
dia-PASEF	9	0.99	1.30	893.92	937.18	-
dia-PASEF	9	0.70	0.99	594.82	614.27	-
dia-PASEF	10	1.01	1.30	937.18	991.44	-
dia-PASEF	10	0.70	1.01	614.27	633.82	-
dia-PASEF	11	1.04	1.30	991.44	1065.55	-
dia-PASEF	11	0.70	1.04	633.82	653.82	-
dia-PASEF	12	1.09	1.30	1065.55	1199.94	-
dia-PASEF	12	0.70	1.09	653.82	674.37	-

Appendix Texts

Appendix Text S1 - Fundamental considerations about the noise in target and reference for ratio-based quantification

We investigated to what extent, if any, the ratio between reference and target channels would contribute to variance in the experimental values. If a given peptide had a 50% error in its estimated signal in a 40:1 reference to target ratio, this would manifest in a 20.5-fold ratio in a first run. In a second run – given that MS signals are very reproducible – we would then expect a ratio of 20 or perhaps 19.2. From this it follows that - concerning the noise - the variance of the ratio estimation over runs is not determined by magnitude of the ratio. Following this reasoning, the overall variance of the log2 ratios ${\sigma^2}_{ratio}$ is ${\sigma^2}_{ratio} = {\sigma^2}_{target} + {\sigma^2}_{reference}$, with ${\sigma^2}_{target}$ denoting the variance of the target channel and ${\sigma^2}_{reference}$ denoting the variance of the reference channel. As the higher abundances of the reference channel stabilizes its signal, we conclude that the overall variation of the ratios will decrease with higher reference proteome amounts. We also demonstrated our assumption in the target channel by using different amounts in the reference channel comparing the CVs of the single-cell equivalents in the target channel (scReference dataset), as the CVs in the target channel decrease with higher reference channel amounts (Appendix Fig S4E).
