# Supplementary material for: Robust dimethyl‐based multiplex‐DIA doubles single‐cell proteome depth via a reference channel
Source: Mol Syst Biol. 2023 Aug 21;19(9):e11503. doi: 10.15252/msb.202211503 (PMC10495816; doi:10.15252/msb.202211503)
Supplement: Supplementary file 2 — Expanded View Figures PDF [file MSB-19-e11503-s002.pdf]

Expanded View Figures

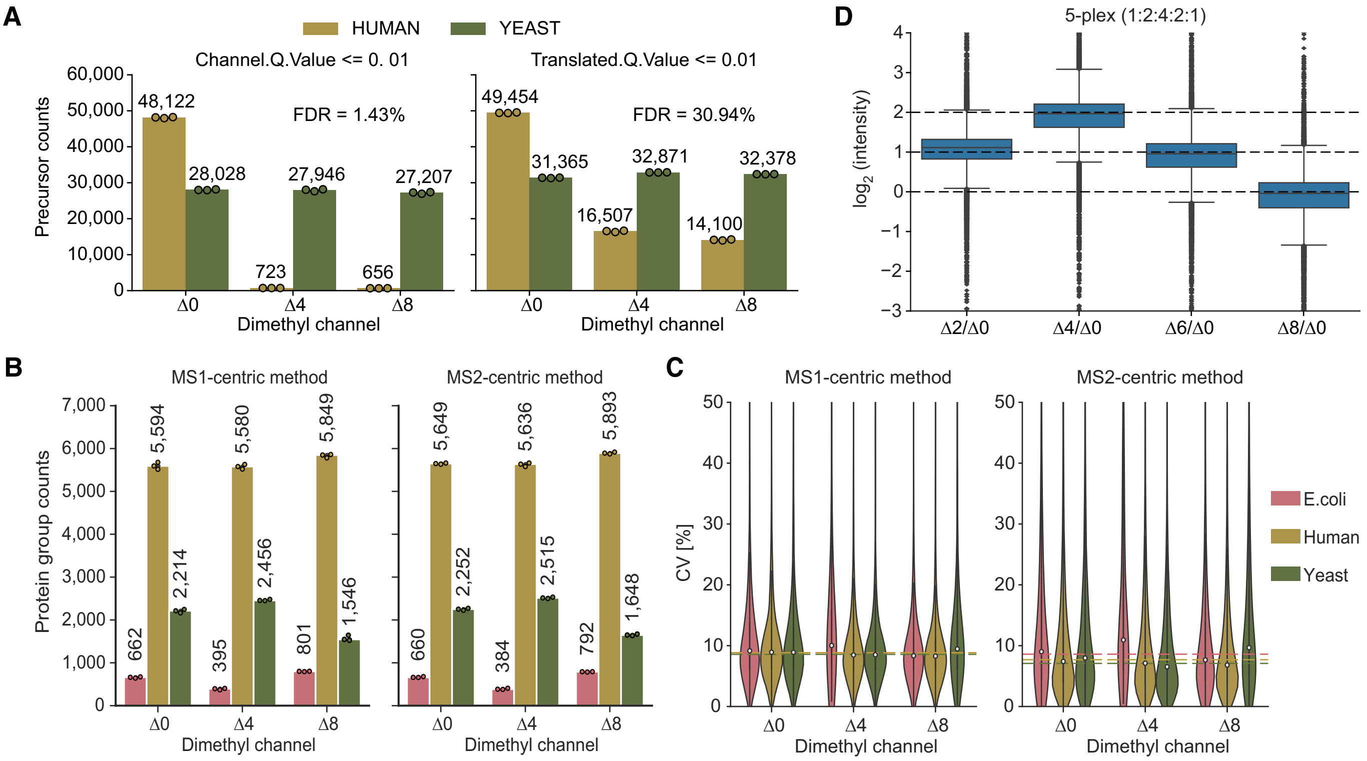

Figure EV1. Identification rates and coefficient of variation (CVs) in the mixed species experiment.

A Determination of the empirical FDR based on filter cut-offs of 'Channel.Q.Value' ≤ 0.01 (left panel) and 'Translated.Q.Value' ≤ 0.01 (right panel). All channels contain 100 ng of labeled tryptic yeast peptides. Additionally, channel Δ0 contains 100 ng labeled tryptic HeLa peptides (human) (technical replicates, $n = 3$).

B Tryptic peptides from E. coli, yeast and human were combined at defined ratios before dimethyl labeling. Side-by-side comparison of the number of quantified protein groups for the individual species with MS1- and the MS2-centric methods. 100 ng of peptides were injected per channel (technical replicates, $n = 3$).

C Coefficients of variation (CVs, %) of protein groups shown in (A). Median CVs for three technical replicates ($n = 3$) are shown as dashed lines.

D Quantification accuracy assessment of 5-plex by Lys-N of labeled HeLa peptides in the respective channel (Δ0, Δ2, Δ4, Δ6, Δ8) in a ratio of 1:2:4:2:1 (technical replicates, $n = 3$). Protein group ratios are plotted as boxplots with expected ratios as dashed lines normalized to the Δ0 channel. The box shows the interquartile range with the central band representing the median value of the dataset. The whiskers represent the furthest datapoint within 1.5 times the interquartile range (IQR).

 © 2023 The Authors

Dataset name	Reference channel (Δ0)	Target channels (Δ4 and Δ8)
scReference	0.25ng to 10ng	Single cell equivalent
scBenchmark	10ng	Single cell equivalent
scQuant	10ng	0.0625ng to 2ng
scDecoy	10ng	Empty or<br>single cell equivalent

Figure EV2. Overview of datasets used for evaluation of the reference channel.

Figure EV3. Evaluation of 'Translated.Q.Value' and 'Channel.Q.Value' on identifications in empty target channels and quantification in single-cell equivalents.

A Dilution series of HeLa peptides to define protein identifications in mDIA workflow, in which the linear increase with input amount levels off at about 10 ng (technical replicates, $n = 3$).

B Precursor identifications of empty target channels at 1% 'Translated.Q.Value' revealed more than 1% false positives (left). Precursor (middle) and protein (right) identifications of empty target channels at 15% Channel.Q.Value showed a FDR lower than 1% (scDecoy dataset).

C, D Count-based FDR estimation on precursor (C) and protein level (D) by dividing two quantities, namely the maximum number of identified precursors or proteins in four empty target channel runs by the minimum number of identified precursors or proteins in a total of four target channels (target channel Δ4 and Δ8) which contain single-cell equivalent amounts (scDecoy). The count-based FDR reveals a 'Channel.Q.Value' filter of 0.45 for precursors and 0.17 (dashed line) for proteins at 1%. For comparison, the used 'Channel.Q.Value' cutoff of 0.15 is shown as well (thick dashed line).

E Precursor count-based FDR derived from empty target channels with mouse liver tissue samples at 15% Channel.Q.Value is much below 1%.

F Impact of the reference channel on identification in the target channels of single-cell equivalents. We compare single-cell equivalents in the target channels with (10 ng Δ0-labeled HeLa) and without reference channel (10 ng unlabeled HeLa) (technical replicates, $n = 6$). Bars and errors depict median ± standard deviation.

G Quantification evaluation of 'Channel.Q.Value' filter. Ratios of reference to target channels (top), channel 4 (middle) and 8 (bottom) by RefQuant (Fig 5) are shown for arginine and lysine precursor at a given filter of 'Channel.Q.Value' (technical replicates, $n = 5$). At all filtering steps, the modes of the distributions are close to the expected ratio (dashed line) with a systematic skew towards lower ratios, which is more pronounced for the arginine precursors. The skews in the distributions are decreased with increased filtering stringency in a gradual manner. The mean quantification ratios are as expected, whereas they start to deviate at 0.2.

© 2023 The Authors

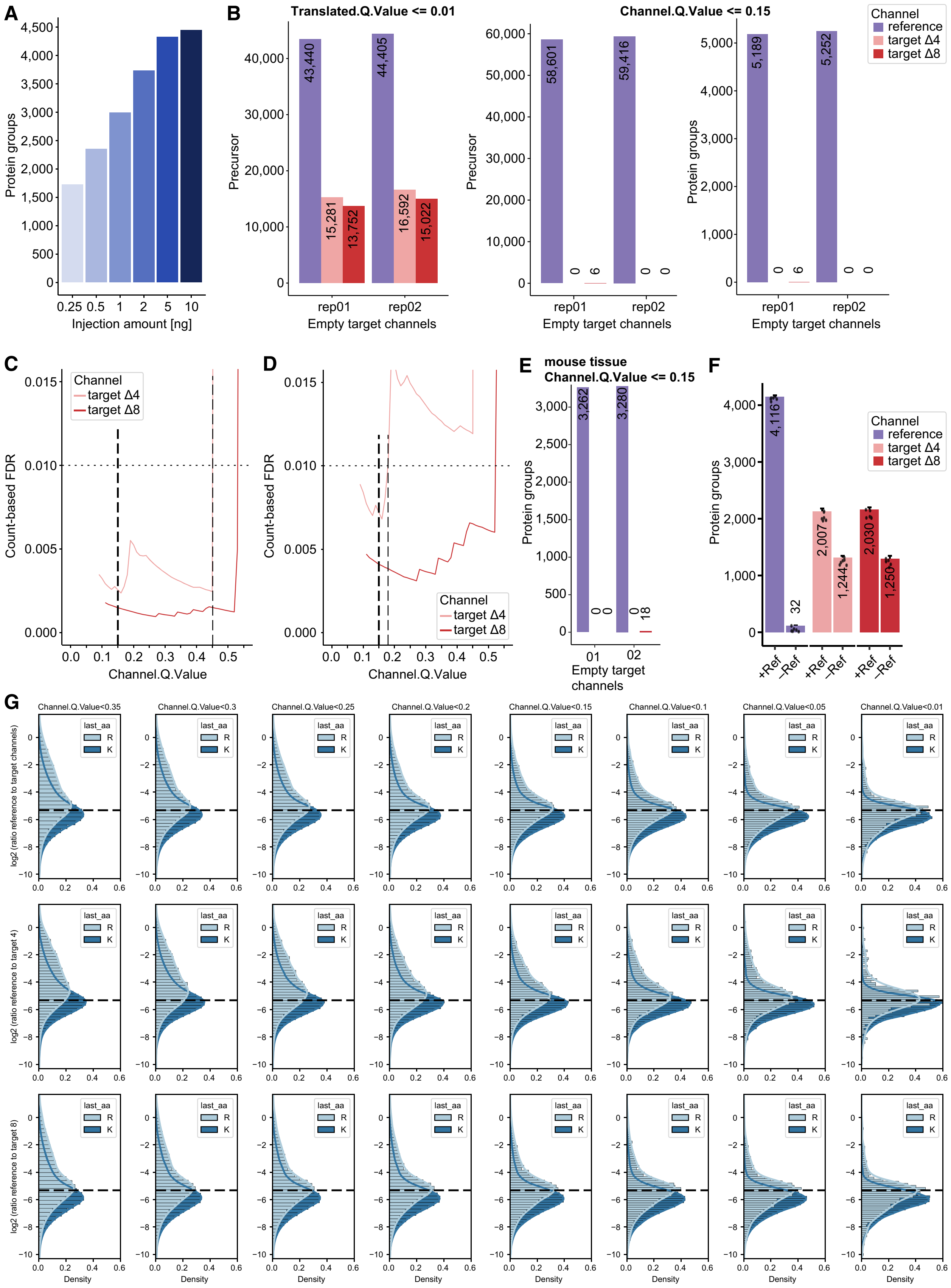

Figure EV3.

 © 2023 The Authors

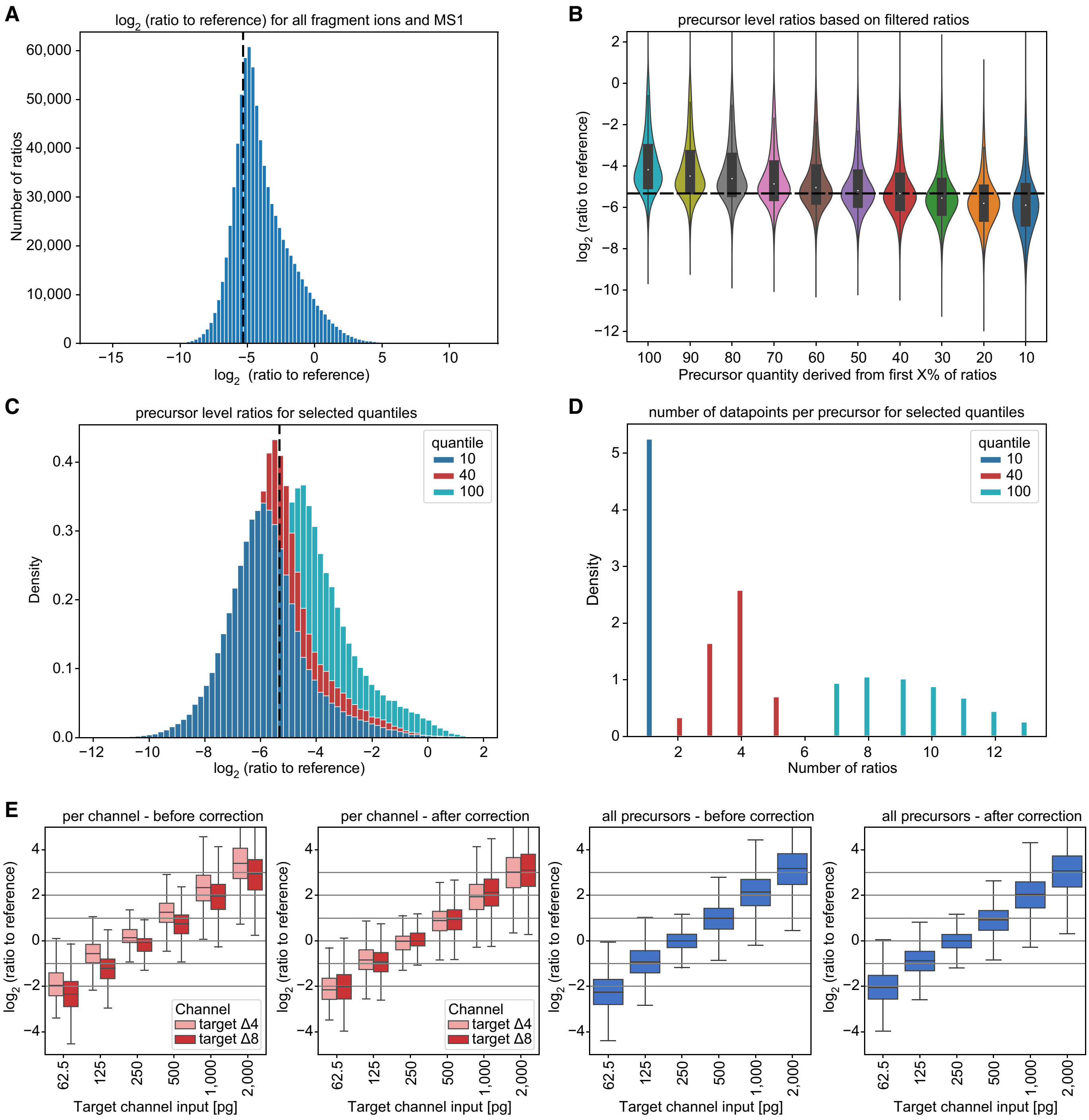
A
log2 (ratio to reference) for all fragment ions and MS1
Number of ratios
log2 (ratio to reference)
B
precursor level ratios based on filtered ratios
log2 (ratio to reference)
Precursor quantity derived from first X% of ratios
C
precursor level ratios for selected quantiles
quantile
10
40
100
Density
log2 (ratio to reference)
D
number of datapoints per precursor for selected quantiles
quantile
10
40
100
Density
Number of ratios
E
per channel - before correction
per channel - after correction
all precursors - before correction
all precursors - after correction
log2 (ratio to reference)
Channel
target Δ4
target Δ8
Target channel input [pg]

Figure EV4.

◀ **Figure EV4. Ratios to reference channel evaluation and channel correction comparison.**

A Ratios to the reference for all available ions (fragment ions and MS1 peaks, scBenchmark dataset), revealing a distribution that has a mode proximate to the expected ground truth (dashed line). The distribution is asymmetric with a skew towards less extreme ratios. A potential explanation for this skew is that noise or interferences are dominant for a fraction of the ratios (a ratio of 0 might be a 'noise vs. noise' comparison).

B An approach to mitigate this asymmetry is to filter out some ions before estimating the precursor level ratio. For this, the ratios of a precursor are sorted ascending and only the first ratios are retained (up to the 'X value' indicated) using the scBenchmark dataset. The precursor ratio is estimated by taking the mean of the remaining ratios. We see that retaining 40% of the ratios matches the ground truth well and is more symmetric (technical replicates, $n = 5$). The violin plot shows the distribution of the data while the box depicts the interquartile range with the central band representing the median value of the dataset. The whiskers represent the furthest datapoint within 1.5 times the interquartile range (IQR).

C A more detailed comparison of the distributions when taking 10%, or 40% of ratios as compared to all ratios.

D Number of available ratios per precursor after filtering. We see that in general between 7 and 14 ratios are available, which reduces to 2–5 when taking the 40% quantile.

E Channel correction comparison in scQuant dataset in comparison of all precursor and target channel (technical replicates, $n = 5$). Ratio of reference channel to target channel was calculated using RefQuant. All $\log_2$ ratios were normalized to the single-cell equivalent sample in the target channel (250 pg). A basic median normalization between the channels was applied. The violin plot shows the distribution of the data while the box depicts the interquartile range with the central band representing the median value of the dataset. The whiskers represent the furthest datapoint within 1.5 times the interquartile range (IQR).

 © 2023 The Authors
