# Supplementary material for: Robust dimethyl‐based multiplex‐DIA doubles single‐cell proteome depth via a reference channel
Source: Mol Syst Biol. 2023 Aug 21;19(9):e11503. doi: 10.15252/msb.202211503 (PMC10495816; doi:10.15252/msb.202211503)
Supplement: Supplementary file 6 [file MSB-19-e11503-s006.pdf]

Robust dimethyl-based multiplex-DIA doubles single-cell proteome depth via a reference channel

Marvin Thielert, Ericka Itang, Constantin Ammar, Florian Rosenberger, Isabell Bludau, Lisa Schweizer, Thierry Nordmann, Patricia Skowronek, Maria Wahle, Wen-Feng Zeng, Xie-Xuan Zhou, Andreas-David Brunner, Sabrina Richter, Mitchell Levesque, Fabian Theis, Martin Steger, and Matthias Mann
DOI: 10.15252/msb.202211503

Corresponding author(s): Matthias Mann (mmann@biochem.mpg.de)

Review Timeline:

Submission Date:	9th Dec 22
Editorial Decision:	19th Jan 23
Revision Received:	29th May 23
Editorial Decision:	4th Jul 23
Revision Received:	17th Jul 23
Accepted:	25th Jul 23

Editor: Maria Polychronidou

Transaction Report:

(Note: With the exception of the correction of typographical or spelling errors that could be a source of ambiguity, letters and reports are not edited. Depending on transfer agreements, referee reports obtained elsewhere may or may not be included in this compilation. Referee reports are anonymous unless the Referee chooses to sign their reports.)

19th Jan 2023

RE: Manuscript MSB-2022-11503, "Robust dimethyl-based multiplex-DIA doubles single-cell proteome depth via a reference channel"

Dear Matthias,

Thank you again for submitting your work to Molecular Systems Biology. We have now heard back from the three referees who agreed to evaluate your study. As you will see below, the reviewers raise substantial concerns, which unfortunately preclude the publication of the study in its current form.

The reviewers appreciate that the presented workflow seems potentially relevant for single-cell proteomics. However, they point out that as it stands the study seems rather preliminary and they raise significant concerns regarding the validity of several of the reported conclusions. They also mention that the relatively limited biological insights derived from the performed analyses do not provide sufficient support for the value of the approach. Given the rather substantial issues raised, at this point we can unfortunately not offer to publish the study.

Nevertheless, as the reviewers did acknowledge that new methodological developments for single-cell proteomics are relevant for the field, we would not be opposed to considering a substantially revised and extended manuscript, provided that the issues raised by the reviewers can be convincingly addressed. The reviewers provide constructive and detailed suggestions on how the study could be improved. The most essential issues that would need to be addressed include:

- Additional experimental and computational analyses are required to ensure the quality of the presented data and the validity of the derived conclusions. All reviewers provide constructive recommendations in this regard.

- As reviewer #2 and #3 point out, an application demonstrating the potential of the presented methodology to derive new biology would be very important for better supporting the overall methodological advance and broader relevance of the study.

All other issues raised by the reviewers need to be satisfactorily addressed. We recognize that thoroughly addressing the referees' concerns would involve substantial further analyses. If you are considering proceeding with a resubmission, I would be happy to schedule a call or look at a preliminary point by point response delineating how the issues raised can be addressed, so that we can work together on how to move forward.

As you probably understand, we can give no guarantee about the eventual acceptability of the resubmitted work. If you do decide to follow this course, then we would ask you to enclose with your resubmission a point-by-point response to the points raised in the present review.

I am sorry that the review of your work did not result in a more favorable outcome on this occasion, but I hope that you will not be discouraged from sending your work to Molecular Systems Biology in the future. In any case, thank you for the opportunity to examine this work.

Kind regards,

Maria

Maria Polychronidou, PhD
Senior Editor
Molecular Systems Biology

Reviewer #1:

Thielert et al present an exciting technological development for multiplexed DIA, reminiscent of the recently published plexDIA workflow (Derks et al, 2022). Although mDIA is proposed to facilitate multiplexing up to 5 samples, for single-cell proteome profiling, mDIA combines two cells and a reference channel. In contrast to the plexDIA approach, the throughput is lower by 40 cells per day, but the proteome coverage appears to have increased more than two-fold. The authors then leverage their established mDIA method to reiterate biological findings they have made in a previous study (Brunner et al, 2022). The manuscript presents an interesting workflow that enriches the arsenal of DIA and single-cell proteomics enthusiasts alike. However, there are several important issues present in the study, rendering the current state of the manuscript not suitable for

publication and requiring substantial revision of the main text and, likely, additional experiments and/or computational analyses to ensure the quality of data obtained with the established workflow. Overall, the manuscript feels a little rushed, and just to name an example, the figures should have been labeled to make it easier to refer to them correctly while reading the main text. Also, the initial literature overview of previous achievements in the field of single-cell proteomics is rather limited, leaving the less knowledgeable reader with a false impression of the true gains that were made with mDIA compared to existing capabilities and not crediting other labs active in the field sufficiently. With the young state of the field of SCP, it is imperative that we ensure our results can withstand the greatest scrutiny, as we will otherwise never be taken seriously by the more general biologists and life science specialists. All developments are welcome, but need to undergo rigorous evaluations to ensure the quality thereof, which is something I'm not sure the authors fully succeeded with here. Whether this was due to them being under time pressure, or just overly excited to share their work with the community we don't know, but hopefully they can address the main shortcomings below more elaborately, as there is real impact expected from their work once final.

Major concerns

1. The abstract appears somewhat misleading. For example, the authors write that "in single runs of mammalian cells, ..., 7,700 proteins" were quantified per channel. It should be better clarified that this does not entail single cells, and rather refer to "xx ng of complex mammalian lysate". As it stands, the sentence is not very informative. Moreover, the authors state that mDIA quantifies "close to 4,000 proteins in single cells with excellent reproducibility". However, when looking at figure 6, this is entirely not the case (!). The median PG is 2,377 proteins per cell, and it is this number that should be listed here. However, regarding this median number, I will get back to other concerns about this figure in point 10 below.

2. Fig 2D is referenced before 2C, and upon closer inspection, 2C is not referenced at all, despite there being a substantial increase in CV on both OT and TOF with added channels - this needs to be explored in much more depth and claims substantiated! Why was 2C left out of the discussion, when it clearly demonstrates a bias in the measured ratios when extra channels are added? Is this due to false identifications and thus quantification of the wrong signal, or are these interferences that add up to the correct signal, resulting in high variance?

3. The text describing Fig 2E claims that the measured protein ratios largely agree, which when looking at the data, appears to be an overstatement. Measured ratios seem significantly off compared to the expected value in most cases (e.g. yeast comparison d0/d8 and e.coli comparison d4/d8), which together with 2C indicates issues with the quality of quantification. This is not the case in e.g. the plexDIA paper (Derks et al., Fig. 3). The authors should explain these phenomena. Some measurements seem to have ratio compression, while others have even higher ratios.

4. It was nice to see that the authors employed a species mix to asses accuracy, as it is an elegant way to increase proteome complexity and even enable aspects such as FDR calculation etc. Regarding FDR, the authors missed an opportunity where e.g. one channel does not contain e.coli (e.g. d4) and where they could calculate the false transfer rate of e.coli peptides from the other channels onto this channel to assess empirical FDR. Especially in light of mDIA taking a reference channel approach (which in my opinion should be called a booster channel), it is important to assess cross-channel ID transfer and FDR thereof.

5. Regarding Fig 3D, a similar problem emerges as 2C, where the fact that CV values are increasing are not described nor evaluated in the text. Again, is this due to false identifications or interferences? Similarly, as the authors are keen on establishing mDIA as a 5-plex single cell proteomics workflow, they should evaluate accuracy in this labelling context too.

6. On pg. 9, the authors claim that "the different non-isobaric channels in the mDIA workflow are decoupled from each other in terms of quantification". Here I have to disagree, as Figs 2C and 3S clearly show that this is not true, also supported by the poor accuracy tests of Fig 2e. Similarly, on pg. 10 they "conclude that these channels are isolated from each other as expected from the mDIA concept". Again, the data presented in the manuscript does not support this claim!

7. The RefQuant algorithm is an interesting approach, similar to that of the carrier channel used in SCoPE-MS, and represents a major aspect of the impact of this work. The authors decouple precursor identification from quantification by using the reference channel for FDR-controlled identification, and quantifying the signal in the remaining channels by transfer of the peak boundaries of the reference channel. The authors use the Channel.Q.Value in the single-cell channels as a filter to remove transfers to signals that are of similarly low quality as in empty channels. However, it would be important to note at what reference channel amount this parameter was determined, as Fig. EV5 B suggest a crosstalk between reference and target channels. It should be clearly noted that their 40% quantile parameter is empirically determined and results could change with changes in both the overall LC-MS setup used, and the sample investigated. Although it is reassuring that the number of protein quantifications levels off with increasing reference channel amount, there will likely still be interferences that add up to peaks that pass the quality threshold. This could be investigated by quantifying common precursors in the single-cell channels across the different reference inputs (Fig.4C). If present, the extent of the added signal on top of the precursor signal from the reference channel would quantify the contribution of the reference channel, which likely leads to a ratio compression effect. These interferences are likely specific to the channel used and, as it seems from Fig. EV5E, also depend on the last AA in the precursor. It would be important to investigate these interferences further and potentially propose a solution besides qualitative data filtering.

8. Regarding Fig 5C, how did the authors come to roughly 3000 shared precursor counts at 250pg and even in 2000pg? Should the number not increase with higher loads? Moreover, why is it so low compared to nearly 3000 proteins from 250pg in Fig. 4C?

9. I am struggling a bit with their claim that it was as challenging to quantify proteins from 250pg of diluted HeLa digest as it was from single cells, as this contradicts many other observations in the field. Generally, we've seen much higher ID numbers from dilutions than from single cells. To this end, it would be imperative to know what cell sizes were used. The complete lack of FACS data in this manuscript is concerning, as it becomes difficult to connect the number of protein IDs per-cell to cell size. Also, for those few cells where the authors did ID >3,000 proteins, how can we be sure they were still single cells, and not doublets? The .fcs files from the FACS sorting procedure should allow us to resolve these concerns.

10. Related to point 8, only ~2,500 precursors were quantified for the 250pg HeLa dilutions (Fig 5C). Fig 6 indicates that a similar number, but now actual protein groups, were quantified in actual single cells. This is striking as all prior research has demonstrated it to be more difficult to quantify protein groups from actual cells than dilution series (due to sample loss, imperfect digestion etc.). Can the authors explore this observation in more depth and e.g. show the difference in abundance (i.e. TIC or BPC) between the dilution samples and real single cells?

11. Finally, and related to point 1, the authors provide a Source Data Table for Fig 6A which contains 893 columns, indicating that more cells were measured than noted in the main text (476 total) and filtered out before subsequent data analysis. Looking at the included table, it appears that 398 cells in the full dataset delivered below 500 protein groups, of which 372 cells produced less than 100 protein groups. If this is true, this is rather concerning, as it suggests that the experimental workflow might not be entirely robust in general, with a dropout rate of nearly 50%. If one includes these cells in the overall proteins-per-cell calculation, this would decrease the average number of quantified proteins to only ~1450 protein groups. Moreover, as this is only noticeable after MS analysis, this would also lower the throughput of mDIA SCP to only 40, rather than the claimed 80 cells per day. Perhaps the FACS data can provide more insights into this, and this aspect needs to be evaluated in much more detail.

12. A PCA or UMAP plot of the single cell results would be nice to see, to determine if the biological variation due to the cell cycle is captured and no channel (d4/d8) biases are present.

Minor concerns

13. The first half of the introduction covers the field of single-cell proteomics (SCP) in a very shallow manner. The breakthrough studies are barely referenced or wrong references are used (e.g nanoPOTS [Kelly, 2020], where the actual study that established the method is Zhu et al, 2018). The authors should ensure to appropriately credit previous research that propelled the field forward. For example, other studies that have also used 384w plate format with FACS were Liang et al 2021, Specht et al 2021, and Schoof et al, 2021, and deserve mentioning here.

14. Regarding Supplemental Text EV7, I can follow the authors' argumentation until the point where they claim that higher abundances of the reference channel will result in lower variation values. Certainly, the coefficient of variation (CV) will decrease, however, the absolute variation might still increase. The authors should use the data they have to test the consideration made in EV7.

15. I am lacking a bit more detail on the spectral libraries that were used. Were the same libraries of AlphaPeptDeep used for both TOF and OT data? Would it be expected that mass analyser-specific spectral libraries should be used for best performance? Also, I couldn't find the actual libraries in the data submission, something that would be imperative to evaluate their performance independently.

16. I really liked the author's MS1 vs MS2 quant comparison, this was a very nice feature to add and seems relevant for single-cell DIA which is as of yet, still relatively unexplored. However, if the authors are keen on including this in the manuscript, I would like to see more exploration on aspects of e.g. how comparable the OT methods are vs TOF? Was it a fair comparison based on window size/resolution & cycle time? There was not a lot of detail provided for the data acquisition schemes in the methods, which should be improved (Supp. Tables were not much help here either), and could the authors explain how they optimised MS1 vs MS2 methods for OT? On timsTOF, there were 2 clear methods used, but on OT it appears the same method was used for both MS1 and MS2. This is very likely sub-optimal, and possibly explains the observed performance differences between OT and TOF.

17. The idea of a reference/carrier channel in plexDIA has been laid out multiple times already which should be attributed in the manuscript. https://www.nature.com/articles/s41587-022-01389-w, https://www.biorxiv.org/content/10.1101/2022.11.05.515287v1

18. Budnik et al, 2018 introduced the term "carrier channel" and not "booster channel". This terminology should be used correctly when directly citing the original paper.

19. On pg. 8, the authors claim that the derivatization reaction does not complicate the workflow, but if on-tip labelling is used for

single cells, then how are labeled peptides pooled onto their final EvoTip? I would expect this adds an additional elution step?

20. It would be nice to see some data on the claim of a 2s FWHM peak width

21. In the main text the authors state that the upper 40% quantile is used, but in the method section "The ratios are sorted in ascending order and the first 40% of ratios are retained." This would suggest the lower 40% quantile. We assume that the latter one is the correct one.

Reviewer #2:

This paper by Thielert et al. presents a multiplexed workflow using data-independent acquisition (mDIA) using dimethyl labeling of bulk or single-cell samples in an automated way using a liquid handling system. Bulk analyses of mammalian cells in three-plex of tryptic peptides quantified as many as 7,700 proteins per channel. Use of Lys-N for digestion extension of non-isobaric labeling to five-plex quantification. The use and potential added value of a reference channel in mDIA is demonstrated as well as sample analysis throughput of as high as 80 single cells per day. The biological study described extends the previously reported concept of a stable proteome.

Major Issues:

1. This report by Thielert and the Mann group combines several previously reported methods, some well described elsewhere by this group i.e., Brunner et al. processing and analysis methods for single cell analysis in DIA mode, Slavov lab for non-isobaric labeling of samples for multiplexed DIA of bulk and single cell analyses (Derks et al. NatBiotech 2022) and Wu et al. (Chem Commun 2014) for development and use of a 5-plex version of the dimethyl reagents developed by the Heck lab. The present paper combines all of the above in DIA mode which, while interesting, is not particularly novel.

2. The one aspect of the paper that is potentially novel and of interest is the impact of the use of a reference channel on depth of coverage, reproducibility, quantitative accuracy and ability to link data obtained across large numbers of multiplexed DIA analyses. While the reference channel concept is well known and commonly used in multiplexed DDA, and its potential value clearly described for DIA in the Discussion portion of the Derks et al. paper, the present report goes beyond the Derks et al. report in using a reference channel and showing its value in quality controlling target-channel quantitation. A paper focused on this interesting aspect could be valuable especially in the context of single cells and large sample numbers. However, the demonstration presented is rather simplistic (HeLa cells using a HeLa common reference channel at higher load levels) and does not provide experimental results that explain why the reference channel appears to work to boost numbers and reduce the spectral noise. Could the increase in identification simply be an effect of adsorptive losses during sample preparation/acquisition, and the lack of further increase above 5 ng load for reference channel be due to saturation? The impact of the composition of the reference and the absence of carrier peptides in the presence of target peptides was not evaluated. What if it differed from that of the analyte samples tested? At the presumed heterogenous single cell level, how would this be dealt with?

3. Importantly, there is also no new application of the methods that sheds light on some interesting biology. I view this as essential for a paper in MSB. Instead, the authors extend an already published observation that there is a stable core proteome (at least in HeLa cells). Without a relevant new application (such as that cited in prepublication form from these same authors by XXXXX et al.) of the reference method the paper is more suited to a specialized proteomics journal.

Minor issues identified:
1. The numbers reported in the Derks et al paper for multiplexed bulk cell analyses used 1-hour active gradients on an Orbitrap instrument and quantified ~8,000 proteins in each sample. This should be noted in the text as the numbers reported are very similar to this paper by Thielert.
There is also an entire paragraph on comparable IDs for label-free, single and multiple channel IDs making a point that dimethyl labeling does not impact the overall IDs but that the timsTOF is beneficial for deconvolution of multiplexed spectra in complex samples. While this is certainly true, this also has been reported previously by Derks et al. in great detail for the mTRAQ reagents. Again, at a minimum, noting that this has been reported for effectively a very similar experimental paradigm needs to be part of this paragraph.

2. While it is correct that Derks et al. reported ca. 1000 proteins/cell at single cell level using triplex DIA, they only used a 5 min and a 15 min. effective gradient vs. the longer gradient used here. This needs to be made clear in the text.

3. In the Discussion: "We also explored the idea of a reference channel in single-cell mDIA. Note that this is conceptually different from the booster channel employed in the SCoPE-MS method (Budnik et al, 2018) because the fragments of the mDIA channels are offset from each other and do not contribute to a common low reporter mass." This point is already made very clear in the Derks paper and should be cited.

4. Figure 4 and elsewhere: "Target 4" and "Target 8" are unclear - you mean the target delta 4 and target delta 8 channels -

replace.

5. Abstract - "We demonstrate automated and complete dimethyl labeling of bulk or single-cell samples, without losing proteomic depth." You then go on and state: "In single runs of mammalian cells, a three-plex analysis of tryptic peptides quantified 7,700 proteins per channel." The wording is potentially misleading as readers may assume that the second sentence refers to single cell analyses. Be specific that the single runs are for bulk cells, not single cells.

1. Abstract: you state "...confidently quantifies close to 4,000 proteins in single cells with excellent reproducibility" This reviewer is struggling to see where this claim is supported in the results presented. Figure 4C shows at best 3000 proteins identified while Figure 6 shows a median of ca. 2400 proteins with a few cells having ca. 4000. The median value and the interquartile range should be reported, not results for a few outliers.

Reviewer #3:

Thielert et al.combine several known approaches in mass spectrometry (MS) and liquid chromatography (LC) to present a pipeline with increased sensitivity for single cell proteomics.
They couple multiplex data independent acquisition (mDIA) with dimethyl labeling and either trypsin or LysN digestion for 3plex or 5plex measurement respectively. The availability of multiple channels means that one of these can be designated as a reference channel to improve detection sensitivity, which is important for single cell data. In terms of LC, they use the EvoSep system which allows for low-flow separation with pre-made gradients and very small dead volume, all of which are also beneficial for single-cell analysis. After benchmarking they apply their 3plex approach in an automated fashion to about 500 single cells. They detect a median of about 2400 proteins per cell (about double what they previously reported for a similar sample) and report a throughput of 80 cells/day. They show, as they previously described at lower proteome coverage, that the intercellular variation in protein levels is smaller than the variation in transcript levels.
The study is technically sound and in the interesting area of methods development for single-cell proteomics; tech developments are needed in this area if it is to become as useful and widespread as other single cell omics measurements. Nevertheless, the paper is also very incremental, technical, and likely of interest to a specialized readership implementing or wishing to implement such approaches.
Major points
1. Novelty is overall low. Multiplex DIA applied to single cell proteomics is not in itself new (Budnik et al, 2018 PMID: 30343672), nor is dimethyl labeling. The appropriate references are cited. The minor elements of novelty in this manuscript are:
• The combination of multiplexDIA and dimethyl labeling; the study shows that this combination works, but this is not at all surprising.
• The use of LysN for 5 plex measurements, but this is only shown on test data
• The use of the EvoSep system for the LC; again it is not surprising that this works
• The use of a reference channel in multiplexDIA, see point 2
• The RefQuant analysis approach for quantification

2. Given the multiplex nature of the data acquisition, one channel can be designated as a reference channel. Since more material can be run in this channel, the resulting robust identifications can be used to improve identification in the low-amount single-cell channels. This is conceptually not new, it was previously described in Budnik et al, but its actual implementation is new in this work.

To avoid false positives, the authors recommend an empirical threshold, but they also suggest that this reference approach may be applicable to generic proteomics studies. But at higher reference channel load, the chance of false positives may increase and a more stringent threshold may be required. If the authors wish to suggest that this may be a general approach, they should test the generality of the recommended threshold, or discuss that this "channel-q-value" threshold should be carefully re-examined for each experimental setup.

3. The authors claim that their approach quantitatively detects up to 4000 proteins per cell, this claim is even made in the abstract. But this is true only for a few cells (Fig 6A). The authors are clear in the text and indeed in the figure that the median is about 2400 proteins per cell, and this would be the more appropriate statement in the abstract. Also the statement about quantifying 7,700 proteins per channel, while technically correct, is a bit misleading in the abstract since it is the per-cell number that most readers will be looking for and it would be easy to confuse this.

4. Which element of the described approach contributes most substantially to sensitivity gains? The authors state on page 12 that this is due almost entirely to the reference channel, but it is not quite clear on what this is based since there have been apparently several changes to the pipeline in comparison to their previous work. A clear demonstration of where the improved sensitivity is coming from would benefit readers and potential users.

5. For the single-cell experiment shown in Fig 6A, the authors should report the number of peptides detected as well, and not just the number of proteins.

6. The authors go back and forth between describing a 3plex and a 5plex approach, the reason for this is not clear and makes the manuscript more difficult to read. Why did they not use their 5plex method for the actual single-cell experiment? Also, did they develop a different analytical pipeline for the 5plex approach?

7. The authors argue that their data supports a "stable proteome", based on the fact that inter-cell variability in protein levels is smaller than in transcript levels. This is not entirely new; they have reported it before based on data with half the median number of proteins quantified per cell. The observation also feels a bit incremental, because it is still only a subset of proteins and the data come from only about 500 cells.

Also, while this is an interesting observation, its meaning is not clear. It could be that proteins are on average present at many higher copies per cell than transcripts, so that variability in measuring the latter is more likely, or it could be because regulation of transcript levels is more prevalent than of protein levels, or there could be other reasons as well. In any case, this observation is not further developed in the manuscript, which reduces novelty and impact of this aspect of the study.

Minor Points

8. Are the authors sure they always have single cells in their sorted material and are not in some cases detecting doublets? How do they ensure this?

9. The titles of Supp Figures 2 and 4 are identical.

10. Regarding the RefQuant approach, there seems to be an inconsistency regarding the ratio filtering step: In the method, the authors states that "the ratios are sorted in ascending order and the first 40% of ratios are retained." Which means, to my understanding, that the *smallest* 40% of the ratios are kept, correct? Which makes sense if one assumes that higher ratios would indeed only be caused by (increasing) interference signals for other fragments. However, in the main text (p 10), it is stated that the best ratio is estimated by "taking the mean from the 40% upper quantile of ratios". Shouldn't it be the 40% *lower* quantile of ratios as stated in the method instead? Please clarify.

** As a service to authors, EMBO Press offers the possibility to directly transfer declined manuscripts to another EMBO Press title or to the open access journal Life Science Alliance launched in partnership between EMBO Press, Rockefeller University Press and Cold Spring Harbor Laboratory Press. The full manuscript and if applicable, reviewers' reports, are automatically sent to the receiving journal to allow for fast handling and a prompt decision on your manuscript. For more details of this service, and to transfer your manuscript please click on Link Not Available. **

Point-by-point response to reviewer comments for "Robust dimethyl-based multiplex-DIA doubles single-cell proteome depth via a reference channel"

We thank the reviewers for providing us with detailed and valuable feedback. We appreciate the constructive comments, which we think have helped us to significantly improve the quality of our manuscript. We are pleased that the reviewers think our work to be an 'exciting technological development' and 'technically sound and in the interesting area of methods development for single-cell proteomics' along with their appreciation of several specific points.

Reviewers 1 and 3 were quite positive overall but especially reviewer 1 had many comments and questions to our results. We believe that this is due in part to the multitude of results and different measurement scenarios, in addition to genuine issues that were unclear or underexplored in the original manuscript. Over the last months, we have performed a range of new measurements that are incorporated in the revision. Furthermore, we revised aspects of our computational analyses with the help of the reviewer's comments.

The reviewers noted that the individual pieces of our mDIA workflow were not entirely new. Specifically, our mDIA workflow has many similarities with the plexDIA workflow (Derks et al., 2022) but that paper in turn builds on many developments in (non-isobaric) MS1 level multiplexing over the years, including SILAC and super-SILAC, whereas the combination of mTRAQ with DIA was first demonstrated with the MEDUSA technology in 2014. The great advantage that DIA brings to the table is that proteome depth is not reduced by picking of redundant precursors as it is in DDA.

That said, we note that we had to develop our workflow over more than a year with an entire team to develop it to its current state, where it is now the basis for all future projects in our groups. Furthermore, as the reviewers also noted, the reference channel concept and the RefQuant algorithm are indeed novel. We also believe that the practical aspect of a robust, lossless, complete and very economical derivatization that is already fully automated is a hugely important aspect.

Regardless of this, in the revision we have added a new application area as requested by the reviewers. We build upon the recently introduced Deep Visual Proteomics (DVP) spatial proteomic technology and multiplex this for the first time. Specifically, we isolate primary cutaneous melanoma cells within different tumor microenvironments using AI and single cell type laser microdissection capture, adding a bulk reference channel. Compared to our original publication from last year, we reduced the number of single cell shapes seven-fold, doubling analysis speed, doubling number of sample and still obtaining similar

proteomics depths (together a 28-fold increase in throughput). Quantitative analysis of the epidermal and dermal melanoma cells showed clearly relevant tumor microenvironment alterations on the level of individual proteins and pathways. We believe that the combination of mDIA and spatial tumor biology will be very exciting for the oncology community (and their patients) and in precision oncology in future. This is described in the response to reviewer 2 below (page 28-31) and in the revised manuscript.

Overall, we have carefully considered each of the concerns and made changes to address them. In particular, we have performed many additional experiments and we have also applied mDIA to an entirely new application area. The most important points are:

1. Revising the text to ensure it is not misleading, overselling and in general more understandable.
2. Changing the quantification strategy by using 'Precursor.Normalised' instead of 'Precursor.Translated' in DIA-NN.
3. Detailed analysis of FDR-control and interferences in bulk and reference channel-based analysis, supporting our strategy.
4. Investigation of the reference channel concept with additional experiments.
5. Newly added experiment to demonstrate the feasibility and advantages of mDIA in a spatial, single cell type context in an oncology application (see reviewer #2, point 3, page 28-31).

In the point-by-point response below, we explain in detail the changes we have made to address each reviewer's concerns.

We used following color code:

black:	written comments by the reviewers
blue:	our explanation
orange:	modified text in the revised manuscript

Reviewer #1:

Thielert et al present an exciting technological development for multiplexed DIA, reminiscent of the recently published plexDIA workflow (Derks et al., 2022). Although mDIA is proposed to facilitate multiplexing up to 5 samples, for single-cell proteome profiling, mDIA combines two cells and a reference channel. In contrast to the plexDIA approach, the throughput is lower by 40 cells per day, but the proteome coverage appears to have increased more than two-fold.

We thank the reviewer for their positive assessment of our work and constructive and detailed comments. Using a reference channel indeed reduces throughput by 1/3rd in triple labeling and by less for higher plex labeling. As the reviewer notes, the doubling of proteome depth more than makes up for the decrease in throughput and there are many

more advantages of a reference channel as well as it represents a complete internal standard for the entire proteome just like in super-SILAC.

The authors then leverage their established mDIA method to reiterate biological findings they have made in a previous study (Brunner et al, 2022). The manuscript presents an interesting workflow that enriches the arsenal of DIA and single-cell proteomics enthusiasts alike. However, there are several important issues present in the study, rendering the current state of the manuscript not suitable for publication and requiring substantial revision of the main text and, likely, additional experiments and/or computational analyses to ensure the quality of data obtained with the established workflow. Overall, the manuscript feels a little rushed, and just to name an example, the figures should have been labeled to make it easier to refer to them correctly while reading the main text.

We have found that putting together the mDIA workflow was quite complex and over the months following first submission we have put much additional work into this technology. We hope that in the revision we could address many of the shortcomings of our initial, ‘rushed’ submission. We also apologize for not proper labeling of the figures. In the revised version, we made sure that all figures are labeled and referenced correctly.

Also, the initial literature overview of previous achievements in the field of single-cell proteomics is rather limited, leaving the less knowledgeable reader with a false impression of the true gains that were made with mDIA compared to existing capabilities and not crediting other labs active in the field sufficiently.

With the young state of the field of SCP, it is imperative that we ensure our results can withstand the greatest scrutiny, as we will otherwise never be taken seriously by the more general biologists and life science specialists. All developments are welcome, but need to undergo rigorous evaluations to ensure the quality thereof, which is something I'm not sure the authors fully succeeded with here. Whether this was due to them being under time pressure, or just overly excited to share their work with the community we don't know, but hopefully they can address the main shortcomings below more elaborately, as there is real impact expected from their work once final.

As mentioned above, plexDIA, mDIA and many of the single cell developments themselves are part of a long continuum. We do not feel that we have inadequately cited these (noting that this is not a review article). However, given that this was a main point of all the reviewers, in this revision we have made every effort to adequately cite previous work and not to claim novelty for mDIA. We also hope that mDIA and similar approaches will have ‘real impact’ in the field and this is definitely our experience in the laboratory.

Major concerns

1. The abstract appears somewhat misleading. For example, the authors write that "in single runs of mammalian cells, ..., 7,700 proteins" were quantified per channel. It should be better clarified that this does not entail single cells, and rather refer to "xx ng of complex mammalian lysate". As it stands, the sentence is not very informative. Moreover, the authors state that mDIA quantifies "close to 4,000 proteins in single cells with excellent reproducibility". However, when looking at figure 6, this is entirely not the case (!). The median PG is 2,377 proteins per cell, and it is this number that should be listed here. However, regarding this median number, I will get back to other concerns about this figure in point 10 below.

We do not see how the 7,700 proteins should refer to single cells, but since all reviewers read it this way, the fault must be ours. Following the reviewer's advice and including the new results we have adjusted the abstract accordingly (page 2 of revised manuscript).

We do agree with the reviewers that the claim of up to 4,000 proteins in single cells, while technically true, may be misleading. In any case, the record numbers of proteins identified in a single cell is a moving target of little underlying scientific merit. Therefore, we now emphasize the 'doubling of proteome depth' as in the title because it focuses more on the improvements due to the mDIA workflow and specifically the reference channel.

"Our algorithm RefQuant takes advantage of this feature and confidently ***quantifies twice as many proteins per single cell compared to our previous work (Brunner et al., PMID 35226415)****, …"*

"Single-cell proteomics aims to characterize biological function and heterogeneity at the level of proteins in an unbiased manner. It is currently limited in proteomic depth, throughput and robustness, a challenge that we address here by a streamlined multiplexed workflow using data-independent acquisition (mDIA). We demonstrate automated and complete dimethyl labeling of bulk or single-cell samples, without losing proteomic depth. The Lys-N enzyme enables five-plex quantification at MS1 and MS2 level. Because the multiplex channels are quantitatively isolated from each other, mDIA accommodates a reference channel that does not interfere with the target channels. Our algorithm RefQuant takes advantage of this and confidently quantifies twice as many proteins per single cell compared to our previous work (Brunner et al., PMID 35226415), while our workflow currently allows routine analysis of 80 single cells per day. Finally, we combined mDIA with spatial proteomics to increase the throughput of Deep Visual Proteomics 28-fold with higher proteomic depth. Applying this to primary cutaneous melanoma, we discovered proteomic signatures of cells within distinct tumor microenvironments, showcasing its potential for precision oncology."

2. Fig 2D is referenced before 2C, and upon closer inspection, 2C is not referenced at all, despite there being a substantial increase in CV on both OT and TOF with added channels - this needs to be explored in much more depth and claims substantiated! Why was 2C left out of the discussion, when it clearly demonstrates a bias in the measured ratios when extra channels are added? Is this due to false identifications and thus

quantification of the wrong signal, or are these interferences that add up to the correct signal, resulting in high variance?

Thank you for picking this up and Fig. 2C is now referenced properly.

"Quantitative reproducibility measured by coefficient of variation (CV) decreased only slightly from single to triple labeling (median 4.1% at a single channel and 7.8% at three channels) (Fig 2C)."

We were also puzzled by this unexpected result in the first submission but had reported it 'as is'. We made use of the plexDIA module in DIA-NN. Activation of this module automatically leads to the "translation" of intensity ratios between channels, resulting in "*Precursor.Translated*" values in the main output report. However, when these translated values are used for protein quantification, the CVs dramatically increase with increasing dimethyl channels. This may be due to slight retention time shifts with deuterated dimethyl labels in the medium and heavy channels, which do not occur with the Sciex mTRAQ labels that Derks et al. used.

To address this issue, we now investigated whether quantification based on "*Precursor.Normalised*" values (MS2-based quantification, also used for label-free quantification) were better suited for dimethyl-based multiplexing. Indeed, using "*Precursor.Normalised" for* quantification substantially decreased the CVs with multiplexing overall. Furthermore, the increase of CVs with additional channels was much reduced (median 4.1% at a single channel and 7.8% at three channels, compared to using "Precursor.Translated" with 4.2% at single channel and 12.6% at three channels) (**Response Figure 1**, which corresponds to **Figure 2C** of the revised manuscript).

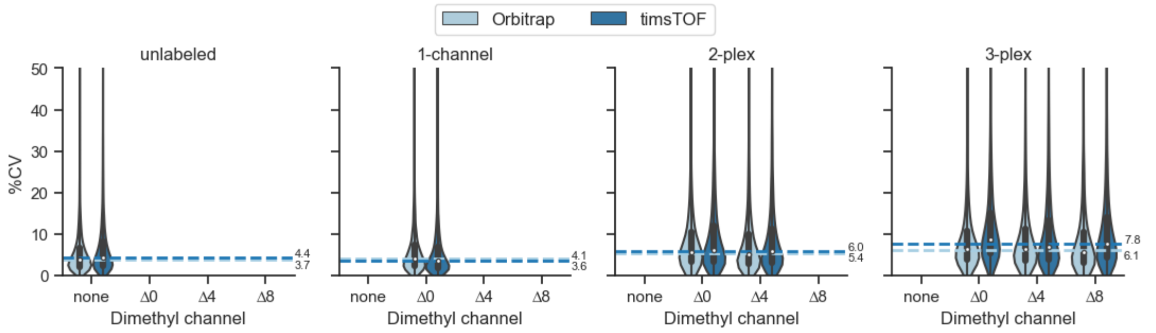

Response Figure 1, new **Figure 2C**. Coefficients of variation (CV, %) of all protein groups identified per condition for Orbitrap and timsTOF instruments. Protein group intensities were calculated using MaxLFQ-based protein quantification from "Precursor.Normalised" quantities. Median CVs are shown as dashed lines and as boxplot.

In summary of this important point, we have re-analyzed all our dataset and used "*Precursor.Normalised*" values for protein quantification, which is reflected in **Figure 2C** of the revised manuscript and the corresponding text changes in the results and methodology sections (page 30 of revised manuscript and Figure legends).

3. The text describing Fig 2E claims that the measured protein ratios largely agree, which when looking at the data, appears to be an overstatement. Measured ratios seem significantly off compared to the expected value in most cases (e.g. yeast comparison d0/d8 and e.coli comparison d4/d8), which together with 2C indicates issues with the quality of quantification. This is not the case in e.g. the plexDIA paper (Derks et al., Fig. 3). The authors should explain these phenomena. Some measurements seem to have ratio compression, while others have even higher ratios.

We agree with the reviewer and looked at this unexpected result in more depth in the revision process. Based on the systematic shift of ratios involving channel Δ0, we came to the conclusion that a small pipetting error was the issue here, prompting us to repeat the experiment. The new results demonstrate much better quantification accuracy (**Response Figure 3, new Figure 2E**). Additionally, for MS2-based quantification, we analyzed the data using both 'Precursor.Translated' and 'Precursor.Normalised' quantities in DIA-NN. As for the data presented above (**Response Figures 1 and 2**), we found that using "*Precursor.Normalised*" values increases both quantification precision and accuracy as compared to protein ratios computed by "*Precursor.Translated*" values.

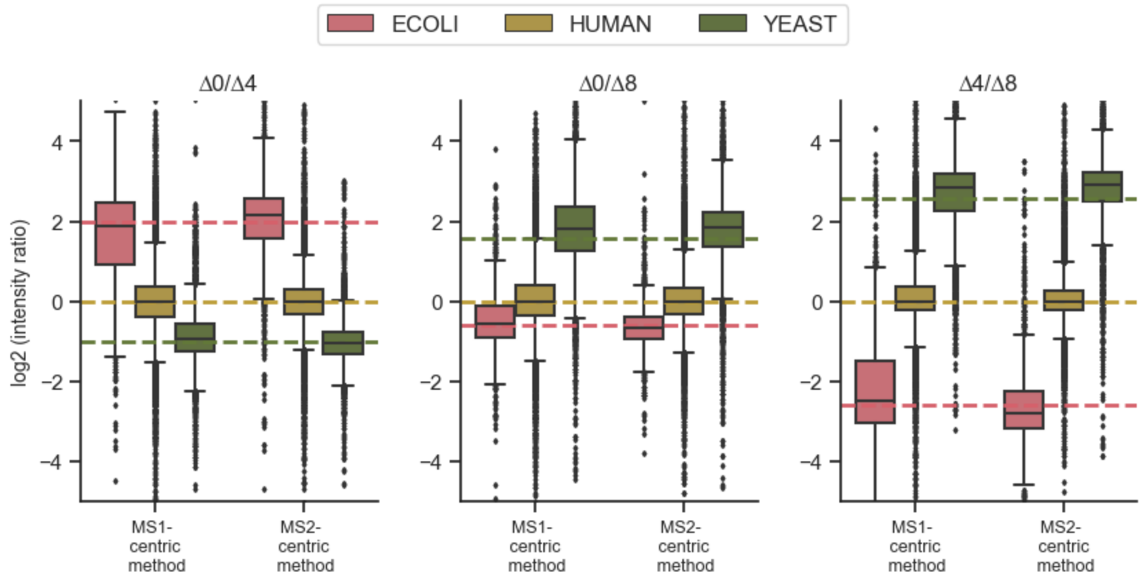

Response Figure 3, new panel Figure 2E. Side-by-side comparison of quantification accuracies between MS1-centric and MS2-centric acquisition methods in a mixed species experiment. Protein group ratios are plotted as boxplots with expected ratios as dashed lines.

4. It was nice to see that the authors employed a species mix to assess accuracy, as it is an elegant way to increase proteome complexity and even enable aspects such as FDR calculation etc. Regarding FDR, the authors missed an opportunity where e.g. one channel does not contain e.coli (e.g. d4) and where they could calculate the false transfer rate of e.coli peptides from the other channels onto this channel to assess empirical FDR. Especially in light of mDIA taking a reference channel approach (which in my opinion

should be called a booster channel), it is important to assess cross-channel ID transfer and FDR thereof.

Thank you for this suggestion. To perform the suggested analysis for the empirical FDR, we loaded 100 ng of yeast in each of the three channels and added 100 ng labeled HeLa peptides to the Δ0 channel. We then assessed the empirical FDR with either Translated.Q.Value or Channel.Q.Value <= 0.01 filters. Using Translated.Q.Value <= 0.01 was not sufficient to remove false matches of human peptides from channels Δ4 and Δ8 (as already observed for the reference channel, see Figure EV5B). In contrast, using Channel.Q.Value <= 0.01 resulted in a peptide precursor matching FDR of ~1.5% (**Response Figure 4**, new **Supplementary Figure EV3C**). As a result, we decided to stick to a Channel.Q.Value filtering of <= 0.01 for all our analyses, as before.

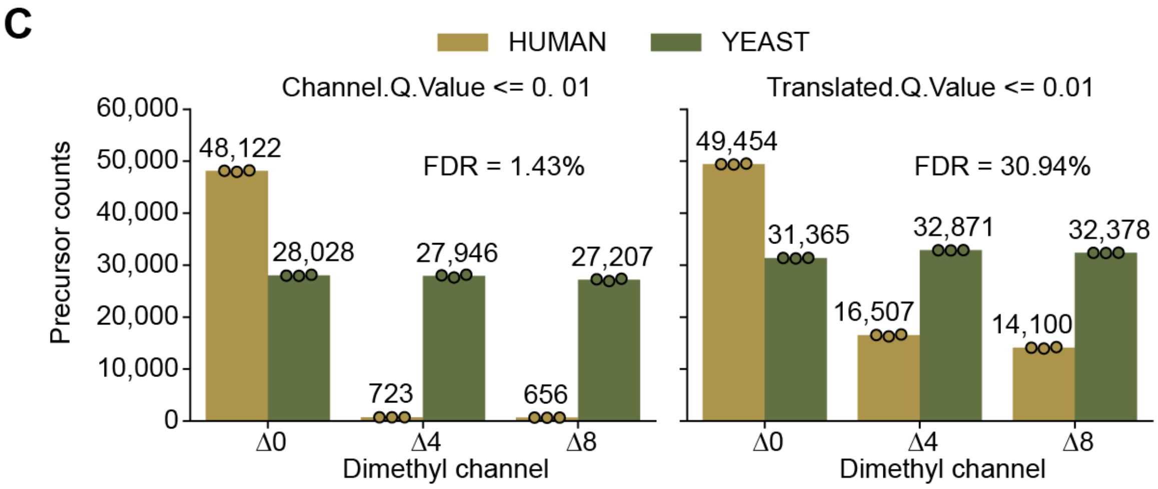

Response Figure 4, new Fig EV3C. Determination of the empirical FDR based on filter cut-offs of Channel.Q.Value <= 0.01 (left panel) and Translated.Q.Value <= 0.01 (right panel). All channels contain 100 ng of labeled tryptic yeast peptides. Additionally, channel Δ0 contains 100 ng labeled tryptic HeLa peptides (human).

"We empirically determined the false discovery rate (FDR) in a two-species experiment (Fig EV3C). For bulk and equally abundant channels, the FDR was 1.5% at a 'Channel.Q.Value' cutoff of 0.01 (see Fig EV3C for comparison of 'Channel.Q.Value' and 'Translated.Q.Value'). These results indicate absent or minimal cross-talk between the dimethyl channels."

For the reference channel approach in the case of single cells, we had already done the suggested analysis in the first submission. To summarize, we had labeled HeLa peptides with Δ0 and injected 10 ng on a timsTOF SCP (Whisper40, 31 min gradient). We then determined the peptide matching FDR empirically, by processing the raw data in DIA-NN and including the channels Δ4 and Δ8. Here, we had used four runs with empty target channels (Δ4 and Δ8, =decoy) and four runs with single cell equivalents in Δ4 and Δ8. After decoy counting at various Channel.Q.Values, we divided the maximum count of

decoys by the minimal count of targets of the four runs (most conservative way). This led to the finding that a cutoff of 15% Channel.Q.Value is below a count-based FDR of 1% (see also point 7 below for an additional experiment along these lines).

Regarding the term ‘booster channel’, we prefer ‘reference channel’ because the reference function is important in many contexts such as clinical proteomics, while it also encompasses the function of decoupling identification from quantification as well.

5. Regarding Fig 3D, a similar problem emerges as 2C, where the fact that CV values are increasing are not described nor evaluated in the text. Again, is this due to false identifications or interferences? Similarly, as the authors are keen on establishing mDIA as a 5-plex single cell proteomics workflow, they should evaluate accuracy in this labelling context too.

This is again due to the same issue as above. When using “*Precursor.Normalised*” instead of “*Precursor.Translated*” the increase in CV values is more gradual, reminiscent of the 3-plex setup (**Response Figure 5**, new **Figure 3D** of the revised manuscript).

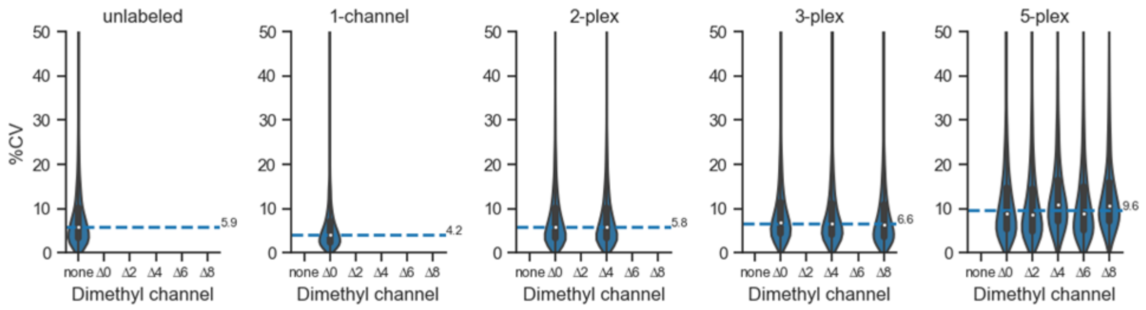

Response Figure 5, new Figure 3D. Coefficients of variation (CV, %) of all protein groups identified per condition in the dimethyl five-plex setup, using LysN as protease. Protein group intensities are calculated using MaxLFQ-based protein quantification from “*Precursor.Normalised*” quantities. Median CVs are shown as dashed lines and as boxplot.

Further, we assessed the quantitative accuracy of 5-plex in mDIA by LysN. For this, we used HeLa protein lysate digested by LysN and labeled with 5-plex dimethyl labels, followed by mixing in a ratio of 1:2:4:2:1 (Δ0, Δ2, Δ4, Δ6, Δ8). This revealed a very good quantification accuracy (**Response Figure 6**, new **Figure EV3D**).

“Similar to the three-plex mDIA result described above, CV values did not change until 3-plex, while they increased slightly in 5-plex (Fig 3D). The median fold changes of a mixing experiment largely agreed with their expected values in 5-plex (Fig EV3D).”

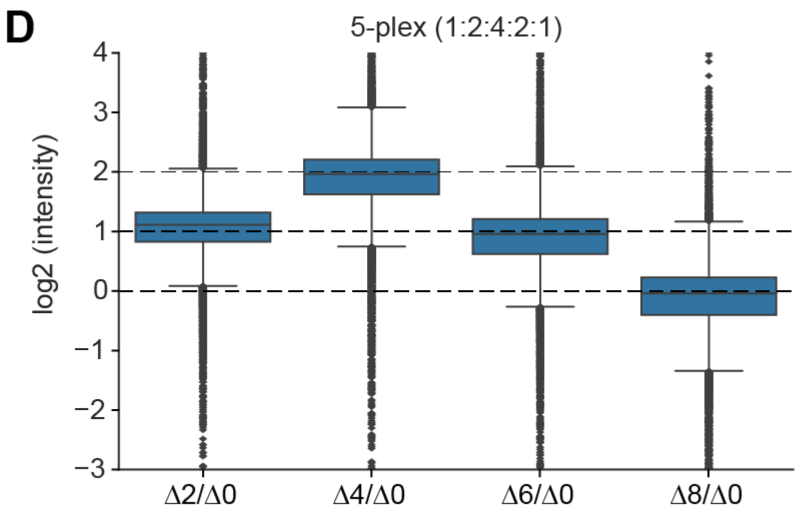

Response Figure 6, new Figure EV3D. Quantification accuracy assessment of 5-plex by LysN of labeled HeLa peptides in the respective channel (Δ0, Δ2, Δ4, Δ6, Δ8) in a ratio of 1:2:4:2:1. Protein group ratios are plotted as boxplots with expected ratios as dashed lines normalized to the Δ0 channel.

6. On pg. 9, the authors claim that "the different non-isobaric channels in the mDIA workflow are decoupled from each other in terms of quantification". Here I have to disagree, as Figs 2C and 3S clearly show that this is not true, also supported by the poor accuracy tests of Fig 2e. Similarly, on pg. 10 they "conclude that these channels are isolated from each other as expected from the mDIA concept". Again, the data presented in the manuscript does not support this claim!

Firstly, we hope that the explanation above to points 2 and 3 of this response and the additionally presented data convincingly demonstrates that the dimethyl channels are indeed decoupled from each other. The decoupling is also an important conceptual point which is true in non-isobaric labeling both at the MS1 and MS/MS level. This is not to say that quantification is perfect; there could be interferences from other channels of other proteins or ratio compression introduced by the detector of the mass spectrometer, for instance. This is clarified in the revised manuscript.

"In the bulk experiments above, we had established that the different non-isobaric channels in the mDIA workflow are decoupled from each other in terms of quantification. More importantly, different precursors that are fragmented together still do not contribute to the same 'reporter ions' as they may do in TMT labeling. Thus, the only interference in terms of quantification has to come from chemical noise of different precursors that happen to share a fragment within the MS2 resolution of the mass spectrometer."

7. The RefQuant algorithm is an interesting approach, similar to that of the carrier channel used in SCoPE-MS, and represents a major aspect of the impact of this work. The authors decouple precursor identification from quantification by using the reference

channel for FDR-controlled identification, and quantifying the signal in the remaining channels by transfer of the peak boundaries of the reference channel. The authors use the Channel.Q.Value in the single-cell channels as a filter to remove transfers to signals that are of similarly low quality as in empty channels. However, it would be important to note at what reference channel amount this parameter was determined, as Fig. EV5 B suggests a crosstalk between reference and target channels.

We thank the reviewer for appreciating the importance of the transfer of identifications in the reference channel to the target channels and investigating it in depth. We believe we have not explained this in sufficient detail and revised this part in the new manuscript.

In figure EV5B, we had shown what the results would look like if we would use a filter of 'Translated.Q.Value' <= 0.01 in DIA-NN (similar to Derks et al. in the case of mTRAQ). However, that had revealed that a Translated.Q.Value <= 0.01 was not sufficient for filtering out false matches to neighboring channels (similar to what we have observed for the bulk dataset, see **Response Figure 5** for details).

To clarify this confusing part, we now add a figure showing both the insufficient filter Translated.Q.Value <= 0.01 and the filter which we used of Channel.Q.Value <= 0.15 (**Response Figure 7**, **Fig EV5B** in the revised manuscript). The latter filtering strategy essentially removes all false identifications on the scDecoy dataset as determined by a count-based FDR (see also point 4): We performed several mDIA runs with 10ng in the reference channel, where we had left out either the target Δ4 or the target Δ8 channel or both, so that these empty channels could serve as decoys. We then calculated the ratio of counts in the decoy channel to the channel containing single cell equivalents. Defining the threshold in the most conservative way, we took only the highest scoring peptide for each protein (see **Figure EV5C**). Our results show much less than 1% count-based FDR for different experiments and batches. In the revision, this is clarified by an additional panel that directly visualizes the counts (**Response Figure 7, new Fig EV5B**).

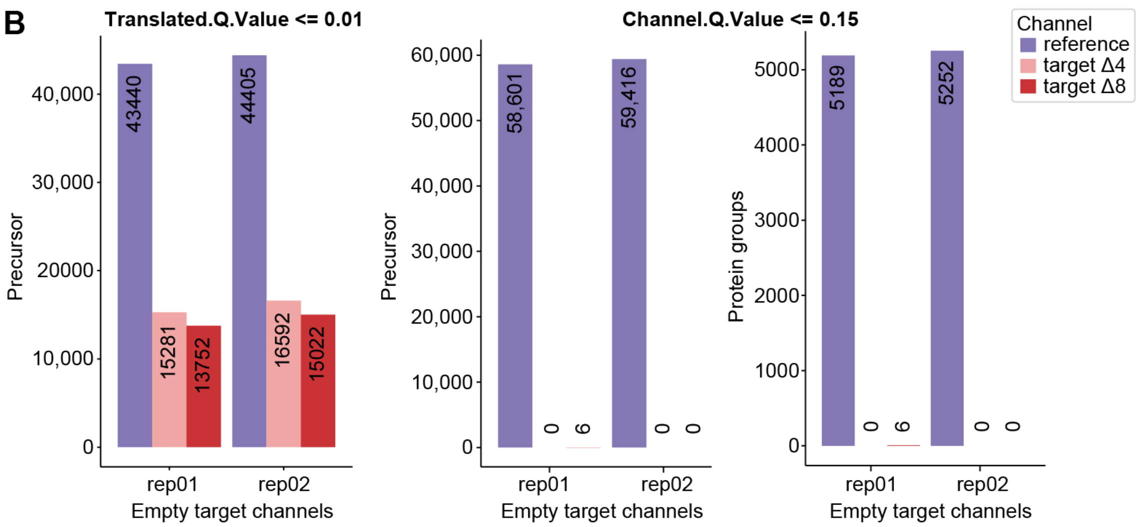

Response Figure 7, new Fig EV5B: B. Precursor identifications of empty target channels at 1% 'Translated.Q.Value' revealed more than 1% false positives (left). Precursor (middle) and protein (right) identifications of empty target channels at 15% Channel.Q.Value showed a FDR lower than 1%.

To also show that this empirical count-based FDR holds true for other samples, we repeated this experiment for mouse tissue from the liver. The reference channel contained 10 ng of bulk liver and the target channels were single shapes cut out by laser microdissection or empty. As seen before, the cutoff of 15% Channel.Q.Value again resulted in a count-based FDR of less than 1% (**Response Figure 8, new Fig EV5E**). This experiment also shows transferability of the workflow to other species and sample compositions.

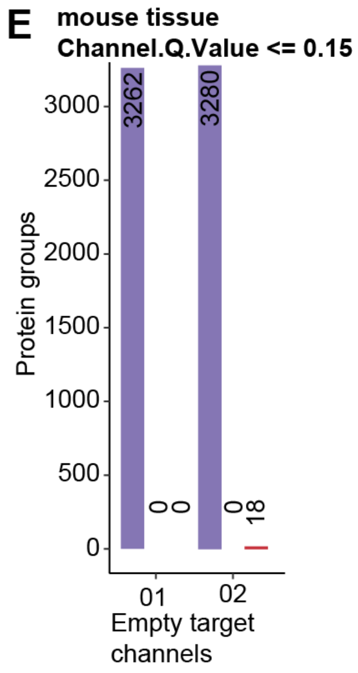

Response Figure 8, new Fig EV5E. Precursor count-based FDR derived from empty target channels with mouse liver tissue samples at 15% Channel.Q.Value is much below 1%.

"Note that this same empirically determined cutoff was also supported by experiments on mouse liver tissue (Fig EV5E)."

It should be clearly noted that their 40% quantile parameter is empirically determined and results could change with changes in both the overall LC-MS setup used, and the sample investigated. Although it is reassuring that the number of protein quantifications levels off with increasing reference channel amount, there will likely still be interferences that add up to peaks that pass the quality threshold. This could be investigated by quantifying common precursors in the single-cell channels across the different reference inputs (Fig.4C). If present, the extent of the added signal on top of the precursor signal from the reference channel would quantify the contribution of the reference channel, which likely leads to a ratio compression effect. These interferences are likely specific to the channel

used and, as it seems from Fig. EV5E, also depends on the last AA in the precursor. It would be important to investigate these interferences further and potentially propose a solution besides qualitative data filtering.

We agree and already noted that the quantile used by RefQuant was empirically derived. It is important to distinguish two different types of filtering:

1) The ID transfer filter, which defines a DIA-NN Channel.Q.Value threshold for transferring identifications from the reference channel to the target channel (experimentally determined by count-based FDR, see Fig EV5B and C and point above).

2) Beyond decoupling identification of the reference channel from that of the target channels, the RefQuant algorithm robustly deals with the inherent difficulty to determine ratios as opposed to simple quantities. This involves empirically selecting a quantile from which to take the measurement values whose median is then determined. In our experimental case, a 40% quantile worked well. While we used 40% here, we note that 100% still works, however, it introduces some ratio compression on our instrumentation. Note that the only function of the RefQuant filter is to improve quantification accuracy. It does not act as a filter for peptide identification.

To address those two filtering strategies, we had acquired different datasets. This might have been confusing and we now modified the naming of our datasets to scDecoy, scReference, scBenchmark and scQuant (Response Figure 8 and 9). We also added the information to each figure legend better describing the origin of the data used.

"To investigate the reference channel concept in mDIA, we first systematically increased its loading in the ∆0 channel (The specific datasets used for evaluation are summarized in Supplemental Table 1)."

"Supplementary Table 1: Overview of datasets used for evaluation of the reference channel

Dataset name	***Reference channel (∆0)***	***Target channels (∆4 and ∆8)***
scReference	*0.25ng to 10ng*	*Single cell equivalent*
scBenchmark	*10ng*	*Single cell equivalent*

scQuant	*10ng*	*0.0625ng to 2ng*
scDecoy	*10ng*	*Empty or<br>single cell equivalent*

Although it is reassuring that the number of protein quantifications levels off with increasing reference channel amount, there will likely still be interferences that add up to peaks that pass the quality threshold. This could be investigated by quantifying common precursors in the single-cell channels across the different reference inputs (Fig.4C). If present, the extent of the added signal on top of the precursor signal from the reference channel would quantify the contribution of the reference channel, which likely leads to a ratio compression effect. These interferences are likely specific to the channel used and, as it seems from Fig. EV5E, also depends on the last AA in the precursor. It would be important to investigate these interferences further and potentially propose a solution besides qualitative data filtering.

We have carried out the analyses proposed by the reviewer by plotting precursor quantifications of the target channels (∆4 and ∆8) depending on the amount of reference channel input using the scReference dataset (**Response Figure 9**, **Figure EV7A and B** in the revised manuscript).

"Finally, we investigated the quantitative reproducibility of single-cell equivalents in dependence on the reference channel amount (scReference). The Pearson correlations were always greater than 0.8 and did not differ between target channels ∆4 and ∆8 (Fig EV7A and B)."

The result is that quantification is largely independent of the reference channel amount, indicating low cross talk (all Person correlations > 0.8). Additionally, the channels ∆4 and ∆8 do not differ with the same reference channel amounts which suggests no channel specific interferences. Finally, there is also no major difference between lysine and arginine tryptic peptide precursors (**Response Figure 9 to 11**, corresponding to **Figure EV7A and B** of the revised manuscript).

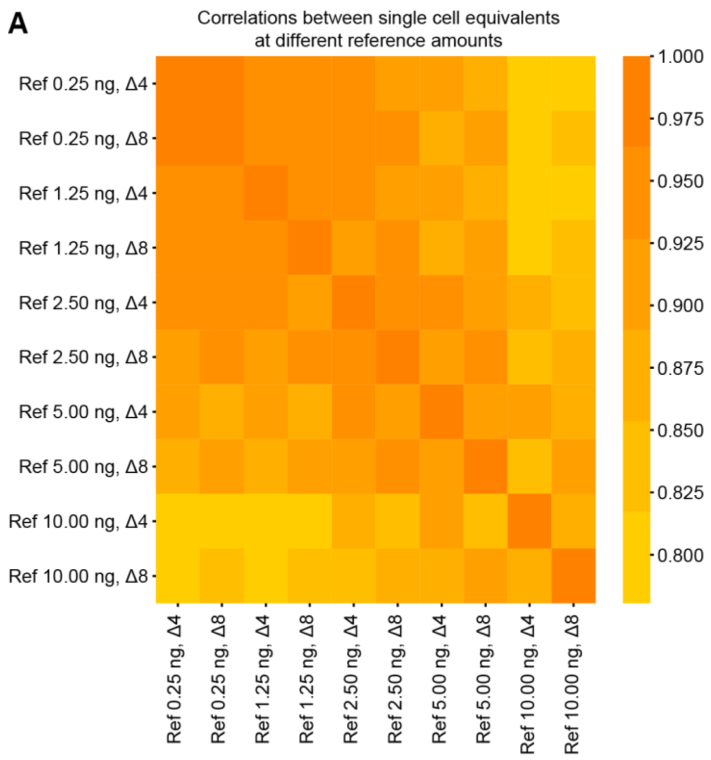

Response Figure 9, new **Fig EV7A**: Quantification accuracy in Pearson correlation coefficients between single cell equivalents in target channels (∆4 and ∆8) dependent on different reference channel amounts (0.25ng to 10ng) using the scReference dataset. The MS1 quantity is used in all cases.

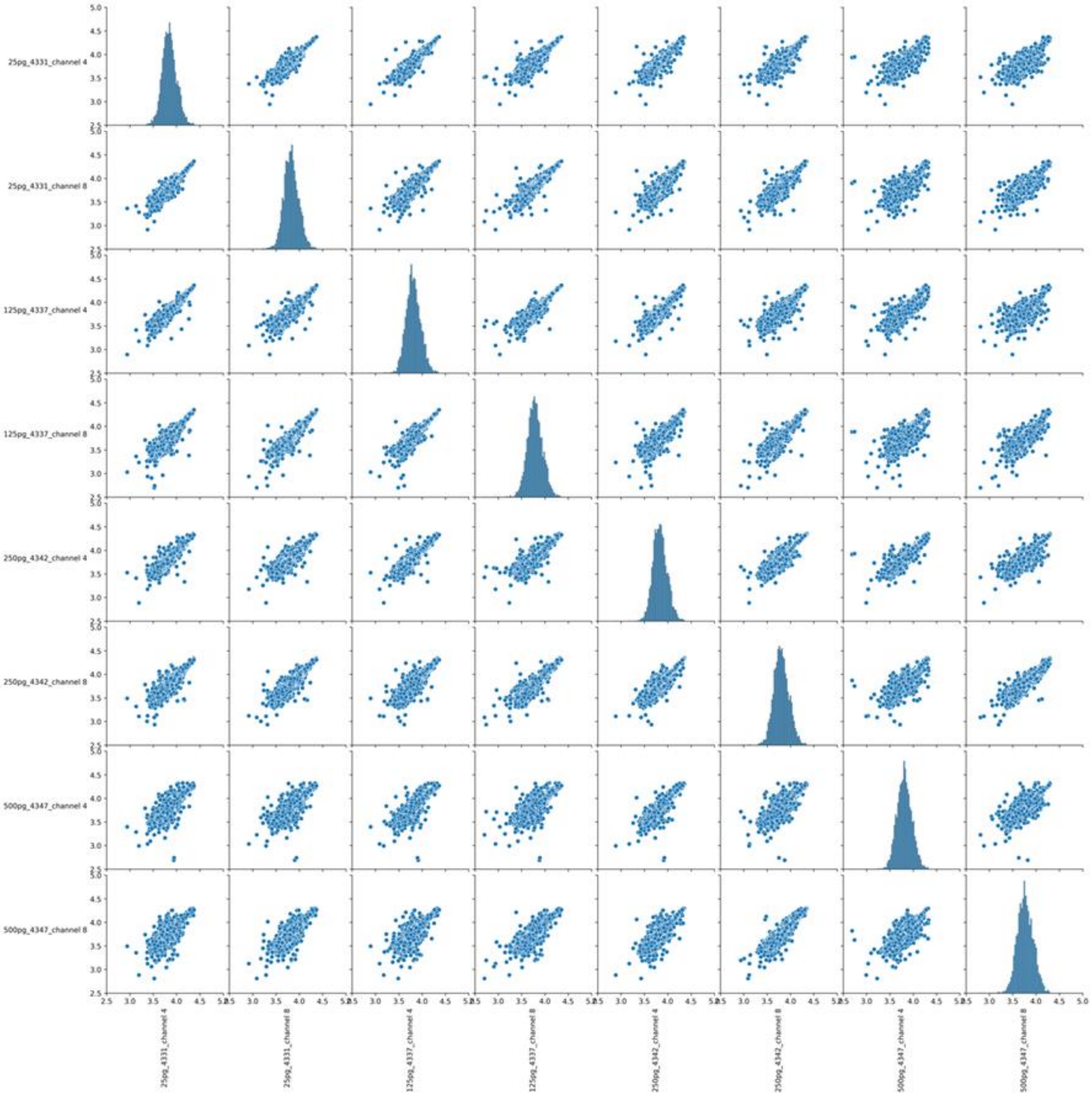

Response Figure 10, new Fig EV7B: Quantification accuracy between single cell equivalents in target channels (∆4 and ∆8) dependent on different reference channel amounts (0.25 ng to 10 ng). The spike-in amounts changed by a factor of 40, with noticeable but still mild effects on correlation between target runs (lowest correlation 0.86). The MS1 quantity is used in all cases.

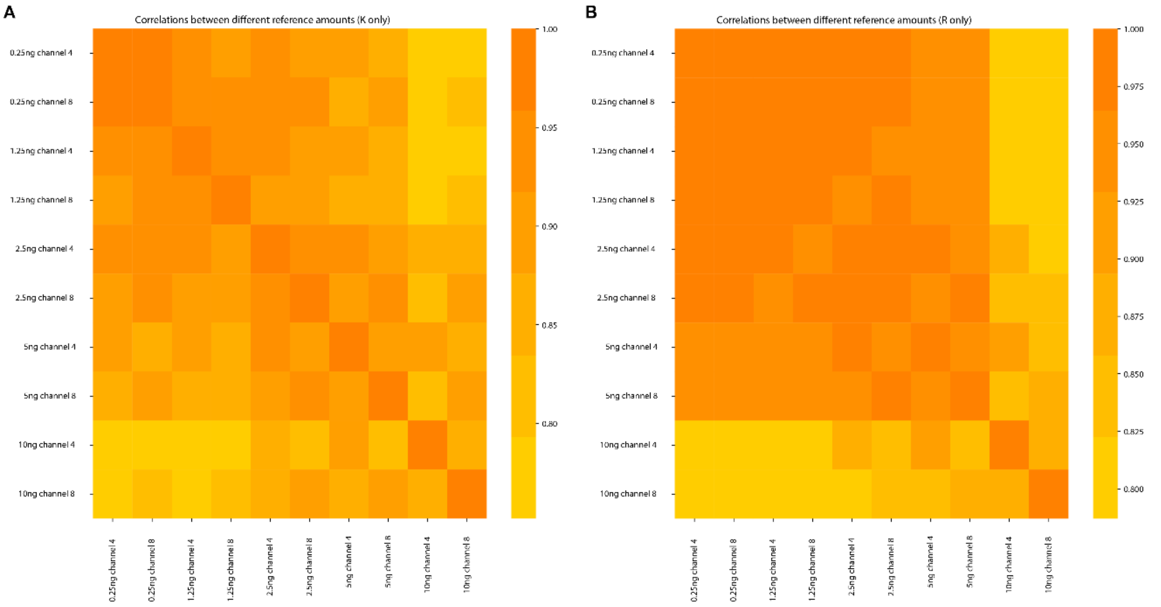

Response Figure 11: Quantification accuracy in Pearson correlation between single cell equivalents in target channels (Δ4 and Δ8) dependent on different reference channel amounts (0.25 ng to 10 ng) using the scReference dataset in dependence on the precursor of Lys (A, left) and Arg (B, right). The MS1 quantity is used in all cases.

8. Regarding Fig 5C, how did the authors come to roughly 3000 shared precursor counts at 250pg and even in 2000pg? Should the number not increase with higher loads? Moreover, why is it so low compared to nearly 3000 proteins from 250pg in Fig. 4C?

For Figure 5C, we used the scQuant dataset with 10 ng of reference channel and varying amounts in the target channel (0.0625 ng to 2 ng). We calculated ratios based on the condition of 250 pg peptide load in the target channel. This means that we divided each precursor by its median intensity at 250 pg. Therefore, any precursor that was not quantified at 250 pg of input will automatically be excluded. However, we note that the unfiltered numbers of precursors for each dataset show the increase in precursor and protein numbers with increasing amounts in the target channel (**Response Figure 12**).

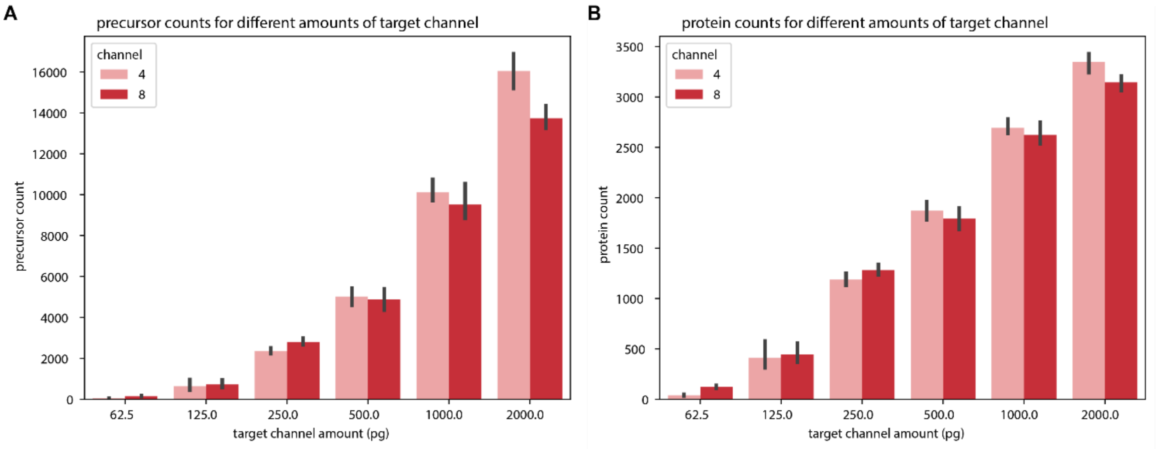

Response Figure 12: Total counts of precursors (left) and proteins (right) per amount in target channels from scQuant.

9. I am struggling a bit with their claim that it was as challenging to quantify proteins from 250pg of diluted HeLa digest as it was from single cells, as this contradicts many other observations in the field. Generally, we've seen much higher ID numbers from dilutions than from single cells. To this end, it would be imperative to know what cell sizes were used. The complete lack of FACS data in this manuscript is concerning, as it becomes difficult to connect the number of protein IDs per-cell to cell size. Also, for those few cells where the authors did ID >3,000 proteins, how can we be sure they were still single cells, and not doublets? The .fcs files from the FACS sorting procedure should allow us to resolve these concerns.

We have removed the claim from the text (see also below). Following the reviewer's request, we have uploaded all FACS data for the reader, allowing us to match single cells with their FACS signatures. The plot of forward scatter area (FSC-A) vs. forward scatter height (FSC-H) below shows no indication of doublets and no correspondence of higher protein numbers with FACS signature (**Response Figure 13, new Fig EV9A** in the revised manuscript).

"Plotting this signal against protein identifications revealed a strong dependency of input amount on proteome depth (Fig 6B), ***but is not caused by doublets (Fig EV9A)."***

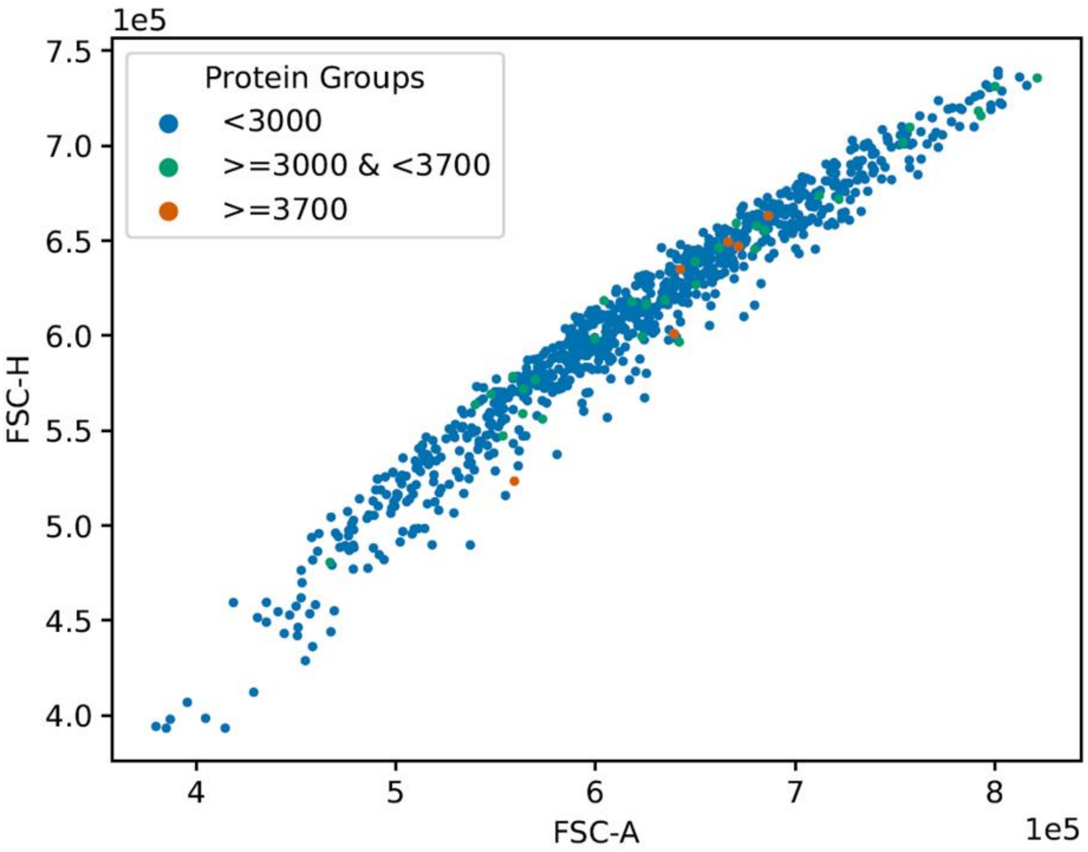

Response Figure 13, new Fig EV9A: FACS plot of single cells of forward scatter height (FSC-H) versus FSC-A (area). The data suggest absence of cell doublets and no correlation of FACS signature with protein identifications.

10. Related to point 8, only ~2,500 precursors were quantified for the 250 pg HeLa dilutions (Fig 5C). Fig 6 indicates that a similar number, but now actual protein groups, were quantified in actual single cells. This is striking as all prior research has demonstrated it to be more difficult to quantify protein groups from actual cells than dilution series (due to sample loss, imperfect digestion etc.). Can the authors explore this observation in more depth and e.g. show the difference in abundance (i.e. TIC or BPC) between the dilution samples and real single cells?

We agree with the reviewer that this is surprising. There are three possible explanations: either we have less than the desired 250 pg in our diluted down HeLa sample, we have become very efficient at avoiding any protein loss from single cells, or finally, that our HeLa cells actually contain more than 250 pg of protein.

To generate our single cell equivalents, we had used our in-house Hela digest, whose concentration we had measured by nanoDrop to be 100 ng/ul and then diluted it down to single cell equivalents. When we compared the MS1 signal of single cells to those of single cell equivalents, we found that the latter were about a factor two higher (**Response Figure 14**). This suggests that our single cell equivalents may have contained less than half of

the true single cell amount. Judging directly from the TIC and BPC is sadly not possible, since the runs include the signal from the reference channel which is higher abundant than the single cells and single cell equivalents.

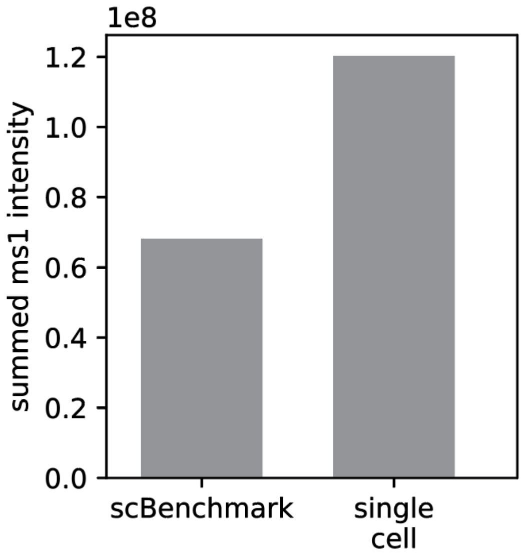

Response Figure 14: Median summed MS1 intensity of target channels (Δ4 and Δ8, single cell equivalent or single cell) in the different datasets.

11. Finally, and related to point 1, the authors provide a Source Data Table for Fig 6A which contains 893 columns, indicating that more cells were measured than noted in the main text (476 total) and filtered out before subsequent data analysis. Looking at the included table, it appears that 398 cells in the full dataset delivered below 500 protein groups, of which 372 cells produced less than 100 protein groups. If this is true, this is rather concerning, as it suggests that the experimental workflow might not be entirely robust in general, with a dropout rate of nearly 50%. If one includes these cells in the overall proteins-per-cell calculation, this would decrease the average number of quantified proteins to only ~1450 protein groups. Moreover, as this is only noticeable after MS analysis, this would also lower the throughput of mDIA SCP to only 40, rather than the claimed 80 cells per day. Perhaps the FACS data can provide more insights into this, and this aspect needs to be evaluated in much more detail.

We are aware of this dropout rate of our single cells. This is partly an issue with FACS as we visually confirmed by placement of the droplet in the 384 well. In subsequent work on single shapes using the Deep Visual Proteomics technology (Rosenberger et al., bioRxiv, 2022), the dropout rate was lower (only around 9%), showing that this is not an inherent problem of the mDIA technology applied to single cells. Furthermore, ongoing work with our own software (AlphaDIA) on the same data also produces much lower dropout rates indicating that our conservative FDR calculation to limit the transfer of IDs in DIA-NN could still be improved, which would lead to lower apparent dropouts. In any case, the dropout rate is not related to our mDIA concept.

In the future, we aim to improve our workflow with different sample preparation methods including using instruments like the CellenOne (ScienIon) in which the cell is more clearly visualized and can be examined more directly than in FACS data.

12. A PCA or UMAP plot of the single cell results would be nice to see, to determine if the biological variation due to the cell cycle is captured and no channel (d4/d8) biases are present.

Thank you for pointing this out. We now included a PCA plot showing that there are no biases by the channel. However, since we do not have any prior assigned classes and small changes given our unsynchronized sorting, there are no clear classes of cell cycle states (**Response Figure 15**).

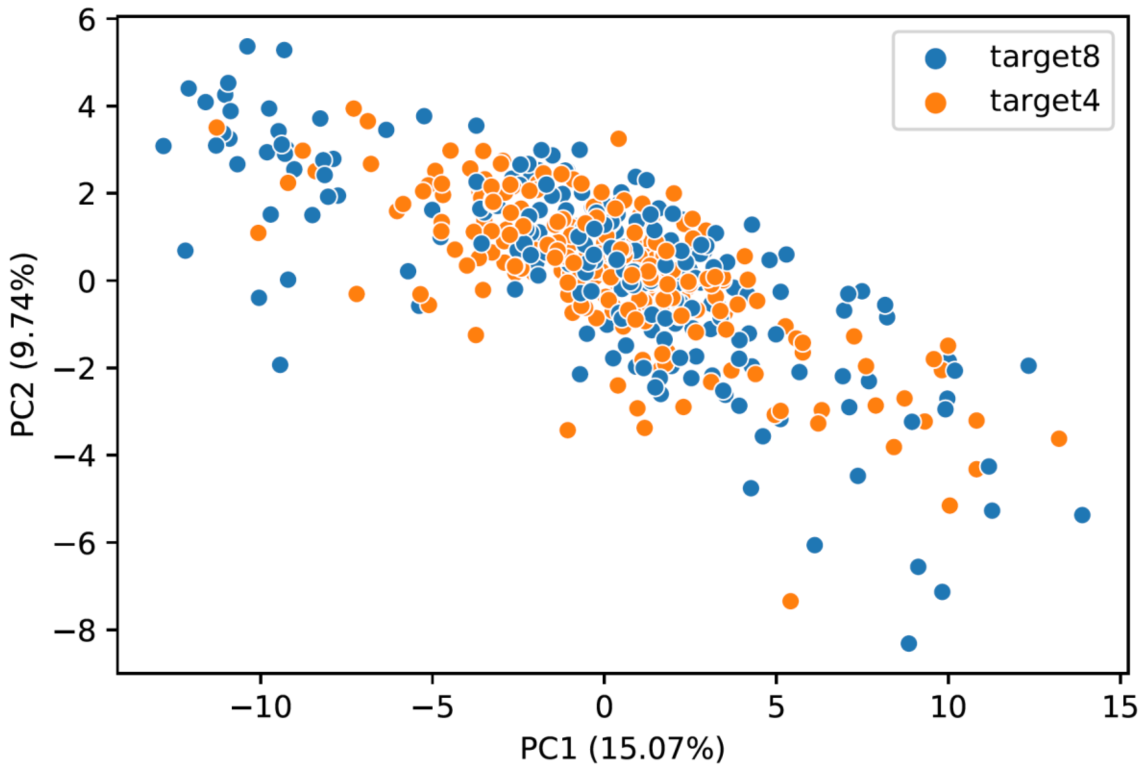

Response Figure 15: Principal component analysis (PCA) of unsynchronized single- sorted HeLa cells. The PCA does not show a bias for the two different target channels Δ4 and Δ8.

Minor concerns

13. The first half of the introduction covers the field of single-cell proteomics (SCP) in a very shallow manner. The breakthrough studies are barely referenced or wrong references are used (e.g nanoPOTS [Kelly, 2020], where the actual study that established the method is Zhu et al, 2018). The authors should ensure to appropriately credit previous research that propelled the field forward. For example, other studies that have also used

384w plate format with FACS were Liang et al 2021, Specht et al 2021, and Schoof et al, 2021, and deserve mentioning here.

We have added more references in the introduction to give credit to breakthrough studies in the field of single cell proteomics. We hope that the introduction does now represent the single-cell proteomics field better (page 3 and 4 of revised manuscript).

14. Regarding Supplemental Text EV7, I can follow the authors' argumentation until the point where they claim that higher abundances of the reference channel will result in lower variation values. Certainly, the coefficient of variation (CV) will decrease, however, the absolute variation might still increase. The authors should use the data they have to test the consideration made in EV7.

We are not entirely sure what the reviewer means by CVs in target channels decreasing as a result of increased amount in the reference channel, but absolute variation increasing. However, we have performed the analysis suggested, namely to plot the CVs in the Δ4 and Δ8 target channels containing single cell equivalents as a function of the amount in the reference channel. From quintuplicate measurements we clearly see that the CVs decrease until the maximum value measured, which was 10 ng (**Response Figure 16**, new **Figure EV7C**). Perhaps the reviewer means that the absolute variation between target channel and single channels would increase. This is obviously true but already discussed above in relation to RefQuant. In the revision, we have tried to make our statement clearer.

“As the higher abundances of the reference channel stabilizes its signal, we conclude that the overall variation of the ratios will decrease with higher reference proteome amounts. We also demonstrated our assumption in the target channel by using different amounts in the reference channel comparing the CVs of the single-cell equivalents in the target channel (scReference dataset), as the CVs in the target channel decrease with higher reference channel amounts (Fig EV7C).”

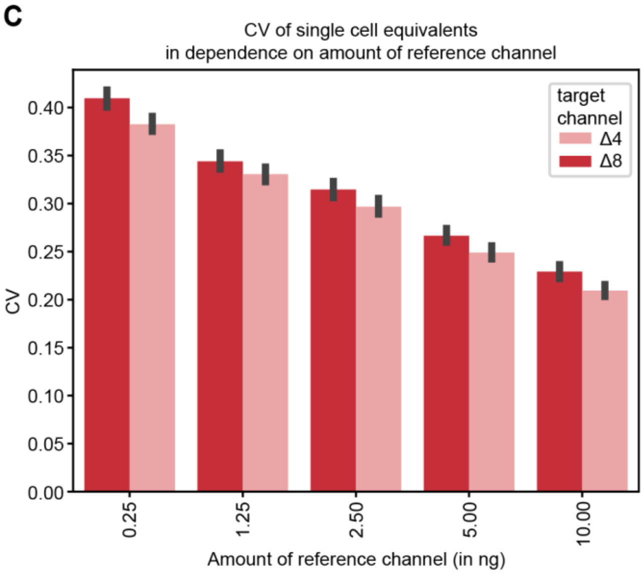

Response Figure 16, new **Figure EV7C**: Decreasing CV values within the data of single cell equivalents as the amount in the reference channel is increased from 250 pg to 10 ng (by using the scReference dataset).

15. I am lacking a bit more detail on the spectral libraries that were used. Were the same libraries of AlphaPeptDeep used for both TOF and OT data? Would it be expected that mass analyser-specific spectral libraries should be used for best performance? Also, I couldn't find the actual libraries in the data submission, something that would be imperative to evaluate their performance independently.

We agree that this is an interesting point. Yes, indeed, we utilized the same spectral libraries for both timsTOF and Orbitrap raw data processing in DIA-NN. These libraries were generated using dimethyl-labeled DDA datasets acquired on an Orbitrap platform. We observed that employing an Orbitrap-trained library led to higher identification rates for timsTOF data as compared to a timsTOF-trained library, without any significant differences in CV values (**Response Figure 16A**).

This somewhat unexpected result is due to the fact that AlphaPeptDeep relies on de-noised and centroided peaks of fragments for MS2 training. This is particularly advantageous for Orbitrap data because Thermo has a unified peak picking (de-noising and centroiding) algorithm that is used by most of the software tools, such as MaxQuant, MSFragger, DIA-NN, Spectronaut, etc. As a result, the fragment intensity values are almost the same among different software. However, Bruker does not provide such a unified peak picking algorithm for developers, so each software tool does peak picking on its own to extract fragment intensities, making the intensity distributions quite different for different software. As an example, we show that fragment intensities of only 63% precursors are very reproducible (>0.9 correlations) between MaxQuant and MSFragger results (the percentage is >90% for the Orbitrap cases) (**Response Figure 16B**). Although we cannot access DIA-NN's peak-picking intensities, we guess this also applies to DIA-NN and this may explain why the identification numbers decrease with timsTOF-trained libraries.

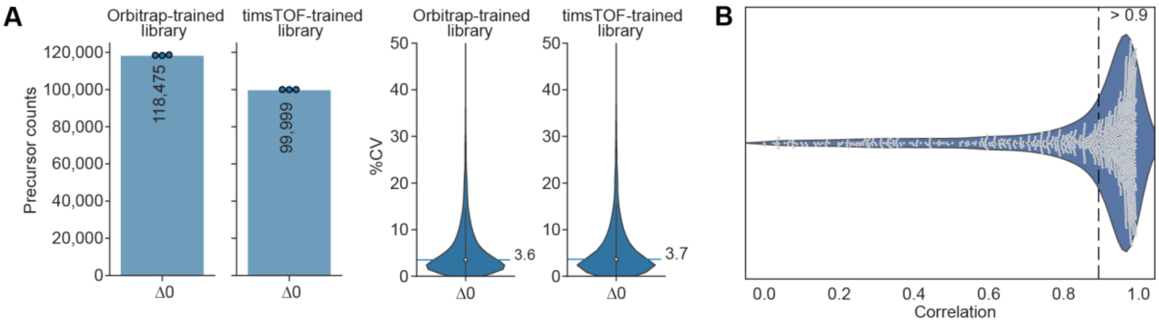

Response Figure 16. Comparison of Orbitrap-trained and timsTOF-trained libraries. A. Number of precursor identifications (left panel) and %CVs (right panel) of the 1-channel timsTOF data (data from Figure 2 of the revised manuscript) when searched against the two libraries. B. Correlation of fragment ion intensities of 1-channel dda-PASEF data processed with MaxQuant and MSFragger.

The .speclib libraries had been added in the folder 'Libraries.zip'. In the revision, we now also include the .tsv files of each library.

16. I really liked the author's MS1 vs MS2 quant comparison, this was a very nice feature to add and seems relevant for single-cell DIA which is as of yet, still relatively unexplored. However, if the authors are keen on including this in the manuscript, I would like to see more exploration on aspects of e.g. how comparable the OT methods are vs TOF? Was it a fair comparison based on window size/resolution & cycle time? There was not a lot of detail provided for the data acquisition schemes in the methods, which should be improved (Supp. Tables were not much help here either), and could the authors explain how they optimised MS1 vs MS2 methods for OT? On timsTOF, there were 2 clear methods used, but on OT it appears the same method was used for both MS1 and MS2. This is very likely sub-optimal, and possibly explains the observed performance differences between OT and TOF.

Apologies if the data presented was confusing and if the provided details on the MS methods were not sufficient. We have improved this in the revised manuscript. However, we believe it is beyond the scope of this paper to undertake a detailed investigation and comparison of two manufacturer's instruments and their many different parameters and system components. Additionally, the requested comparison of MS1 and MS2-based methods on the Orbitrap has been performed in the plexDIA paper (Derks et al., 2022) and this is now noted.

"In addition to our initially designed dia-PASEF method, we generated an alternative method, consisting of multiple MS1 scans in between dia-PASEF scans of each duty cycle (Fig EV4B), ***similar to what was performed previously on Orbitrap instruments (Derks et al, 2022).****"*

In brief, in our original manuscript, we first focused on the Exploris instrument for the bulk measurements alongside the timsTOF instrument. This is because the Orbitrap has a large user base and we believe our mDIA results will be of interest to that community. This was done on both instruments by MS2-based methods. We added details of the methods below (**Response Table 2**, new **Supplemental Table 6**).

We then switched to only the more sensitive timsTOF for the reference channel concept and the single cell (and now DVP) measurements as well as for the detailed assessment of quantitative accuracy achievable (MS1 vs. MS2 methods) on bulk and with the reference channel. Our manuscript is already very long and an all against all matrix would make it even longer.

Method explanation:
We started off with MS2-optimized methods for both platforms. For the Orbitrap instrument, we used a 44 m/z windows acquisition scheme, with a cycle time of approximately 3.2 seconds and an average of 4 data points per peak (previously published method, Steger et al, Nature Communications 2021; https://doi.org/10.1038/s41467-021-

25454-1). For the timsTOF instrument, we instead used 20 diaPASEF scans, as an optimal compromise between acquisition speed and quantification precision such that we obtain 5 data points per peak at a retention length of about 11.2 seconds, corresponding to a cycle time of 2.23 seconds. We then generated a MS1 centric methods for the timsTOF, with comparable cycle times to the MS2-centric methods. We reduced the number of diaPASEF scans to 16 and increase the number or MS1 scans to 4.

Response Table 2, new Supplementary Table 6. Important parameters used in the MS2-optimized acquisition schemes for both Orbitrap and timsTOF and MS1-optimized for timsTOF.

	Orbitrap	*timsTOF*	
Acquisition scheme	*MS2-optimized*	*MS1-optimized*	*MS2-optimized*
Number of MS1 scans	*1*	*4*	*1*
Number of MS2 scans	*44*	*16*	*20*
MS1 Resolution	*120,000*	*40,000*	*40,000*
MS2 Resolution	*30,000*	*40,000*	*40,000*
Cycle Time (s)	*~3.2*	*~2.12*	*~2.23*
Data points per peak	*4*	*5*	*5*

17. The idea of a reference/carrier channel in plexDIA has been laid out multiple times already which should be attributed in the manuscript. https://www.nature.com/articles/s41587-022-01389-w, https://www.biorxiv.org/content/10.1101/2022.11.05.515287v1

We are a bit confused here as the reviewer previously commends us for the novelty of the reference channel, as does reviewer 3. In fact, the Derks et al. paper does not explore the concept of a reference channel, which in any case arguably goes back to the super-SILAC concept (although not in the DIA context). One challenge in our project was that DIA-NN, while incredibly useful, explicitly does *not* support the concept of a reference channel.

18. Budnik et al, 2018 introduced the term "carrier channel" and not "booster channel". This terminology should be used correctly when directly citing the original paper.

Thank you for pointing this out. We have fixed it.

19. On pg. 8, the authors claim that the derivatization reaction does not complicate the workflow, but if on-tip labelling is used for single cells, then how are labeled peptides pooled onto their final EvoTip? I would expect this adds an additional elution step?

Thanks for mentioning this. The labeling is in fact a great strength of our workflow especially in connection with the Bravo robot and the Evosep tips. The single cells are labeled in solution in their respective wells of the 384-well plate. The reference channel (which was processed separately) is loaded first on the Evotip, followed by direct depositing of the single cell proteomes (Δ4 and Δ8) onto the same Evotip. Therefore there is no additional step that could complicate the workflow. This is now pointed out more strongly in the revised manuscript.

"Each of the three in-solution labeled digests in a 384-well plate are sequentially deposited onto the same Evotip, thus, the derivatization reaction does not complicate the workflow and is completely transparent to the user. The entire sample preparation, including the combination of the labeled channel while loading of the Evotips,"

20. It would be nice to see some data on the claim of a 2s FWHM peak width.

This is directly from the FWHM in the output of DIA-NN; mean of 0.045 min in our scBenchamark dataset, which equals 2.7s for our scBenchmark dataset, as the reader and reviewer can check for themselves.

21. In the main text the authors state that the upper 40% quantile is used, but in the method section "The ratios are sorted in ascending order and the first 40% of ratios are retained." This would suggest the lower 40% quantile. We assume that the latter one is the correct one.

Sorry for the confusion, indeed the latter one is correct. We corrected this in the revised text.

Reviewer #2:

This paper by Thielert et al. presents a multiplexed workflow using data-independent acquisition (mDIA) using dimethyl labeling of bulk or single-cell samples in an automated way using a liquid handling system. Bulk analyses of mammalian cells in three-plex of tryptic peptides quantified as many as 7,700 proteins per channel. Use of Lys-N for digestion extension of non-isobaric labeling to five-plex quantification. The use and potential added value of a reference channel in mDIA is demonstrated as well as sample

analysis throughput of as high as 80 single cells per day. The biological study described extends the previously reported concept of a stable proteome.

We thank the reviewer for the accurate summary of our paper.

Major Issues:

1. This report by Thielert and the Mann group combines several previously reported methods, some well described elsewhere by this group i.e., Brunner et al. processing and analysis methods for single cell analysis in DIA mode, Slavov lab for non-isobaric labeling of samples for multiplexed DIA of bulk and single cell analyses (Derks et al. NatBiotech 2022) and Wu et al. (Chem Commun 2014) for development and use of a 5-plex version of the dimethyl reagents developed by the Heck lab. The present paper combines all of the above in DIA mode which, while interesting, is not particularly novel.

We acknowledge that many of the single components of the mDIA workflow have been described in different or similar contexts before. We have done our best to cite each and every one of those previous developments. While a paper much certainly has novelty, we believe there is nothing wrong with building on previous efforts, a core tenant of the scientific method. We believe that the aim of our workflow as of previous multiplexing efforts is very important and requires the contribution of many in the community.

We believe that the novelty in this manuscript comes from the combination and analytical characterization of all of the aspects that we have brought together. Additionally, the 5-plex in mDIA is novel and not irrelevant as it doubles throughput when also using a reference channel (The above cited Wu et al, uses 5-plex labeling at the MS1 level only, the fragments are superimposed on each other because they use LysC instead of LysN. Dimethyl labeling for proteins is usually attributed to Hsu, 2003, whereas the Heck lab greatly expanded the use of this technology). The novelty of 5-plex at MS1 and MS2 level by LysN is also credited by reviewer #3.

2. The one aspect of the paper that is potentially novel and of interest is the impact of the use of a reference channel on depth of coverage, reproducibility, quantitative accuracy and ability to link data obtained across large numbers of multiplexed DIA analyses. While the reference channel concept is well known and commonly used in multiplexed DDA, and its potential value clearly described for DIA in the Discussion portion of the Derks et al. paper, the present report goes beyond the Derks et al. report in using a reference channel and showing its value in quality controlling target-channel quantitation. A paper focused on this interesting aspect could be valuable especially in the context of single cells and large sample numbers. However, the demonstration presented is rather simplistic (HeLa cells using a HeLa common reference channel at higher load levels) and does not provide experimental results that explain why the reference channel appears to work to boost numbers and reduce the spectral noise. Could the increase in identification simply be an effect of adsorptive losses during sample preparation/acquisition, and the lack of further increase above 5 ng load for reference

channel be due to saturation? The impact of the composition of the reference and the absence of carrier peptides in the presence of target peptides was not evaluated. What if it differed from that of the analyte samples tested? At the presumed heterogenous single cell level, how would this be dealt with?

We indeed believe the reference channel concept is of great value, which has further been born out in our experience since first submission. We also agree with the reviewer that the reference channel would also naturally have some of the advantages of a carrier analyte. As suggested by the reviewer, we now directly determined the impact the reference channel on preventing absorptive losses or otherwise 'helping' the single cell target channels.
For testing this, we measured single cell equivalents in the target channels (Δ4 and Δ8) and added 10 ng of either unlabeled HeLa (minus reference channel) or Δ0-labled HeLa (plus reference channel). With this experiment, the saturation and losses upon adsorption should be the same and should therefore result in similar proteomic depth if adsorptive losses are the reason for the increase in identification. However, we show that the identifications using a reference channel is higher in the target channels then when just using the same amount of spiked-in unlabeled HeLa sample (no reference channel) (**Response Figure 17, new Figure EV5F**).

With this, we think that we can show that the reference channel is the important factor for improvement and not a simple sample acquisition effect. This data also shows the same increase in protein identifications similar as shown in Figure 4C, indicating that the effect is not due to adsorptive losses during acquisition.

Additionally, we note that the sample preparation of the single cells is performed individually, meaning that the reference channel has no impact on the sample preparation of lysis, digestion and sample losses of the individual single cell.

"Additionally, to show that this identification increase is due to the reference channel and not simply because of adsorptive losses during sample analysis, we compared the identifications in the target channels with reference channel and without, while spiking 10 ng of unlabeled HeLa instead. Identifications from single cell equivalents adding an unrelated 10 ng proteome were 1247 proteins, while using a 10 ng reference channel were 2018 protein groups (mean of five replicates in each target channel). Thus, the increase in identifications is overwhelmingly due to the reference channel, rather than simply an effect of adsorptive losses (Fig EV5F)."

F

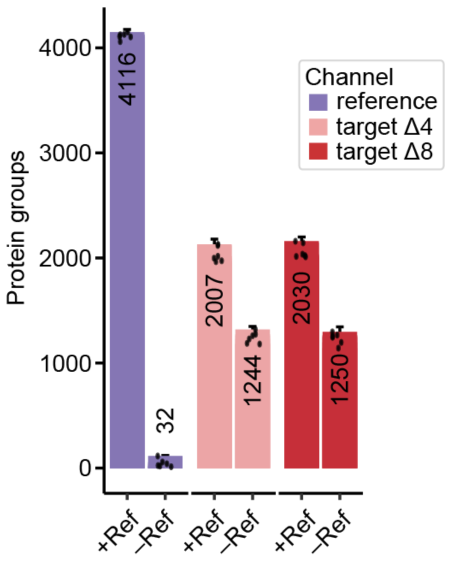

Response Figure 17, new Figure EV5F: Impact of the reference channel on identification in the target channels of single cell equivalents. We compare single cell equivalent in the target channels with (10 ng Δ0-labeled HeLa) and without reference channel (10 ng unlabeled HeLa).

3. Importantly, there is also no new application of the methods that sheds light on some interesting biology. I view this as essential for a paper in MSB. Instead, the authors extend an already published observation that there is a stable core proteome (at least in HeLa cells). Without a relevant new application (such as that cited in pre publication form from these same authors by XXXXX et al.) of the reference method the paper is more suited to a specialized proteomics journal.

Further, the introduction of the reference channel to non-isobaric labeling is another added combination, which has not been shown before. Additionally, we now added an additional experiment to show biology with our technology. Here, we used our Deep Visual Proteomics (DVP) technique and compared primary cutaneous melanoma in different tumor environments by cutting only 100 shapes (corresponding to 20 cell equivalents). We think that this experiment is a good use-case to show its feasibility.

"mDIA advances single cell type resolved spatial proteomics in oncology
We reasoned that the multiplexing and quantitative attributes of mDIA should be of great advantage in tissue proteomics, especially in the context of diseases. In particular, we wanted to integrate it with our recent technology termed Deep Visual Proteomics (Mund et al, 2022). DVP combines artificial intelligence-driven image analysis of cellular phenotypes with automated single-cell laser microdissection and ultra-high-sensitivity mass spectrometry, effectively linking protein abundance to complex cellular phenotypes while preserving the spatial context. To explore the advantages of mDIA in this context,

we profiled cancer cells in-situ in primary cutaneous melanoma. Using routine histopathological markers, we segmented single melanoma cells and further stratified them according to their spatial location to the epidermal or dermal compartment, thereby taking the tumor microenvironment into account (Fig. 7A). In recent work on superficial spreading melanoma, we had cut 700 shapes from 2.5 µm-thin formalin-fixed paraffin-embedded (FFPE) tissue sections (Mund et al, 2022). We found that the mDIA workflow seamlessly integrated into the DVP pipeline. For the design of the reference channel, we used bulk digest from a consecutive tissue slide, containing mainly but not exclusively tumor material. We expected mDIA to be much more sensitive in this context and therefore only excised 100 single cell shapes (corresponding to 20 cell equivalents at 2.5 µm thickness). We added the reference channel to the epidermal and dermal target channels in a roughly estimated ten-fold excess. With a Whisper20 SPD gradient (58 minutes active gradient), we quantified 4,000 protein groups in melanoma cells within the epidermis and 2,740 in the dermis, similar to our previous report on the same cell type. However, our mDIA-DVP pipeline used seven times less input amount, a shorter gradient (1 hour vs. 2 hours) and measured two samples per run. This represents an overall 28-fold increase in throughput without losing proteomic depth. Importantly, cutting time on the laser microdissection instrument is also decreased seven-fold, a large gain for the overall DVP pipeline.

To test the cell-type specific and biological validity, we looked for typical melanocytic markers (SOX10, MITF, DCT, MLANA, PMEL, TYR, TYRP1) (Belote et al, 2021) in our data. We detected all but TYRP1, including SOX10, the most important (transcription) factor of melanocytic lineage used in routine clinical diagnostics (Fig. 7B). Interestingly, expression of these identity markers was lower in dermal melanoma cells, highlighting the role of the tumor microenvironment in cancer-cell identity and dedifferentiation (Fig. 7C). Next, we sought to assess proteomic differences between epidermal and dermal melanoma cells. In principal component analysis (PCA), component 1 clearly separated dermal from epidermal melanoma cells (Fig. 7D). Importantly, this was irrespective of the target channel used for labeling individual replicates, which we ascertained by label swapping. Compared to melanoma cells in the epidermis, melanoma cells located in the dermal compartment had significantly higher expression of proteins involved in remodeling of the extracellular matrix (e.g., DPT, COL1A2, COL1A1, COL3A1, COL6A1, DCN, TGFBI, PRELP, DCN, FGA, FN1, and LUM) (Fig. 7E). In contrast, melanoma cells of the dermal compartment had significantly lower expression of TACSTD2 and LGALS7, amongst others (Fig. 7E). While downregulation of TACSTD2 is part of a gene expression profile signature that predicts increased risk of cancer progression, LGALS7 is involved in promoting cellular apoptosis and could therefore lead to increased tumor cell survival (Gerami et al, 2015; Biron-Pain et al, 2013). The protein Keratin 15 (KRT15), which is normally expressed in basal keratinocytes of the epidermis, has also been found to be associated with tumor stage and prognosis in metastatic melanoma patients, with higher expression in primary tumors and loss in metastases (Han et al, 2021). We also observed a reduced expression of KRT15 in cells located in the dermal compared to the epidermal melanoma cells according to the depth of invasion (Fig 7D). Pathway enrichment revealed multiple and highly relevant signaling cascades enriched in dermal melanoma cells, such

as senescence, PI3K-AKT signaling, and IL-18 signaling. Taken together, the combination of mDIA and DVP enables high-throughput, in-depth and biologically relevant insights of cancer cells from single patients, including the spatial component.

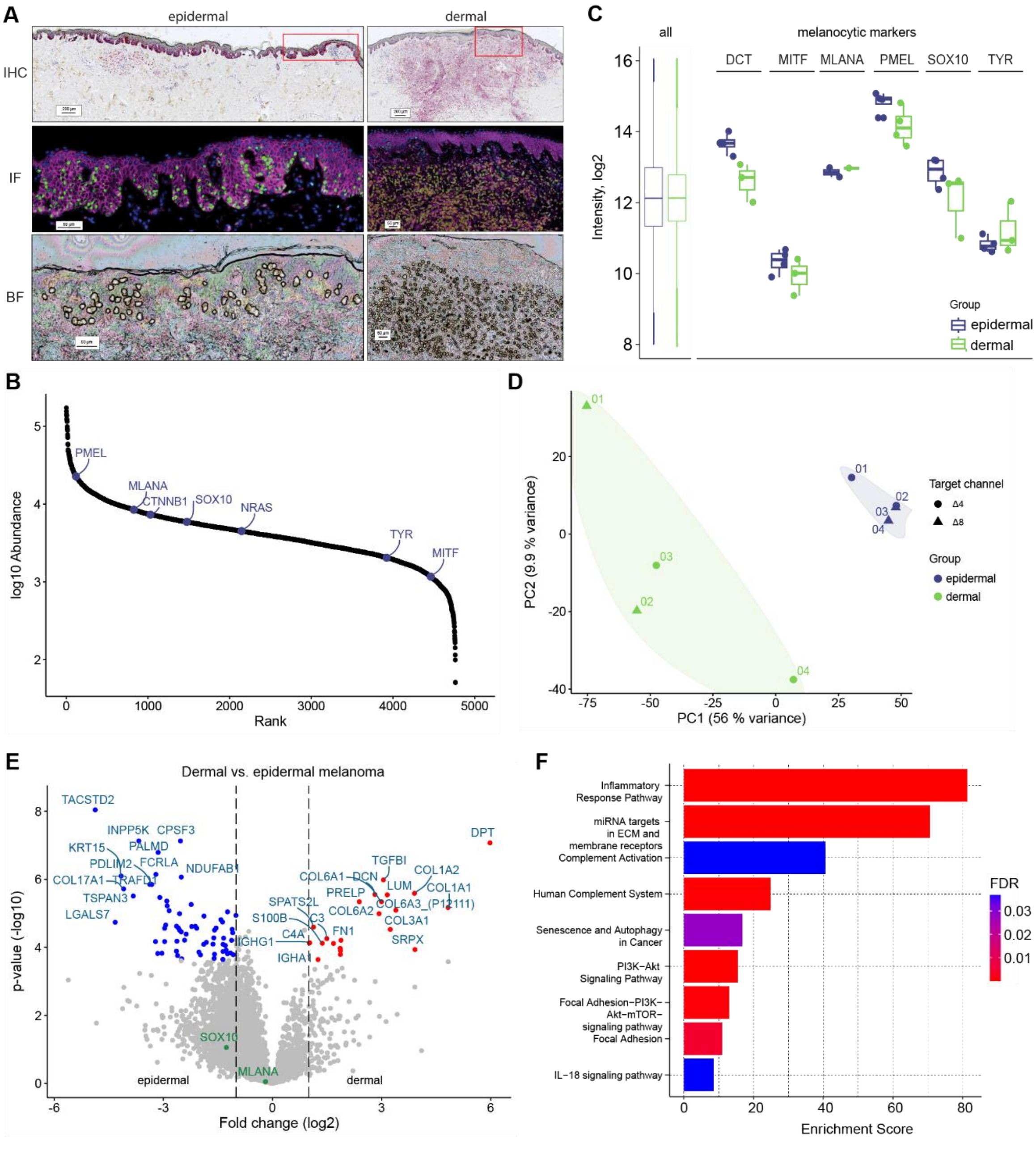

Response Figure 16, new **Fig 7**: A. Macroscopic overview of primary cutaneous melanoma (type SSM, Breslow 3.5mm) including Melan-A immunohistochemistry (IHC, pink) and CD44/Sox10 immunofluorescence (IF, pink/green) before, as well as a brightfield (BF) image after laser microdissection. Segmented melanoma cells are highlighted (yellow outlines). Quadruplicates of 100 shapes (20 cell equivalents) were laser-microdissected from the epidermal (left) and dermal (right) compartment using Deep Visual Proteomics (DVP). B. Rank plot of all proteins identified in melanoma cells. 6 out of 7 identified melanocyte identity markers are highlighted (blue). C. Boxplot of log2 intensities of all proteins compared to 6 out of 7 identified melanocytic markers (Belote et al., 2021). D. Principal component analysis of dermal and epidermal melanoma cells which

differentiate on PC1 irrelevant of the target channel used. E. Differential protein expression between epidermal (left) and dermal (right) melanoma cells. Significant proteins (t-test, q-val < 0.01) with a log2 fold change >1 (red) and <1 (blue), respectively. F. Overrepresentation analysis of significantly enriched proteins in dermal melanoma using the Wikipathway database. Color represents FDR (threshold <0.05).

Minor issues identified:

4. The numbers reported in the Derks et al paper for multiplexed bulk cell analyses used 1-hour active gradients on an Orbitrap instrument and quantified ~8,000 proteins in each sample. This should be noted in the text as the numbers reported are very similar to this paper by Thielert.

There is also an entire paragraph on comparable IDs for label-free, single and multiple channel IDs making a point that dimethyl labeling does not impact the overall IDs but that the timsTOF is beneficial for deconvolution of multiplexed spectra in complex samples. While this is certainly true, this also has been reported previously by Derks et al. in great detail for the mTRAQ reagents. Again, at a minimum, noting that this has been reported for effectively a very similar experimental paradigm needs to be part of this paragraph.

We think that we have cited Derks et al. in this paragraph and also many times over the whole manuscript to acknowledge their work. However, we revised the text to point this out more explicitly in the mentioned paragraph.

"In addition to our initially designed dia-PASEF method, we generated an alternative method, consisting of multiple MS1 scans in between dia-PASEF scans of each duty cycle (Fig EV4B), ***similar to what was performed previously on Orbitrap instruments (Derks et al, 2022).***"

5. While it is correct that Derks et al. reported ca. 1000 proteins/cell at single cell level using triplex DIA, they only used a 5 min and a 15 min. effective gradient vs. the longer gradient used here. This needs to be made clear in the text.

We have not fully compared our data to the Derks et al. paper since we also compared to the 1000 proteins/cell identified in our label-free single cell manuscript. However, we have added the used 30 min active gradient into the text.

"Applied to single cells, their 'plexDIA' approach reached a depth of 1,000 proteins per cell ***in an active gradient of 30 min.****"*

6. In the Discussion: "We also explored the idea of a reference channel in single-cell mDIA. Note that this is conceptually different from the booster channel employed in the SCoPE-MS method (Budnik et al, 2018) because the fragments of the mDIA channels are offset from each other and do not contribute to a common low-reporter mass." This point is already made very clear in the Derks paper and should be cited.

Reviewer 1 and 3 commends us for the novelty of the reference channel. In fact, the Derks et al. paper does not explore the concept of a reference channel, which in any case arguably goes back to the super-SILAC concept (although not in the DIA context). However, we have added the citation to the discussion section.

7. Figure 4 and elsewhere: "Target 4" and "Target 8" are unclear - you mean the target delta 4 and target delta 8 channels - replace.

We have renamed all statements of 'target 4' and 'target 8' to 'target Δ4' (or target d4) and 'target Δ8' (target d8), respectively. Thank you for pointing this out and improving the understanding of our introduced labels.

8. Abstract - "We demonstrate automated and complete dimethyl labeling of bulk or single-cell samples, without losing proteomic depth." You then go on and state: "In single runs of mammalian cells, a three-plex analysis of tryptic peptides quantified 7,700 proteins per channel." The wording is potentially misleading as readers may assume that the second sentence refers to single cell analyses. Be specific that the single runs are for bulk cells, not single cells.

We acknowledge that this sentence was indeed misleading as reviewer 1 also remarked. As we revised the text in the abstract, we have removed this statement (see page 1 of revised manuscript).

9. Abstract: you state "...confidently quantifies close to 4,000 proteins in single cells with excellent reproducibility" This reviewer is struggling to see where this claim is supported in the results presented. Figure 4C shows at best 3000 proteins identified while Figure 6 shows a median of ca. 2400 proteins with a few cells having ca. 4000. The median value and the interquartile range should be reported, not results for a few outliers.

This point has likewise been raised by reviewer 1 and 3, and is addressed in response to reviewer 1, point 1 (page 4).
We can understand the raised concerns. We have limited our claims for the single-cell proteomics to the median of 2,400 protein groups per cell in the revised manuscript. We hope that this clarifies your concern.

"Our algorithm RefQuant takes advantage of this and confidently quantifies twice as many proteins per single cell compared to our previous work (Brunner et al., PMID 35226415),…"

Reviewer #3:

Thielert et al.combine several known approaches in mass spectrometry (MS) and liquid chromatography (LC) to present a pipeline with increased sensitivity for single cell proteomics.

They couple multiplex data independent acquisition (mDIA) with dimethyl labeling and either trypsin or LysN digestion for 3plex or 5plex measurement respectively. The availability of multiple channels means that one of these can be designated as a reference channel to improve detection sensitivity, which is important for single cell data. In terms of LC, they use the EvoSep system which allows for low-flow separation with pre-made gradients and very small dead volume, all of which are also beneficial for single-cell analysis. After benchmarking they apply their 3plex approach in an automated fashion to about 500 single cells. They detect a median of about 2400 proteins per cell (about double what they previously reported for a similar sample) and report a throughput of 80 cells/day. They show, as they previously described at lower proteome coverage, that the intercellular variation in protein levels is smaller than the variation in transcript levels.

The study is technically sound and in the interesting area of methods development for single-cell proteomics; tech developments are needed in this area if it is to become as useful and widespread as other single cell omics measurements. Nevertheless, the paper is also very incremental, technical, and likely of interest to a specialized readership implementing or wishing to implement such approaches.

We thank the reviewer for the accurate and positive summary of our paper. As already stated above, we also agree that multiplexed-DIA will be a fruitful and powerful area for the community.

Major points

1. Novelty is overall low. Multiplex DIA applied to single cell proteomics is not in itself new (Budnik et al, 2018 PMID: 30343672), nor is dimethyl labeling. The appropriate references are cited. The minor elements of novelty in this manuscript are:

○ The combination of multiplexDIA and dimethyl labeling; the study shows that this combination works, but this is not at all surprising.
○ The use of LysN for 5 plex measurements, but this is only shown on test data
○ The use of the EvoSep system for the LC; again, it is not surprising that this works
○ The use of a reference channel in multiplexDIA, see point 2
○ The RefQuant analysis approach for quantification
○

We appreciate the reviewers' opinion that we have adequately cited the literature, which was an important point for us. We also appreciate the point about five-plexing using LysN, which we further expanded on in the revision (see also response to reviewer 1, point 5, page 8 and 9).

Regarding overall novelty, a very similar sentiment was raised by reviewer 2 and we have answered most of the points there (Reviewer 2, point 1, page 26). Furthermore, we hope

that the addition of the new use case of spatial, single cell type resolved proteomics (in response to reviewer 2, point 3, page 28-31), also helps to add novelty to the manuscript.

2. Given the multiplex nature of the data acquisition, one channel can be designated as a reference channel. Since more material can be run in this channel, the resulting robust identifications can be used to improve identification in the low-amount single-cell channels. This is conceptually not new, it was previously described in Budnik et al, but its actual implementation is new in this work.

To avoid false positives, the authors recommend an empirical threshold, but they also suggest that this reference approach may be applicable to generic proteomics studies. But at higher reference channel load, the chance of false positives may increase and a more stringent threshold may be required. If the authors wish to suggest that this may be a general approach, they should test the generality of the recommended threshold, or discuss that this "channel-q-value" threshold should be carefully re-examined for each experimental setup.

It is indeed true that the cut-off presented in the originally submitted manuscript was determined empirically. To answer the concern that these cut-offs may not be transferable to other studies, we now added same count-based FDR experiment on another species and sample of mouse tissue liver. This has been discussed and analyzed with new data in great detail in response to several questions of review 1 (see point 7, page 11). Please find the new text there or in the revised manuscript.

3. The authors claim that their approach quantitatively detects up to 4000 proteins per cell, this claim is even made in the abstract. But this is true only for a few cells (Fig 6A). The authors are clear in the text and indeed in the figure that the median is about 2400 proteins per cell, and this would be the more appropriate statement in the abstract. Also, the statement about quantifying 7,700 proteins per channel, while technically correct, is a bit misleading in the abstract since it is the per-cell number that most readers will be looking for and it would be easy to confuse this.

Apologies for the confusion. In the revised manuscript, we have limited our claims for single-cell proteomics to the median of 2,400 protein groups per cell. Additionally, we have revised the abstract and removed the sentence with 7,700 proteins per channel (see page 2 of revised manuscript). We hope that this clarifies your concern.

These remarks are similar to points made by reviewers 1 and 2 and are answered in more detail there (reviewer #1 point 1, page 4).

4. Which element of the described approach contributes most substantially to sensitivity gains? The authors state on page 12 that this is due almost entirely to the reference channel, but it is not quite clear on what this is based since there have apparently been several changes to the pipeline in comparison to their previous work. A

clear demonstration of where the improved sensitivity is coming from would benefit readers and potential users.

We tried to address the impact of the reference channel with an additional experiment in which we measured single cell equivalents with and without a reference channel. In the sample without the reference channel, we added label-free Hela of 10ng (same amount as the reference channel) to show that it is not of the higher amount injected into the LC-MS (see reviewer #2 point 2).

5. For the single-cell experiment shown in Fig 6A, the authors should report the number of peptides detected as well, and not just the number of proteins.

We agree and have added a supplementary figure in the revised manuscript of numbers of peptides and precursors from the single cells (**Response Figure 17**, new **Figure EV9B and C**).

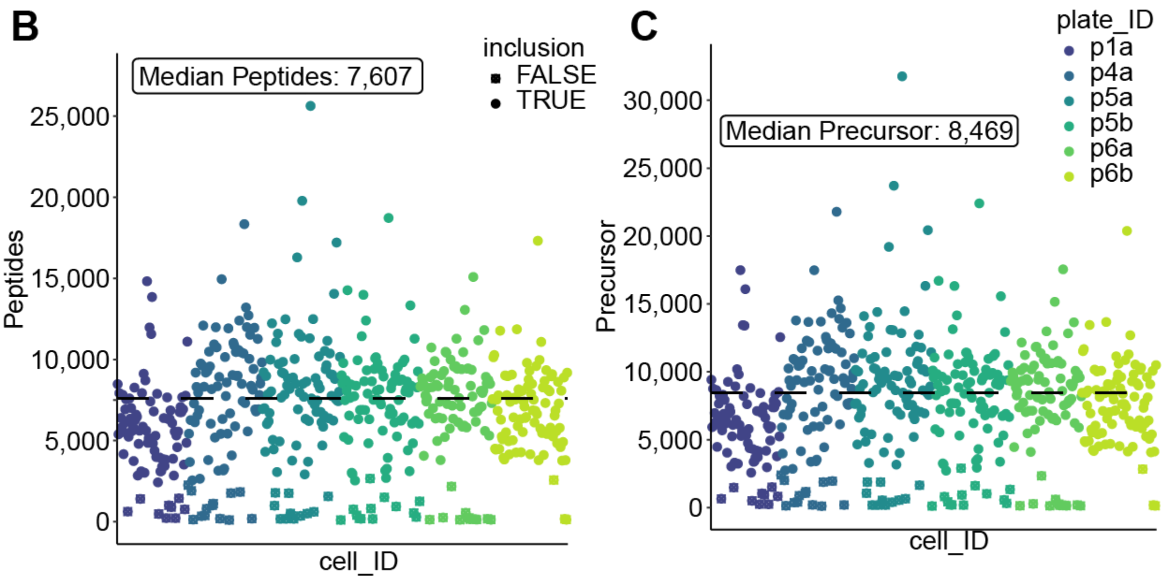

Response Figure 19, new Fig EV9B and C: A. Peptides and B. precursor identifications of single cells across different plates and runs.

"However, even at this stage, we already identified 2,377 protein groups ***and 7,607 peptides per single cell*** *and reached almost 4,000 protein groups in a few single cells (disregarding a single outlier at 4,600 identifications) (Fig 6A,* ***Fig EV9B and C****)."*

6. The authors go back and forth between describing a 3plex and a 5plex approach, the reason for this is not clear and makes the manuscript more difficult to read. Why did they not use their 5plex method for the actual single-cell experiment? Also, did they develop a different analytical pipeline for the 5plex approach?

Our intention was to present all work related to bulk material in the first part of the manuscript and then continue with the single cell part.

We wanted to emphasize that the analytical pipeline for 5-plex and 3-plex is the same, with the only difference that the LC gradient and the dia-PASEF methods were optimized for the different setups (as two different proteases were used and elution profile of peptides and their m/z to ion mobility distributions are different). All raw data was processed using DIA-NN, specifying either 3 or 5 channels. However, LysN is not fully optimized in search algorithms leading to lower numbers overall. Therefore, we decided to show the single cell work on trypsin/LysC.

To better guide the reader through this quite substantial and complex manuscript, we have revised the paragraph at the end of the introduction, that lays out the flow of analyses and applications to come in the rest of the paper (page 4 of revised manuscript).

7. The authors argue that their data supports a "stable proteome", based on the fact that inter-cell variability in protein levels is smaller than in transcript levels. This is not entirely new; they have reported it before based on data with half the median number of proteins quantified per cell. The observation also feels a bit incremental, because it is still only a subset of proteins and the data come from only about 500 cells.

Also, while this is an interesting observation, its meaning is not clear. It could be that proteins are on average present at many higher copies per cell than transcripts, so that variability in measuring the latter is more likely, or it could be because regulation of transcript levels is more prevalent than of protein levels, or there could be other reasons as well. In any case, this observation is not further developed in the manuscript, which reduces novelty and impact of this aspect of the study.

We understand the concerns of the reviewer but somewhat disagree. In fact, one of the strongest take home messages of our previous paper (Brunner et al.) was the notion of the stable core proteome and this took the transcriptomic community by surprise. While the biological reason for this is plausible, the main criticism we got was that we had only shown this for the most expressed proteins. Showing this at double the proteome depth is therefore an important point in our opinion.

We agree that this picture does not show the full recovery and the comparison of transcripts and proteomics alone. However, this has been a major concern and question in our recent study. Therefore, we wanted to show that this claim also holds true at the double depth as shown before. The meaning is that transcripts are more stochastic and in lower copy number in the cell which makes it more variable.

Minor Points

8. Are the authors sure they always have single cells in their sorted material and are not in some cases detecting doublets? How do they ensure this?

We have added FACS data to the revised manuscript and uploaded the data. Additionally, we plotted the forward side height (FSC-H) versus the forward scatter area (FSC-A) to show that this is linear. This shows that we sort single cases and do not detect doublets (also see reviewer 1, point 9).

9. The titles of Supp Figures 2 and 4 are identical.

The title of Supplementary Figures 2 and 4 are changed now. Thank you for pointing this out.

"Figure EV2 –Labeling efficiency for tryptic and Lys-N-derived HeLa peptides.

Figure EV4 - Optimal dia-PASEF acquisition method for dimethyl labeled peptides for tryptic and Lys-N-derived HeLa peptides."

10. Regarding the RefQuant approach, there seems to be an inconsistency regarding the ratio filtering step: In the method, the authors state that "the ratios are sorted in ascending order and the first 40% of ratios are retained." Which means, to my understanding, that the *smallest* 40% of the ratios are kept, correct? Which makes sense if one assumes that higher ratios would indeed only be caused by (increasing) interference signals for other fragments. However, in the main text (p 10), it is stated that the best ratio is estimated by "taking the mean from the 40% upper quantile of ratios". Shouldn't it be the 40% *lower* quantile of ratios as stated in the method instead? Please clarify.

Sorry for the confusion, indeed this is the lower quantile of ratios. We corrected this in the text.

"Subsequently, a best ratio R is estimated from the ratio distribution in a robust manner by taking the mean from the 40% lower quantile of ratios."

4th Jul 2023

Manuscript Number: MSB-2022-11503R
Title: Robust dimethyl-based multiplex-DIA doubles single-cell proteome depth via a reference channel

Dear Matthias,

Thank you for sending us your revised manuscript. We have now heard back from reviewer #1 (same reviewer who also evaluated the initial submission) who agreed to evaluate your revised study. Unfortunately, reviewers #2 and #3 were not available for reviewing the revised study. As you will see below, reviewer #1 thinks that the study has substantially improved as a result of the performed revisions. They do however raise some remaining concerns, which we would ask you to address in a minor revision. These comments seem straightforward to address but please let me know if there is anything you would like to discuss in further detail.

We would also ask you to address some remaining editorial issues listed below.

- Our data editors have noticed some unclear or missing information in the figure legends, please see the attached .doc file. Please make all requested text changes using the attached file and *keeping the "track changes" mode* so that we can easily access the edits made.

- Please provide a .doc file for the manuscript text (including legends for the main figures) and individual production-quality files for the main figures (one file per figure).

- We have replaced Supplementary Information by the Expanded View (EV format). In this case, all additional figures and Tables can be included in a PDF called Appendix. Appendix figures and Tables should be labeled and called out as: "Appendix Figure S1, Appendix Figure S2... Appendix Table S1..." etc. Each legend should be below the corresponding Figure/Table in the Appendix. Please include a Table of Contents in the beginning of the Appendix. For detailed instructions regarding expanded view please refer to our Author Guidelines: .

- Supplementary Tables should be provided as Appendix Tables and included in the Appendix File. They should be labelled and called out as Appendix Table S1, Appendix Table S2 etc. If they are longer than one page and/or more complex they should be provided as EV Datasets.

- Please include the information regarding author contributions in our submission system using CRediT.

- Please include callouts to Fig. 6C, 7F, EV1A-B, EV5G. The callout for Fig. EV3C should be after EV3B, and for EV6B after EV6A (*please note that the callouts to EV Figures should be updated to Appendix Figures!)

- Please provide a "standfirst text" summarizing the study in one or two sentences (approximately 250 characters), three to four "bullet points" highlighting the main findings and a "synopsis image" (exactly 550px width and max 400px height, jpeg or png format) to highlight the paper on our homepage.

- Supplemental text EV8 should be included in the Materials and Methods or in the Appendix (as Appendix Text 1).

Please resubmit your revised manuscript online, with a covering letter listing amendments and responses to each point raised by the referees. Please resubmit the paper **within one month** and ideally as soon as possible. If we do not receive the revised manuscript within this time period, the file might be closed and any subsequent resubmission would be treated as a new manuscript. Please use the Manuscript Number (above) in all correspondence.

Click on the link below to submit your revised paper.

https://msb.msubmit.net/cgi-bin/main.plex

As a matter of course, please make sure that you have correctly followed the instructions for authors as given on the submission website.

Thank you for submitting this paper to Molecular Systems Biology.

Kind regards,

Maria

Maria Polychronidou, PhD
Senior Editor
Molecular Systems Biology

If you do choose to resubmit, please click on the link below to submit the revision online before 3rd Aug 2023.

https://msb.msubmit.net/cgi-bin/main.plex

IMPORTANT: When you send your revision, we will require the following items:

Please note that corresponding authors are required to supply an ORCID ID for their name upon submission of a revised manuscript (EMBO Press signed a joint statement to encourage ORCID adoption).
(https://www.embopress.org/page/journal/17444292/authorguide#editorialprocess)
Currently, our records indicate that the ORCID for your account is 0000-0003-1292-4799.

Please click the link below to modify this ORCID:
Link Not Available

The system will prompt you to fill in your funding and payment information. This will allow Wiley to send you a quote for the article processing charge (APC) in case of acceptance. This quote takes into account any reduction or fee waivers that you may be eligible for. Authors do not need to pay any fees before their manuscript is accepted and transferred to the publisher.

As a matter of course, please make sure that you have correctly followed the instructions for authors as given on the submission website.

*** PLEASE NOTE *** As part of the EMBO Press transparent editorial process initiative (see our Editorial at https://dx.doi.org/10.1038/msb.2010.72 , Molecular Systems Biology will publish online a Review Process File to accompany accepted manuscripts. When preparing your letter of response, please be aware that in the event of acceptance, your cover letter/point-by-point document will be included as part of this File, which will be available to the scientific community. More information about this initiative is available in our Instructions to Authors. If you have any questions about this initiative, please contact the editorial office (msb@embo.org).

Reviewer #1:

The authors, in their revised manuscript, have gone through substantial efforts to improve their work and to address the concerns from the referees. In most cases, they have successfully done so and made great steps forward towards their work being suitable for publication. Adding the biological application through DVP-based analysis is an elegant addition that showcases the improved impact of their workflow (albeit conceptually already pre-printed in https://www.biorxiv.org/content/10.1101/2022.12.03.518957v1 as well). The added details regarding how the data acquisition and searches were done, spectral library and clarifications regarding which datasets (scXXX) were generated certainly enhance the interpretability of their work.

That said, we and the other referees had substantial concerns about the technical aspects of the workflow, and as it stands, the improvements with regards to the quality of quantification appear to be primarily cosmetic. Thus, we would like to urge the authors to better address our remaining concerns regarding the wide ratio distributions and biases. Please allow me to clarify in more detail:

Main Concerns:

1. Our main concern is that the authors have insufficiently investigated the effect of the amount in the reference channel on the quantitative accuracy. The current point of reference in Fig. 5C indicates that the measured ratios are distributed around the true ratios and that there is no systematic ratio compression. However this experiment was only performed at 10ng reference, so we don´t know how wide the distributions would be for lower reference amounts. Additionally, how did the authors arrive at 10ng being the optimal amount for adding protein IDs without sacrificing quantification accuracy and precision?
Another experiment is shown in Fig. EV7. Why did the authors use MS1 quantities for this experiment? Is it still considered as RefQuant?
In this experiment, correlations of protein abundances between single-cell equivalents measured at different reference levels were calculated. The results indicate a decrease in correlations when comparing the "low ref" vs. the "high ref". but also a decrease in correlations comparing the "high ref" with itself. E.g. correlations between Ref 10ng d8 and Ref 10ng d4 are lower

than e.g. correlations between Ref 1.25ng d8 vs. Ref 125ng d4. This indicates that measurements with a 10ng reference channel are less reproducible than measurements with 1.25ng reference (assuming that the scatterplots show the same number of datapoints).
These results indicate a large influence of the reference channel on quantification, especially since these scatterplots are based on absolute protein quantities and not fold changes. These correlations do not measure quantitative accuracy as discussed in Fig. 2 here: https://www.nature.com/articles/s41592-023-01785-3
In this example, T-cells and monocytes have an overall protein abundance correlation of 0.95 (!) despite being very different cell types, making the argument that correlating abundances between proteomes does not in fact measure quantitative accuracy.

Thus, if Fig. 5C was created with a 5ng/2.5ng/... reference, I would expect narrower distributions around the expected ratio, even when the same precursors are compared. Therefore I expect that increasing the reference channel improves proteome coverage but comes at the cost of overall quantification accuracy, as the reference channel itself adds interfering ions.

There are other ways to investigate this:
e.g. measuring ratios between multi-species mixes at different reference levels or comparing fold-changes between 2 cell lines in the 2 target channels measured with different reference levels to bulk measured fold-changes. It is important for the authors to investigate this effect in more detail, as the community needs to know the tradeoffs of such high reference channel amounts and how to assess its impact quantitatively.

2. The authors claim that the large increase in CV values was due to the use "Precursors.Translated", which potentially does not give accurate values due retention time shifts caused by deuterium. Could the authors substantiate this claim, by e.g. checking the specific retention times to empirically determine if that is the case?

3. Figure 2E. The Boxplot with the different species mix are much wider compared to plexDIA (Derks et al, 2022), does this indicate a lower quantification quality compared to a reference-channel-free plexDIA approach?

4. In their revised manuscript, the authors switched to 'Precursor.Normalized', but 'Precursors.Translated' is still used for single-cell analysis. Could they explain why the different quantification methods are used for the respective use-cases and how the results by using 'Precursor.Normalized' are affected?

5. Regarding the substantial dropout rate of single-cell measurements, I would encourage the authors to more clearly discuss this in the manuscript. As it stands, their results suggest their workflow to actually perform at almost half the effective throughput. It could be that it is purely a FACS issue, but given the extra sample handling steps introduced both by labelling and subsequent transfer into an EvoTip, this aspect should at the very least be discussed in more detail.

Minor Concerns:

6. In the main text, the authors still tend to underrepresent certain issues present in the shown data:
e.g. in Figure 1C there is quite a clear off-set from the true ratios, but in the text, the authors describe the quantification accuracy as "excellent". While I agree that the quant is sufficiently accurate, a slight tempering of their claim would be justified.

7. For Figure 2, it is apparent that the number of identified peptides decreases with the addition of extra channels, up to as much as ~30% in the 3-plex, however in the text the authors claim that there is no negative impact. This should be more correctly described in the results text.

8. While the authors have tried to be more modest in their claims, there are still some statements that come across as exaggerated. E.g. "twice as many proteins per single cell compared to our previous work (Brunner et al.)". This is true if one compares their current median performance against the G1 cells from said previous work (~1200 proteins per cell), where the other populations reached 1700-2000 protein groups per single cell. As the mDIA workflow now reaches a median number of 2500, it is not double the depth irrespective of cell type, and likely that G1 cells in this work would also be well below the median proteome coverage.

9. For the mDIA-DVP part, the authors claim that they have increased their throughput 28-fold. How exactly this number was derived is unclear, as in MS terms, running samples as a multiplex of 2 with a decreased runtime of 2 would lead to a 4-fold improvement? It is unclear how the cutting of "shapes" can account for an increased throughput in the context of mDIA (as sample prep is done in parallel to MS analysis), and in my opinion, should be presented separately.

10. Misplaced commas in Figure 3B (5-plex panel) and missing commas in 3C (5-plex panel)

11. The authors should adjust their FWHM claim to 3s if they want to round the number to an integer, as 2.7s should then be rounded up, not down.

12. Citations that are still missing are:

I) Xuan et al, 2020 (for MS1 scan insertion between MS2s)
II) Matzinger et al, 2023 (label-free single-cell proteomics)
III) Specht et al., 2021 (384-well plate prep for SCP, pg. 3)

Point-by-point response to reviewer comments for “Robust dimethyl-based multiplex-DIA doubles single-cell proteome depth via a reference channel”.

We thank the editor and reviewer 1 for providing us with detailed and valuable feedback to our revised manuscript. We thank the reviewer for acknowledging our ‘substantial efforts’ to improve our manuscript by addressing their concerns. In light of the editor’s timeline (returning the manuscript as soon as possible but within four weeks), we have addressed the central remaining concern of the reviewer by a number of new analyses. These deal with the quantitative accuracy in regards to the reference channel amounts, from single cell equivalent (0.25 ng) to the standard amount used in the single cell part of the paper (10 ng). This confirmed that the reference channel amount has little if any impact on quantitative accuracy when using RefQuant.
Furthermore, we addressed all remaining points as best we could.

We used following color code:
black: written comments by the reviewers
blue: our explanation
green: modified text in the revised manuscript

Reviewer #1:

The authors, in their revised manuscript, have gone through substantial efforts to improve their work and to address the concerns from the referees. In most cases, they have successfully done so and made great steps forward towards their work being suitable for publication. Adding the biological application through DVP-based analysis is an elegant addition that showcases the improved impact of their workflow (albeit conceptually already pre-printed in https://www.biorxiv.org/content/10.1101/2022.12.03.518957v1 as well). The added details regarding how the data acquisition and searches were done, spectral library and clarifications regarding which datasets (scXXX) were generated certainly enhance the interpretability of their work.

We thank the reviewer for their positive assessment of our effort to improve the work and manuscript. We appreciate the constructive and detailed comments.
Regarding the ‘single shape’ preprint from our group that the reviewer refers to: that is actually a follow up paper to this one (which has taken longer than anticipated to be published). Importantly, the biological application is not at all the same. The preprint deals with single shapes in fresh frozen mouse hepatocytes from tissues whereas the application here is on melanoma in humans from 2.5 um FFPE tissue, comparing relatively subtle differences between epidermal with dermal cancer cells.

That said, we and the other referees had substantial concerns about the technical aspects of the workflow, and as it stands, the improvements with regards to the quality of quantification appear to be primarily cosmetic. Thus, we would like to urge the authors to better address our remaining concerns regarding the wide ratio distributions and biases. Please allow me to clarify in more detail:

We disagree that our substantial work in the first revision is 'merely cosmetic'. In fact it took a whole group of people many month to do the new experiments, software and analyses. We also believe that the reviewer's comments did make the manuscript substantially and not cosmetically better. Below, we do our best to address the remaining concerns of this reviewer.

Main Concerns:

1. Our main concern is that the authors have insufficiently investigated the effect of the amount in the reference channel on the quantitative accuracy. The current point of reference in Fig. 5C indicates that the measured ratios are distributed around the true ratios and that there is no systematic ratio compression. However this experiment was only performed at 10ng reference, so we don´t know how wide the distributions would be for lower reference amounts.

We thank the reviewer for highlighting the importance of this issue and we address this below (Repsonse Figure 1 and new Appendix Figure S4A-C). Our additional test indicate that – given appropriate computational processing of the data – there is no substantial decrease in quantitative performance due to a high amount of peptides in the reference channel in mDIA.
As stated by the reviewer Fig. 5C indicates that ratio compression is low and we believe that this plot is a rigorous test for ratio compression (even at 10 ng reference channel). Furthermore, Fig. 5B is also a rigorous test for ratio compression, as we have a ground truth of the known ratio between target and reference channel. The figure confirms that peptide precursors (which are much more challenging to quantify than proteins) are properly distributed around this ratio. We had only included the 10 ng condition in this plot, as this is the condition we subsequently use in the study. To address the question of the reviewer on how wide the distributions around the ground truth would be for different reference amounts, we have now carried out additional analyses where we check for ratio compression and distribution width for different reference channel amounts. We describe this as follows in the revised main text:

"Next, we specifically investigated the effects of varying the peptide amount in the reference channel with or without RefQuant. We analyzed the ratios to reference over a range from single cell equivalents (0.25 ng) to 10 ng in the reference channel (Appendix Fig S4A-C). Without RefQuant, we observed a clear dependence of the distribution spread (measured via the standard deviation) on the amount of reference channel at the MS1 level. This spread was substantially reduced on the MS2 level (assessed via 'Precursor.Translated' and the 'Precursor.Normalised' quantities from DIA-NN). However, when applying RefQuant to the same data virtually no dependence of the standard deviation on reference channel amount was observed. Additionally,

only RefQuant eliminated systematic ratio compression as long as the reference channel was dominant. This demonstrates that RefQuant specifically utilizes the information of the reference channel and thereby removes potential artifacts."

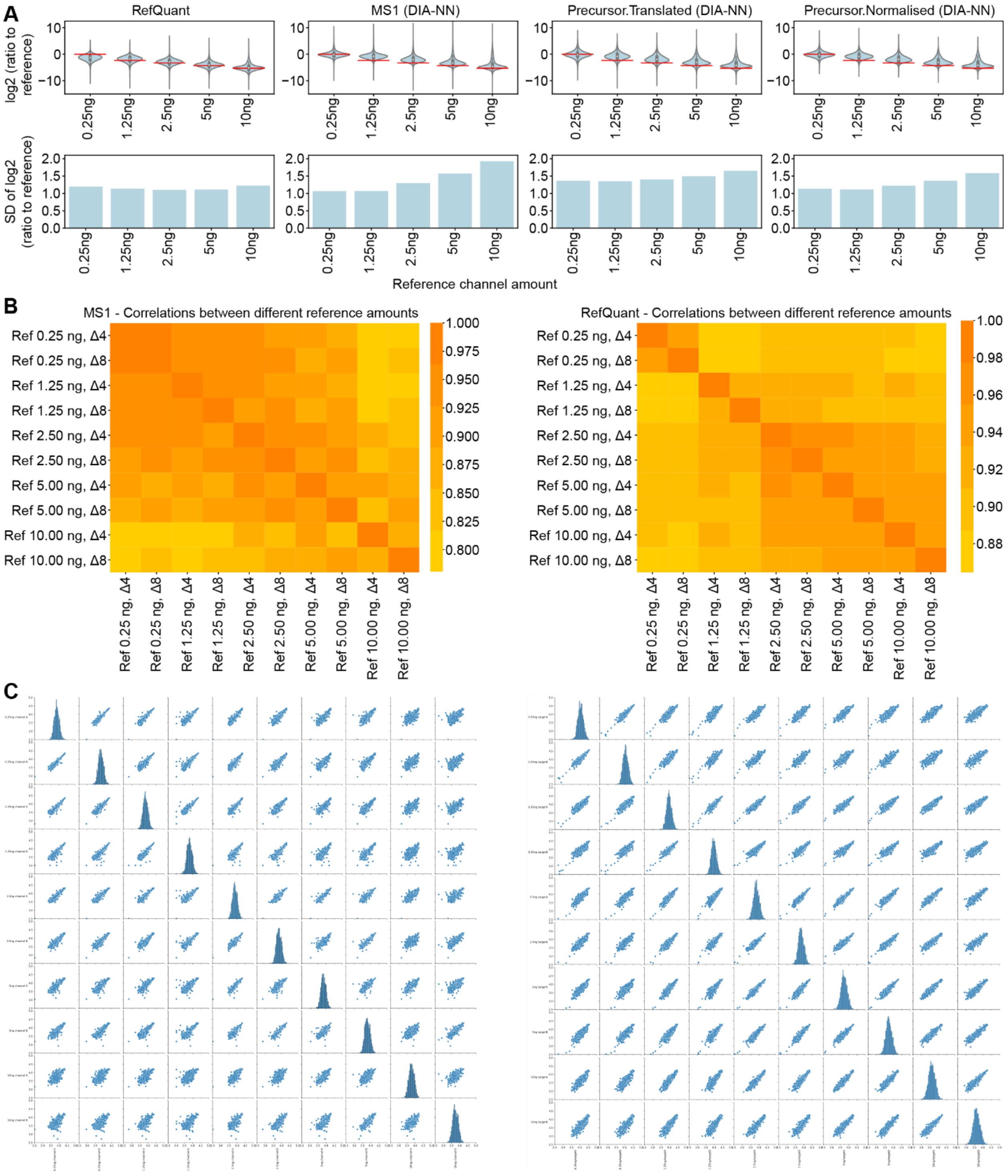

Response Figure 1, new Appendix Figure S4 - Quantitative accuracy and reproducibly of target channels in dependence of the reference channel amount using the scReference dataset.

A. Comparison of the observed ratios to reference to the known ground truth ratio (scReference dataset). Top: distributions as violin plots, bottom: distribution width quantified via the standard deviation. Dependence on amount of reference channel is observed most strongly for MS1 data and RefQuant eliminates most biases. RefQuant under-estimates the ratios in the 0.25ng case, as the model assumptions of RefQuant rely on having a more abundant reference channel. It is not applicable to non-reference channel settings.

B and C. Quantification accuracy in Pearson correlation coefficients (B) or quantity comparisons (C) between single cell equivalents in target channels (Δ4 and Δ8) dependent on different reference channel amounts (0.25ng to 10ng) using the scReference dataset on unmodified MS1 quantities (left) and RefQuant quantities (right). Correlations are improved by using RefQuant and effects of reference channel amounts are mitigated.

Additionally, how did the authors arrive at 10ng being the optimal amount for adding protein IDs without sacrificing quantification accuracy and precision?

This is due to our experience with the Bruker timsTOF SCP, which is a very sensitive instrument. As evaluated in Appendix Figure 5A, using 10 ng, results in a substantial coverage of the proteome; certainly sufficient to cover the peptides expected from single cells or single cell equivalents. As explained just above, there is no substantial tradeoff in quantification accuracy and precision due to RefQuant. That said, looking at Figure 4C less reference channel (as for example 5 ng, 20x) could be beneficial as well, perhaps on instruments with automatic gain control such as Thermo instruments.

Another experiment is shown in Fig. EV7. Why did the authors use MS1 quantities for this experiment? Is it still considered as RefQuant?

We chose to show the MS1 quantities in this case because we wanted to stay as close to the raw data as possible in order to detect possible interferences. As now also shown in Appendix Figure S4A-C (see Response Figure 1), RefQuant is able to efficiently deal with such artifacts. Therefore, when using RefQuant quantities, these effects are not present. We think it is important to show both types of quantities to highlight potential pitfalls. To clarify this, we added the following sentence to the revised manuscript.

*“The Pearson correlations were always greater than **0.88 using RefQuant or** 0.8 **for MS1 quantities** and did not differ between target channels Δ4 and Δ8 (Appendix Fig S4B).”*

In this experiment, correlations of protein abundances between single-cell equivalents measured at different reference levels were calculated. The results indicate a decrease in correlations when comparing the "low ref" vs. the "high ref". but also a decrease in correlations comparing the "high ref" with itself. E.g. correlations between Ref 10ng d8 and Ref 10ng d4 are lower than e.g. correlations between Ref 1.25ng d8 vs. Ref 125ng d4. This indicates that measurements with a

10ng reference channel are less reproducible than measurements with 1.25ng reference (assuming that the scatterplots show the same number of datapoints). These results indicate a large influence of the reference channel on quantification, especially since these scatterplots are based on absolute protein quantities and not fold changes.

These correlations do not measure quantitative accuracy as discussed in Fig. 2 here: https://www.nature.com/articles/s41592-023-01785-3
In this example, T-cells and monocytes have an overall protein abundance correlation of 0.95 (!) despite being very different cell types, making the argument that correlating abundances between proteomes does not in fact measure quantitative accuracy.
Thus, if Fig. 5C was created with a 5ng/2.5ng/... reference, I would expect narrower distributions around the expected ratio, even when the same precursors are compared. Therefore I expect that increasing the reference channel improves proteome coverage but comes at the cost of overall quantification accuracy, as the reference channel itself adds interfering ions.

Please see our comments above. We agree with the reviewer that quantitative accuracy is improved when using a lower reference amount on the MS1 level, therefore representing a tradeoff between quantitative accuracy and proteomics depth. However, as we have shown above, these effects are most pronounced on the MS1 level and we can efficiently deal with them using RefQuant, as displayed in Appendix Figure S4A-C.
The reviewer raises an interesting point about correlations as a measure for quantitative accuracy. Indeed, correlations suffer from the drawback that the dynamic range of the quantified proteins influences the correlation. If proteins span a large dynamic range relative changes between proteins will add less noise and therefore the correlation stays high. However, this criticism mainly applies to correlation of an absolute measure of quantification quality. We agree that fold changes could be a more direct measure and we have now also included fold change-based comparisons (Response Fig 2 and new Appendix Figure S4D). Nevertheless, proteome rank order correlation is still a fair measure to compare experiments, that have been done under similar conditions and they are very established in the community. In any case, the fold change results and the correlation results are also very similar as can be seen in Appendix Figure S4A-D.

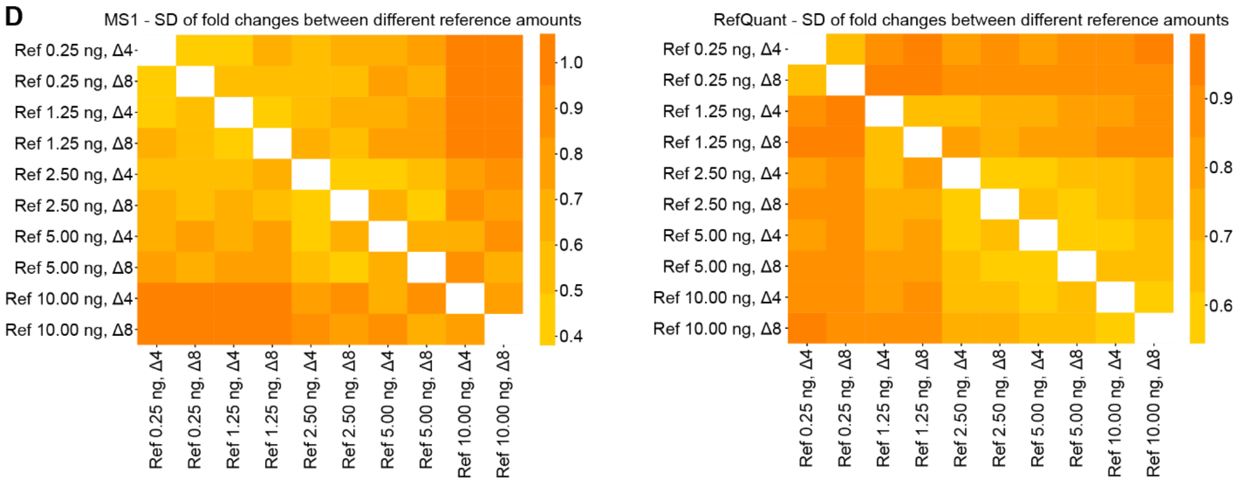

Response Figure 2, new Appendix Figure S4D - Quantitative accuracy and reproducibly of target channels in dependence of the reference channel amount using the scReference dataset.
D. As in B, but using the standard deviation of the fold changes between precursors. Quantification accuracy yields similar results.

There are other ways to investigate this:
e.g. measuring ratios between multi-species mixes at different reference levels or comparing fold-changes between 2 cell lines in the 2 target channels measured with different reference levels to bulk measured fold-changes. It is important for the authors to investigate this effect in more detail, as the community needs to know the tradeoffs of such high reference channel amounts and how to assess its impact quantitatively.

As discussed above, we have now included multiple analyses addressing the explicit concern of the reviewer about the influence of the reference channel amount on quantitative accuracy. We believe that these analyses resolve the concerns raised. Therefore, although we agree that it would be interesting to perform single cell mixed species experiment, this would almost be a separate paper if done properly and we believe it is beyond the scope of this paper, especially given the tight timeline for the second resubmission.

2. The authors claim that the large increase in CV values was due to the use "Precursors.Translated", which potentially does not give accurate values due retention time shifts caused by deuterium. Could the authors substantiate this claim, by e.g. checking the specific retention times to empirically determine if that is the case?

The retention time shift in dimethyl-labeled peptides has already been reported by Boersema et al (Nature Protocols, 2009 https://doi.org/10.1038/nprot.2009.21). This slight separation among the dimethyl channels is caused by deuterium being slightly more hydrophilic than hydrogen. This is also demonstrated in Response Figure 1A below, where the elution peak of differentially labeled species of the same peptide (LLLPGELAK) constantly shifts to the left as we go from light to heavy channels. Overall, the retention time shift reported by DIA-NN between Δ0 and Δ4 channels has a median value of 0 s for both Orbitrap and timsTOF instruments (Response Figure 1B). Meanwhile, the retention time shift between Δ0 and Δ8 channels has a median value of 3.5 s in the Orbitrap and 2.1 s in the timsTOF.

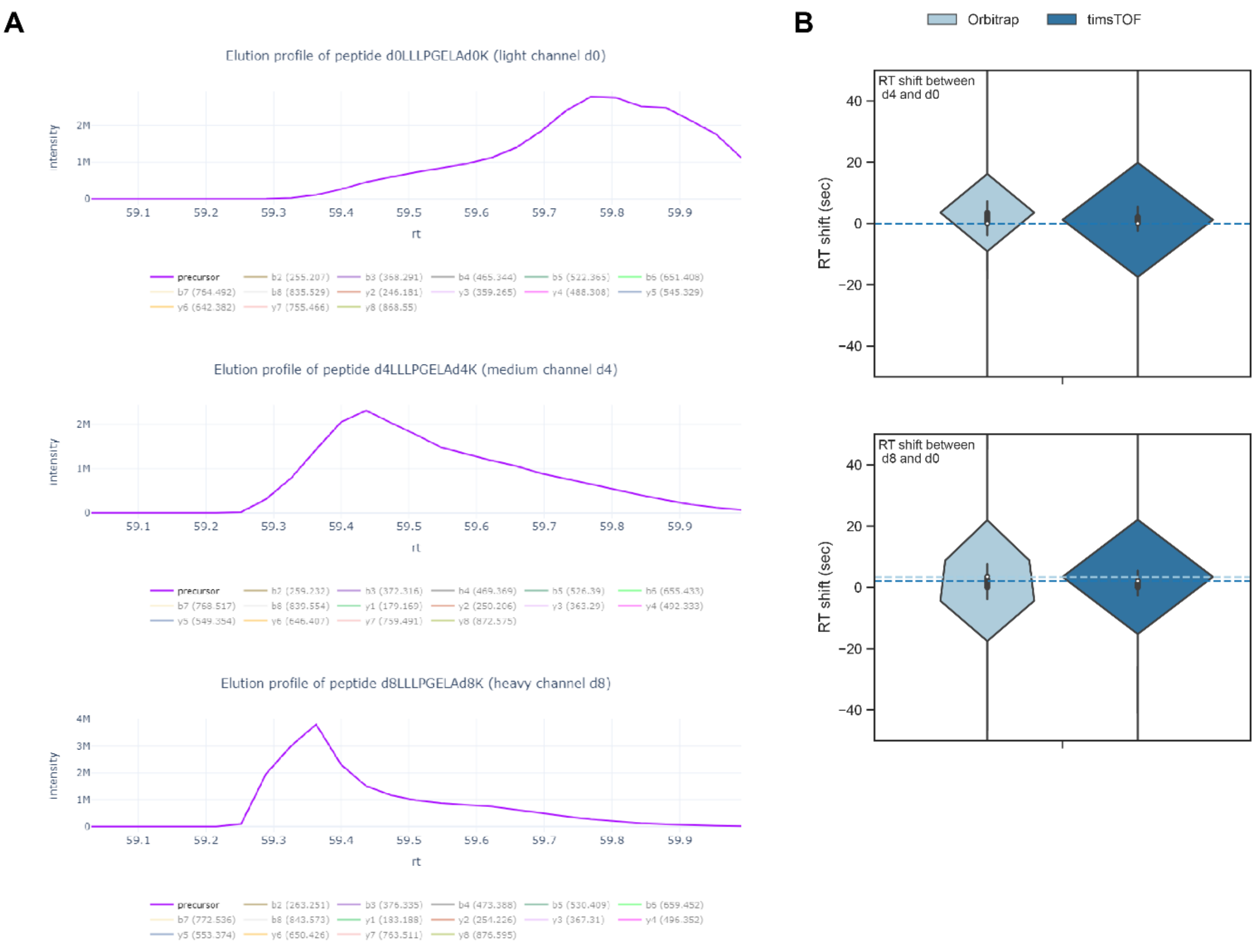

Response Figure 3. Retention time shift across channels in dimethyl-labeled peptides.
A. Elution profiles of differentially-labeled species of the LLLPGELAK peptide with charge of z = +2 (upper panel: Δ0 channel, middle panel: Δ4 channel, lower panel: Δ8 channel).
B. RT shift of all peptides in all replicates for both Orbitrap and timsTOF instruments (upper panel: RT shift between Δ0 and Δ4 channels, lower panel: RT shift between Δ0 and Δ8 channels).

3. Figure 2E. The Boxplot with the different species mix are much wider compared to plexDIA (Derks et al, 2022), does this indicate a lower quantification quality compared to a reference-channel-free plexDIA approach?

Please note that Figure 2E was not prepared in a reference channel setup. Therefore, this analysis is similar to figure 3 in the plexDIA publication. As far as we understand, those authors plotted intersected protein group ratios between plexDIA and LF-DIA which reduces the protein numbers and also low abundant proteins. Doing this intersection may be an explanation for the narrower distribution in plexDIA rather than lower quantification quality in our data. As we do not have label-free data of the same experiment, we cannot use an analogous strategy in using intersected protein groups as in plexDIA paper. As a general point, it is clear that there are still many opportunities to improve quantification on the algorithmic side, see for example the very recent preprint by Vadim Demichev for DIA-NN (https://www.biorxiv.org/content/10.1101/2023.06.20.545604v1).

4. In their revised manuscript, the authors switched to 'Precursor.Normalized', but 'Precursors.Translated' is still used for single-cell analysis. Could they explain why the different quantification methods are used for the respective use-cases and how the results by using 'Precursor.Normalized' are affected?

We thank the reviewer to point that out and agree that it is confusing that we compare RefQuant to ‘Precursor.Translated’ values in Figure 5B as we use ‘Precursor.Normalised’ in the reference channel-free setup (Fig 1-3). To clarify, we do not use ‘Precursor.Translated’ for the single-cell analysis, since we used RefQuant which directly accesses ‘raw’ intensities of fragment ions (peak intensity extractions of fragments by DIA-NN). We used ‘Precursor.Translated’ as the quantification standard against which we compared RefQuant as ‘Precursor.Translated’ was used for single cell analysis in the plexDIA paper – therefore in our opinion this is the standard to validate RefQuant. We agree that it is also interesting to compare against ‘Precursor.Normalised’ as we saw that it performs better in our (reference channel free) mixed species experiment. Therefore we revised Figure 5B with an additional panel of ‘Precursor.Normalised’.

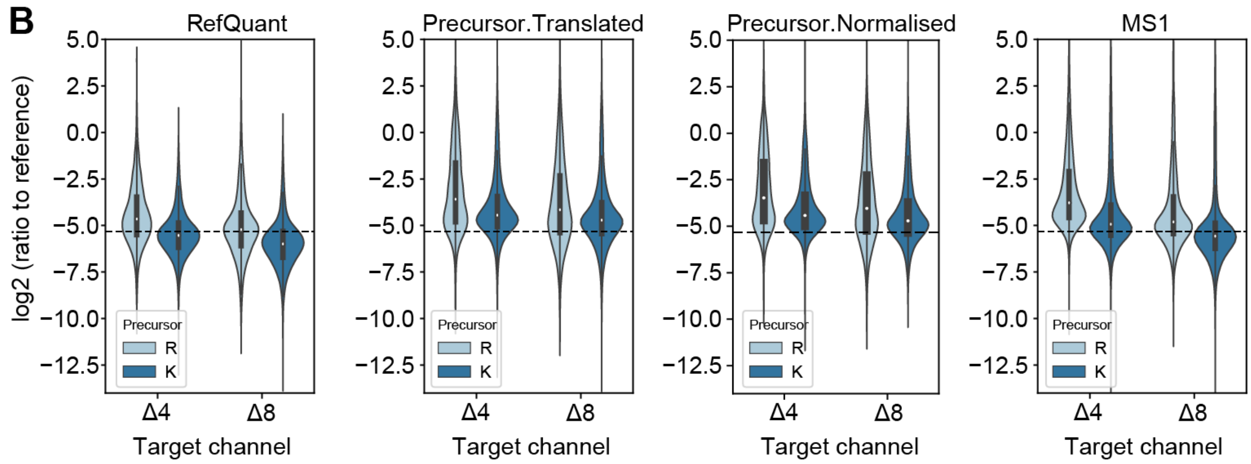

Response Figure 4, adjusted panel in Figure 5B - RefQuant quantifies single-cell equivalents accurately based on sampling ratios relative to the reference channel.
B. Ratio of reference channel to each target channel by arginine (R) and lysine (K) precursor based on quantities from RefQuant (left), Precursor.Translated and Precursor.Normalised by DIA-NN (middle) and MS1 by DIA-NN (right) on the scBenchmark dataset (technical replicates, n=5). RefQuant showed best the expected ratios in both target channels compared to DIA-NN quantities of Precursor.Translated and MS1. The violin plot shows the distribution of the data while the box depicts the interquartile range with the central band representing the median value of the dataset. The whiskers represent the furthest datapoint within 1.5 times the interquartile range (IQR).

5. Regarding the substantial dropout rate of single-cell measurements, I would encourage the authors to more clearly discuss this in the manuscript. As it stands, their results suggest their workflow to actually perform at almost half the effective throughput. It could be that it is purely a FACS issue, but given the extra sample handling steps introduced both by labelling and

subsequent transfer into an EvoTip, this aspect should at the very least be discussed in more detail.

We thank the reviewer for touching on this point. We want to highlight again that there are no additional sampling steps introduced by the labeling since it only requires to pipette the two labeling reagents into the well in which the single cell is already placed and handled. Additionally, the transfer step into an EvoTip was necessary before the labeling as well. However, we agree that the discussion might be valuable, therefore we added the following text to the single cell result section.

"We also note that FACS-sorting may lead to some drop outs which might be overcome with more sensitive sorting strategies as well as the possibility to take pictures of each isolated single cell to validate an intact cell for analysis as done in the cellenONE instrument (Hartlmayr et al, 2021). This may help to reduce drop outs and throughput."

Minor Concerns:

6. In the main text, the authors still tend to underrepresent certain issues present in the shown data:
e.g. in Figure 1C there is quite a clear off-set from the true ratios, but in the text, the authors describe the quantification accuracy as "excellent". While I agree that the quant is sufficiently accurate, a slight tempering of their claim would be justified.

We have addressed the concern of the reviewer by replacing the phrase: "This revealed **excellent** quantification accuracy…" to *"This revealed a **high degree** of quantification accuracy…".*

7. For Figure 2, it is apparent that the number of identified peptides decreases with the addition of extra channels, up to as much as ~30% in the 3-plex, however in the text the authors claim that there is no negative impact. This should be more correctly described in the results text.

Thank you for prompting us to clarify this part of the text. In the manuscript we wrote:
*"Whereas the timsTOF platform generally yielded higher identification numbers and greater quantification precision, **the number of quantified precursors and protein groups between unlabeled and one-channel dimethyl labeled samples was similar on both instruments**. This demonstrates that derivatization of peptides with dimethyl groups does not negatively impact peptide identification rates."*

As highlighted in this text, the claim of having "no negative impact" in the number of identified peptides refers to the comparison between unlabeled and one-channel dimethyl-labeled samples. Our aim was to reinforce that equal amount of both unlabeled and Δ0-labeled samples would yield a similar number of identified peptides. This serves to validate our contention that the labeling process was indeed complete, supporting the >99% labeling efficiency that we have reported and depicted in Figure 1B, Figure 4B and Figure EV2.

8. While the authors have tried to be more modest in their claims, there are still some statements that come across as exaggerated. E.g. "twice as many proteins per single cell compared to our previous work (Brunner et al.)". This is true if one compares their current median performance against the G1 cells from said previous work (~1200 proteins per cell), where the other populations reached 1700-2000 protein groups per single cell. As the mDIA workflow now reaches a median number of 2500, it is not double the depth irrespective of cell type, and likely that G1 cells in this work would also be well below the median proteome coverage.

We appreciate the reviewer's concerns but we note that we already removed our claims regarding 'up to 4000 proteins in single cells' in this revision. We stand by the fact that we identify twice as many proteins compared to the experiments reported in Brunner et al, which is also born out in our daily experience with single cell measurements. This increase is also implicit in Fig. 4C where we analyzed single cell equivalents.

9. For the mDIA-DVP part, the authors claim that they have increased their throughput 28-fold. How exactly this number was derived is unclear, as in MS terms, running samples as a multiplex of 2 with a decreased runtime of 2 would lead to a 4-fold improvement? It is unclear how the cutting of "shapes" can account for an increased throughput in the context of mDIA (as sample prep is done in parallel to MS analysis), and in my opinion, should be presented separately.

Upon reflection, we agree with the reviewer. The confusion arises because there are two potential bottle necks in this workflow; the time the laser microdissection instrument takes to cut the cells and the MS analysis. The higher sensitivity of mDIA now allows us to cut seven times less shapes whereas the improvement on the MS side is indeed four-fold. This is now directly described in the revised text, including in the abstract.

Abstract:
"Finally, we combined mDIA with spatial proteomics to increase the throughput of Deep Visual Proteomics seven-fold for microdissection and four-fold for MS analysis."

Main text:
"This represents an increased throughput of four-fold for MS analysis without losing proteomic depth and a seven-fold decreased cutting time on the laser microdissection instrument, a large gain for the overall DVP pipeline."

10. Misplaced commas in Figure 3B (5-plex panel) and missing commas in 3C (5-plex panel)

We have adapted the misplaced and missing commas.

11. The authors should adjust their FWHM claim to 3s if they want to round the number to an integer, as 2.7s should then be rounded up, not down.

Thank you for pointing this out. We have already adapted the number for FWHM in the previous revised manuscript to 2.7s in the main text:
"This setup makes the workflow very robust and reproducible, and also improved chromatographic resolution, with peaks eluting in full width half maximum (FWHM) with a median of about ***2.7 s****, corresponding to an elution volume of only 8 nL at peak width."*

12. Citations that are still missing are:

I) Xuan et al, 2020 (for MS1 scan insertion between MS2s)
II) Matzinger et al, 2023 (label-free single-cell proteomics)
III) Specht et al., 2021 (384-well plate prep for SCP, pg. 3)

We have added the suggested citations to the introduction (II, III) and result section (I).

25th Jul 2023

Manuscript number: MSB-2022-11503RR
Title: Robust dimethyl-based multiplex-DIA doubles single-cell proteome depth via a reference channel

Dear Matthias,

Thank you again for sending us your revised manuscript. We are now satisfied with the modifications made and I am pleased to inform you that your paper has been accepted for publication.

*** PLEASE NOTE *** As part of the EMBO Publications transparent editorial process initiative (see our Editorial at https://dx.doi.org/10.1038/msb.2010.72), Molecular Systems Biology publishes online a Review Process File with each accepted manuscripts. This file will be published in conjunction with your paper and will include the anonymous referee reports, your point- by-point response and all pertinent correspondence relating to the manuscript. If you do NOT want this File to be published, please inform the editorial office at msb@embo.org within 14 days upon receipt of the present letter.

Should you be planning a Press Release on your article, please get in contact with msb@wiley.com as early as possible, in order to coordinate publication and release dates.

LICENSE AND PAYMENT:
All articles published in Molecular Systems Biology are fully open access: immediately and freely available to read, download and share.

Molecular Systems Biology charges an article processing charge (APC) to cover the publication costs. You, as the corresponding author for this manuscript, should have already received a quote with the article processing fee separately. Please let us know in case this quote has not been received.

Once your article is at Wiley for editorial production you will receive an email from Wiley's Author Services system, which will ask you to log in and will present you with the publication license form for completion. Within the same system the publication fee can be paid by credit card, an invoice or pro forma can be requested.

Payment of the publication charge and the signed Open Access Agreement form must be received before the article can be published online.

Molecular Systems Biology articles are published under the Creative Commons licence CC BY, which facilitates the sharing of scientific information by reducing legal barriers, while mandating attribution of the source in accordance to standard scholarly practice.

Proofs will be forwarded to you within the next 2-3 weeks.

Thank you very much for submitting your work to Molecular Systems Biology.

Kind regards,

Maria

Maria Polychronidou, PhD
Senior Editor
Molecular Systems Biology

EMBO Press Author Checklist

Corresponding Author Name: Matthias Mann	
Journal Submitted to: Molecular System Biology	
Manuscript Number: ID MSB-2022-11503R	

USEFUL LINKS FOR COMPLETING THIS FORM
The EMBO Journal - Author Guidelines
EMBO Reports - Author Guidelines
Molecular Systems Biology - Author Guidelines
EMBO Molecular Medicine - Author Guidelines

Reporting Checklist for Life Science Articles (updated January 2022)
This checklist is adapted from Materials Design Analysis Reporting (MDAR) Checklist for Authors. MDAR establishes a minimum set of requirements in transparent reporting in the life sciences (see Statement of Task: 10.31222/osf.io/9sm4x). Please follow the journal's guidelines in preparing your manuscript.
Please note that a copy of this checklist will be published alongside your article.

Abridged guidelines for figures
1. Data
The data shown in figures should satisfy the following conditions:
- the data were obtained and processed according to the field's best practice and are presented to reflect the results of the experiments in an accurate and unbiased manner.
- ideally, figure panels should include only measurements that are directly comparable to each other and obtained with the same assay.
- plots include clearly labeled error bars for independent experiments and sample sizes. Unless justified, error bars should not be shown for technical replicates.
- if n<5, the individual data points from each experiment should be plotted. Any statistical test employed should be justified.
- Source Data should be included to report the data underlying figures according to the guidelines set out in the authorship guidelines on Data Presentation.

2. Captions
Each figure caption should contain the following information, for each panel where they are relevant:
- a specification of the experimental system investigated (eg cell line, species name).
- the assay(s) and method(s) used to carry out the reported observations and measurements.
- an explicit mention of the biological and chemical entity(ies) that are being measured.
- an explicit mention of the biological and chemical entity(ies) that are altered/varied/perturbed in a controlled manner.
- the exact sample size (n) for each experimental group/condition, given as a number, not a range;
- a description of the sample collection allowing the reader to understand whether the samples represent technical or biological replicates (including how many animals, litters, cultures, etc.).
- a statement of how many times the experiment shown was independently replicated in the laboratory.
- definitions of statistical methods and measures:
  - common tests, such as t-test (please specify whether paired vs. unpaired), simple χ2 tests, Wilcoxon and Mann-Whitney tests, can be unambiguously identified by name only, but more complex techniques should be described in the methods section;
  - are tests one-sided or two-sided?
  - are there adjustments for multiple comparisons?
  - exact statistical test results, e.g., P values = x but not P values < x;
  - definition of 'center values' as median or average;
  - definition of error bars as s.d. or s.e.m.

Please complete ALL of the questions below.
Select "Not Applicable" only when the requested information is not relevant for your study.

Materials

Newly Created Materials	Information included in the manuscript?	In which section is the information available? (Reagents and Tools Table, Materials and Methods, Figures, Data Availability Section)
New materials and reagents need to be available; do any restrictions apply?	Yes	Material & Methods; All used materials and reagents in this study are available to the public and can be bought. FFPE human tissue was provided by the University Hospital of Zurich (Switzerland) according to the institutional review board protocol.

Antibodies	Information included in the manuscript?	In which section is the information available? (Reagents and Tools Table, Materials and Methods, Figures, Data Availability Section)
For **antibodies** provide the following information:<br>- Commercial antibodies: RRID (if possible) or supplier name, catalogue number and or/clone number<br>- Non-commercial: RRID or citation	Yes	Material & Methods; primary: 1. CD44 (AB_868936, Abcam ab51037; rabbit, 1:200) , 2. SOX10 (NordicBiosite bsh-7959-1; mouse, 1:50); secondary: 1. mouse-IgG (AB_2633277, Invitrogen A32728, Alexa-647, 1:400), 2. rabbit-IgG (AB_2633281, Invitrogen A32732, Alexa-555, 1:400)

DNA and RNA sequences	Information included in the manuscript?	In which section is the information available? (Reagents and Tools Table, Materials and Methods, Figures, Data Availability Section)
**Short novel DNA or RNA including primers, probes**: provide the sequences.	Not Applicable	Not Applicable

Cell materials	Information included in the manuscript?	In which section is the information available? (Reagents and Tools Table, Materials and Methods, Figures, Data Availability Section)
**Cell lines:** Provide species information, strain. Provide accession number in repository **OR** supplier name, catalog number, clone number, and/**OR** RRID.	Yes	HeLa cells (wild type, homo sapiens, human, CVCL_0030)
**Primary cultures:** Provide species, strain, sex of origin, genetic modification status.	Not Applicable	Not Applicable
Report if the cell lines were recently **authenticated** (e.g., by STR profiling) and tested for mycoplasma contamination.	Not Applicable	Not Applicable

Experimental animals	Information included in the manuscript?	In which section is the information available? (Reagents and Tools Table, Materials and Methods, Figures, Data Availability Section)
**Laboratory animals or Model organisms:** Provide species, strain, sex, age, genetic modification status. Provide accession number in repository **OR** supplier name, catalog number, clone number, **OR** RRID.	Not Applicable	Not Applicable
**Animal observed in or captured from the field:** Provide species, sex, and age where possible.	Not Applicable	Not Applicable
Please detail **housing and husbandry conditions**.	Not Applicable	Not Applicable

Plants and microbes	Information included in the manuscript?	In which section is the information available? (Reagents and Tools Table, Materials and Methods, Figures, Data Availability Section)
**Plants:** provide species and strain, ecotype and cultivar where relevant, unique accession number if available, and source (including location for collected wild specimens).	Not Applicable	Not Applicable
**Microbes:** provide species and strain, unique accession number if available, and source.	Not Applicable	Not Applicable

Human research participants	Information included in the manuscript?	In which section is the information available? (Reagents and Tools Table, Materials and Methods, Figures, Data Availability Section)
If collected and within the bounds of privacy constraints report on age, sex and gender or ethnicity for all study participants.	Not Applicable	

Core facilities	Information included in the manuscript?	In which section is the information available? (Reagents and Tools Table, Materials and Methods, Figures, Data Availability Section)
If your work benefited from core facilities, was their service mentioned in the acknowledgments section?	Yes	Yes, Acknowledgements

Design

Study protocol	Information included in the manuscript?	In which section is the information available? (Reagents and Tools Table, Materials and Methods, Figures, Data Availability Section)
If study protocol has been **pre-registered, provide DOI in the manuscript**. For clinical trials, provide the trial registration number **OR** cite DOI.	Not Applicable	Not Applicable
Report the **clinical trial registration number** (at ClinicalTrials.gov or equivalent), where applicable.	Not Applicable	Not Applicable

Laboratory protocol	Information included in the manuscript?	In which section is the information available? (Reagents and Tools Table, Materials and Methods, Figures, Data Availability Section)
Provide DOI OR other citation details if **external detailed step-by-step protocols** are available.	Not Applicable	Not Applicable

Experimental study design and statistics	Information included in the manuscript?	In which section is the information available? (Reagents and Tools Table, Materials and Methods, Figures, Data Availability Section)
Include a statement about **sample size** estimate even if no statistical methods were used.	Yes	Material & Methods and Figure Legends; if statistcal methods were used at least n >3 was used. For single cell analysis, 476 were analysed without a predefined effect size.
Were any steps taken to minimize the effects of subjective bias when allocating animals/samples to treatment (e.g. **randomization procedure**)? If yes, have they been described?	Not Applicable	Not Applicable
Include a statement about **blinding** even if no blinding was done.	Not Applicable	Not Applicable
Describe **inclusion/exclusion criteria** if samples or animals were excluded from the analysis. Were the criteria pre-established?<br><br>If sample or data points were omitted from analysis, report if this was due to attrition or intentional exclusion and provide justification.	Yes	Material & Methods; single cells with identifications lower than 2x the standard deviation were filtered out.
For every figure, are **statistical tests** justified as appropriate? Do the data meet the assumptions of the tests (e.g., normal distribution)? Describe any methods used to assess it. Is there an estimate of variation within each group of data? Is the variance similar between the groups that are being statistically compared?	Yes	Yes, Material & Methods

Sample definition and in-laboratory replication	Information included in the manuscript?	In which section is the information available? (Reagents and Tools Table, Materials and Methods, Figures, Data Availability Section)
In the figure legends: state number of times the experiment was **replicated** in laboratory.	Yes	Yes
In the figure legends: define whether data describe **technical or biological replicates**.	Yes	Yes

Ethics

Ethics	Information included in the manuscript?	In which section is the information available? (Reagents and Tools Table, Materials and Methods, Figures, Data Availability Section)
Studies involving **human participants**: State details of **authority granting ethics approval** (IRB or equivalent committee(s), provide reference number for approval.	Yes	Material and Methods
Studies involving **human participants**: Include a statement confirming that **informed consent** was obtained from all subjects and that the experiments conformed to the principles set out in the WMA Declaration of Helsinki and the Department of Health and Human Services Belmont Report.	Yes	Material and Methods
Studies involving **human participants:** For publication of **patient photos**, include a statement confirming that consent to publish was obtained.	Not Applicable	Not Applicable
Studies involving experimental **animals**: State details of **authority granting ethics approval** (IRB or equivalent committee(s), provide reference number for approval. Include a statement of compliance with ethical regulations.	Not Applicable	Not Applicable
Studies involving **specimen and field samples:** State if relevant **permits** obtained, provide details of authority approving study; if none were required, explain why.	Yes	Material and Methods

Dual Use Research of Concern (DURC)	Information included in the manuscript?	In which section is the information available? (Reagents and Tools Table, Materials and Methods, Figures, Data Availability Section)
Could your study fall under dual use research restrictions? Please check biosecurity documents and list of **select agents and toxins** (CDC): https://www.selectagents.gov/sat/list.htm	Not Applicable	Not Applicable
If you used a select agent, is the security level of the lab appropriate and reported in the manuscript?	Not Applicable	Not Applicable
If a study is subject to dual use research of concern regulations, is the name of the **authority granting approval and reference number** for the regulatory approval provided in the manuscript?	Not Applicable	Not Applicable

Reporting

The MDAR framework recommends adoption of discipline-specific guidelines, established and endorsed through community initiatives. Journals have their own policy about requiring specific guidelines and recommendations to complement MDAR.

Adherence to community standards	Information included in the manuscript?	In which section is the information available? (Reagents and Tools Table, Materials and Methods, Figures, Data Availability Section)
State if relevant guidelines or checklists (e.g., **ICMJE, MIBBI, ARRIVE, PRISMA**) have been followed or provided.	Not Applicable	Not Applicable
For **tumor marker prognostic studies,** we recommend that you follow the **REMARK** reporting guidelines (see link list at top right). See author guidelines, under 'Reporting Guidelines'. Please confirm you have followed these guidelines.	Not Applicable	Not Applicable
For **phase II and III randomized controlled trials**, please refer to the **CONSORT** flow diagram (see link list at top right) and submit the CONSORT checklist (see link list at top right) with your submission. See author guidelines, under 'Reporting Guidelines'. Please confirm you have submitted this list.	Not Applicable	Not Applicable

Data Availability

Data availability	Information included in the manuscript?	In which section is the information available? (Reagents and Tools Table, Materials and Methods, Figures, Data Availability Section)
Have **primary datasets** been deposited according to the journal's guidelines (see 'Data Deposition' section) and the respective accession numbers provided in the Data Availability Section?	Yes	Yes, via PRIDE as indicated in the manuscript; section: Data availability.
Were **human clinical and genomic datasets** deposited in a public access-controlled repository in accordance to ethical obligations to the patients and to the applicable consent agreement?	Not Applicable	Not Applicable
Are **computational models** that are central and integral to a study available without restrictions in a machine-readable form? Were the relevant accession numbers or links provided?	Yes	Yes, via Github as indicated in the manuscript; section: Data availability.
If publicly available data were reused, provide the respective **data citations in the reference list**.	Yes	Yes
